# Identifying modifiable risk factors of lung cancer: Indications from Mendelian randomization

**DOI:** 10.1371/journal.pone.0258498

**Published:** 2021-10-18

**Authors:** Jie Ding, Zhenxing Tu, Hongquan Chen, Zhiguang Liu

**Affiliations:** 1 Cancer Center, The Affiliated Changzhou No.2 People’s Hospital of Nanjing Medical University, Changzhou, Jiangsu Province, China; 2 Department of Hand Surgery, The Second Hospital of Tangshan, Tangshan, Hebei Province, China; 3 Department of Bone Surgery, Affiliated Hospital of North China University of Science and Technology, Tangshan, Hebei Province, China; 4 Department of Pulmonary and Critical Care Medicine, Affiliated Changzhou Second People’s Hospital affiliated to Nanjing Medical University, Changzhou, Jiangsu Province, China; University of Hong Kong, HONG KONG

## Abstract

**Background:**

Lung cancer is the major cause of mortality in tumor patients. While its incidence rate has recently declined, it is still far from satisfactory and its potential modifiable risk factors should be explored.

**Methods:**

We performed a two-sample Mendelian randomization (MR) study to investigate the causal relationship between potentially modifiable risk factors (namely smoking behavior, alcohol intake, anthropometric traits, blood pressure, lipidemic traits, glycemic traits, and fasting insulin) and lung cancer. Besides, a bi-directional MR analysis was carried out to disentangle the complex relationship between different risk factors. Inverse-variance weighted (IVW) was utilized to combine the estimation for each SNP. Cochrane’s Q value was used to evaluate heterogeneity and two methods, including MR-Egger intercept and MR-PRESSO, were adopted to detect horizontal pleiotropy.

**Results:**

Three kinds of smoking behavior were all causally associated with lung cancer. Overall, smokers were more likely to suffer from lung cancer compared with non-smokers (OR = 2.58 [1.95, 3.40], p-value = 2.07 x 10^−11^), and quitting smoking could reduce the risk (OR = 4.29[2.60, 7.07], p-value = 1.23 x 10^−8^). Furthermore, we found a dose-response relationship between the number of cigarettes and lung cancer (OR = 6.10 [5.35, 6.96], p-value = 4.43x10^-161^). Lower HDL cholesterol could marginally increase the risk of lung cancer, but become insignificant after Bonferroni correction (OR = 0.82 [0.68, 1.00], p-value = 0.045). In addition, we noted no direct causal relationship between other risk factors and lung cancer. Neither heterogeneity nor pleiotropy was observed in this study. However, when treating the smoking behavior as the outcome, we found the increased BMI could elevate the number of cigarettes per day (beta = 0.139[0.104, 0.175], p-value = 1.99x10^-14^) and a similar effect was observed for the waist circumference and hip circumference. Additionally, the elevation of SBP could also marginally increase the number of cigarettes per day (beta = 0.001 [0.0002, 0.002], p-value = 0.018).

**Conclusion:**

Smoking behavior might be the most direct and effective modifiable way to reduce the risk of lung cancer. Meanwhile, smoking behavior can be affected by other risk factors, especially obesity.

## Introduction

Although its mortality rate has declined rapidly, lung cancer continues to be the leading cause of cancer death in the world [1]. Both unfavorable environmental factors (tobacco smoking, high intake of meat, alcohol intake, and air pollution, et al) and genetic susceptibility (*CHRNA3*, *CHRNA5*, *CHRNB4*, *TERT* and *CLPTM1L*, et al) contribute to its initiation and progression [1], and tobacco consumption has been well recognized as the most important risk factor [2]. Furthermore, two SNPs of *CLPTM1L* (rs401681 and rs402710) can influence susceptibility to lung cancer by regulating *TERT* expression in East Asian populations [3]. Meanwhile, the body mass index (BMI) is also inversely associated with lung cancer [4]. However, it was still far from satisfactory in terms of how we could reduce the incidence rate of lung cancer. Changes in metabolic patterns have been characterized in lung cancer, but it is still not known if endogenous metabolic biomarkers could increase lung cancer. Some observational studies reported that changes in lipid biomarkers could lead to the increased risk of lung cancer; and a U-shaped association was observed between total cholesterol (TC), triglycerides (TG), and lung cancer [5]. However, a recent meta-analysis indicated the null association between cholesterol intake and lung cancer [6]. Meanwhile, elevated insulin may increase the risk of lung cancer, but no other study has explored the impact of other glycemic traits on lung cancer [7]. Besides, it should be noted that observational studies can be easily biased by the confounders such as socioeconomic status and education attainment; and its results should be interpreted as association instead of causation. Thus, it is necessary to screen for risk factors of lung cancer using a relatively robust method.

Mendelian randomization (MR) is a causal inference method using genetic variants (usually single nucleotide polymorphism, SNP) as the instrumental variables to appraise the causation between exposure and outcome; and has achieved great success in determining the risk factors of diseases [8]. The genetic variants are randomly allocated at conception based on Mendel’s law and cannot be biased by potential confounders to some extent. Thus, it should be suitable for causal inference. With the rapid development of genome-wide association study (GWAS), the association between the genetic variant and human phenotype can be conveniently available. Nowadays, many MR studies (mainly two-sample MR studies) have been performed to detect the risk factors of lung cancer; and these risk factors included education [9], BMI [10], and some metabolic risk factors [11]. Therein, lower education might increase the risk of lung cancer [9], and a higher BMI could directly elevate the risk of small-cell lung cancer and squamous cell carcinoma while decreasing the risk of lung adenocarcinoma [10]. Meanwhile, a previous study has reported that higher fasting insulin could increase the risk of lung cancer [7,11]. These studies filled the gap between association and causation since MR studies could reduce these biases caused by unrecognized confounders, reverse causation, and measurement error [12]. However, these results still show a discrepancy and further analysis should be conducted. For instance, there has been disagreement on whether BMI was causally associated with lung adenocarcinoma Additionally, the relationship between blood pressure and lung cancer is inconsistent because the association between high blood pressure and lung cancer is either positive [13] or null [14]. Therefore, blood pressure should be included as a risk factor in our study and whether it can affect the risk of lung cancer should be determined.

Furthermore, we hope to include some new potential risk factors (blood pressure) that have not been investigated in MR studies considering that Shen et al has demonstrated the potential risk factors of lung cancer, including socioeconomic status, lifestyle, dietary, and obesity [15]. Meanwhile, we selected three types of smoking behavior (smoking initiation, smoking cessation, and the number of cigarettes per day) and tried to give novel insights into smoking’s impact on lung cancer. Here, we relied on publicly available GWAS summary statistics to detect modifiable factors that might cause lung cancer. These modifiable factors include tobacco consumption, alcohol intake, BMI, waist-hip-ratio (WHR), lipidemic and glycemic traits. The two-sample MR method was mainly implemented to detect causal factors and a bi-directional MR analysis was carried out to assess other risk factors’ effects on smoking, hoping to account for the inconsistency in observational studies and clarify the relationship between different types of smoking behavior and lung cancer.

## Methods

### Genetic instrumental variables for modifiable risk factors

All genetic instrumental variables were extracted from non-UK Biobank GWAS results with the largest sample size and they were clumped to get independent instrumental variables (linkage disequilibrium r^2^ < 0.01) using 1000 genome Phase 3 European samples as the reference panel (https://www.internationalgenome.org/) [16]. All instrumental variables hit the genome-wide significance (GWAS p-value < 5x10^-8^) except those from GWAS of 2 h after glucose challenge (2-h glucose) during an oral glucose tolerance test (OGTT) (GWAS p-value < 1x10^-5^) and each minor allele frequency was more than 0.01. Genetic variants associated with smoking behavior, namely smoking initiation, the number of cigarettes per day, and smoking cessation, were all obtained from the recent GWAS result derived from GWAS & Sequencing Consortium of Alcohol and Nicotine use (GSCAN) and the participants of GCSAN GWAS were all Europeans [17]. Smoking initiation phenotype was defined as having smoked (past or current) versus never smoked (non-smokers) and smoking cessation phenotype was defined as current versus past smokers. The number of cigarettes per day phenotype was classified into 5 categories: (1) less than a half pack, (2) a half pack, (3) 1 pack, (4) 2 packs, and (5) more than 2 packs. The alcohol instrumental variables were extracted from a habitual alcohol intake GWAS where the maximum habitual alcohol intake was defined as the largest number of drinks of alcohol (beer, wine, and/or liquor) one may have had in one day in a typical month [18]. GWAS results from the Genetic Investigation of ANthropometric Traits (GIANT) consortium were used to identify genetic variants associated with BMI, waist circumference, hip circumference, and waist-to-hip ratio (WHR) [19]. Since the hip circumference, waist circumference and WHR are closely correlated with BMI, we hope to explore their causal effect on lung cancer with adjustment of BMI. The adjustment has been performed by GINAT consortium and we used the summary statistics of hip circumference adjusted by BMI, waist circumference adjusted by BMI and WHR adjusted by BMI. The genetic variants associated with systolic blood pressure (SBP) and diastolic blood pressure (DBP) were extracted from a recent GWAS meta-analysis [20]. Genetic variants associated with lipid traits, including HDL, LDL, total cholesterol, and triglycerides, were obtained from the Global Lipids Genetics Consortium (GLGC) [21]. Results from the Meta-Analyses of Glucose and Insulin-related traits Consortium were used to extract instrumental variables for HbA1c [22], 2 hour glucose after oral glucose tolerance test (2-h glucose) [23], and fasting glucose and insulin [24]. All these GWASs have genomic control. Further details have been displayed in Table 1.

**Table 1:** A brief description of GWAS summary statistics for modifiable risk factors.

Risk factor	Consortium	Sample size	Covariates	PMID
Alcohol intake	GSCAN	126,936 Europeans + 17,029 non-Europeans	age, sex and first 10 genetic principal components	31151762
Smoking initiation	GSCAN	842,717 Europeans	age, gender, and the first 10 genetic principal components	33082346
Smoking cessation	GSCAN	842,717 Europeans	age, gender, and the first 10 genetic principal components	33082346
Cigarettes per day	GSCAN	842,717 Europeans	age, gender, and the first 10 genetic principal components	33082346
BMI	GIANT	344,369 Europeans	age, the first 15 genetic principal components, assessment center, and the genotyping chip	30778226
Hip circumference	GIANT	344,369 Europeans	age, the first 15 genetic principal components, assessment center, and the genotyping chip	30778226
Waist circumference	GIANT	344,369 Europeans	age, the first 15 genetic principal components, assessment center, and the genotyping chip	30778226
WHR	GIANT	344,369 Europeans	age, the first 15 genetic principal components, assessment center, and the genotyping chip	30778226
DBP	---	757,601 Europeans	sex, age, age^2^, BMI, and genotyping chips.	30224653
SBP	---	757,601 Europeans	sex, age, age^2^, BMI, and genotyping chips.	30224653
HDL cholesterol	GLGC	169,899 Europeans and 18,678 non-Europeans	age, age^2^, and sex	24097068
LDL cholesterol	GLGC	169,899 Europeans and 18,678 non-Europeans	age, age^2^, and sex	24097068
Total cholesterol	GLGC	169,899 Europeans and 18,678 non-Europeans	age, age^2^, and sex	24097068
Triglycerides	GLGC	169,899 Europeans and 18,678 non-Europeans	age, age^2^, and sex	24097068
HbA1c	MAGIC	159,940 Europeans	study-specific covariates	28898252
Fasting glucose	MAGIC	151,188 Europeans	age, sex, study site, and principal components	33402679
2-h glucose	MAGIC	15,234 Europeans	BMI, age, sex and study-specific covariates	20081857
Fasting insulin	MAGIC	105,056 Europeans	age, sex, study site, and principal components	33402679

Notes: PMID is the publication ID in PubMed. BMI is the body mass index; WHR is the waist-to-hip ratio; DBP is the diastolic blood pressure; SBP is the systolic blood pressure; HDL is the high-density lipoprotein; LDL is the low-density lipoprotein; 2-h glucose is the 2-hour glucose level of the oral glucose tolerance test.

### GWAS summary statistics for lung cancer

The genetic instrumental variable information on lung cancer was obtained from the most recent pan-cancer GWAS results with 2,485 European ancestry lung cancer cases and 410,350 European ancestry controls from two large cohorts the UK Biobank and Kaiser Permanente Genetic Epidemiology Research on Adult Health and Aging (GERA) cohorts genotyped by Affymetrix [7846216] [25]. With the Haplotype Reference Consortium (HRC) as the main reference panel and the merged UK 10K and 1000 Genomes phase 3 reference panels for additional data, the genotype imputation was performed and 93,095,623 SNPs were obtained. SNPs with low imputation quality (INFO < 0.3) and low allele frequency (MAF < 0.01) were excluded from the study. They included age, sex, the first 10 genetic principal components, and genotyping chips as the covariates, and this GWAS has genomic control.

### Mendelian randomization

Primarily, each modifiable risk factor was treated as the exposure to appraise its causal effect on lung cancer. Then, another MR analysis was carried out to estimate other risk factors’ effect on smoking, and a bi-directional MR was carried out to disentangle the complex relationship between obesity, alcohol intake and smoking considering their complicated effects on lung cancer.

**Fig 1:**
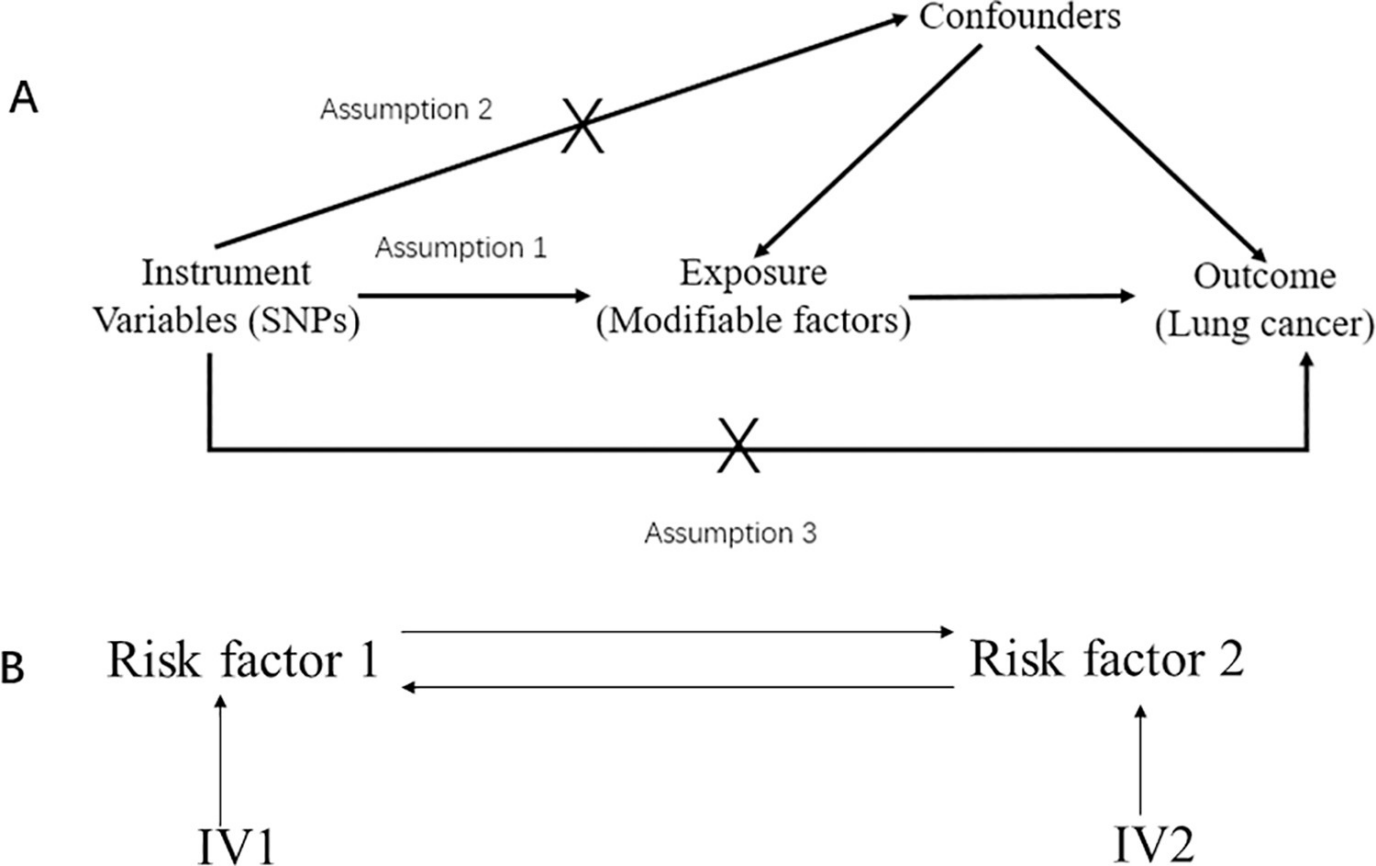
The basic principles underlying Mendelian randomization. Fig 1A is the three assumptions for Mendelian randomization analysis. Fig 1B is the basic principles of bi-directional Mendelian randomization. SNP is the single nucleotide polymorphism and IV is the instrumental variable.

MR is performed based on its three main principles: 1) The genetic variant was closely associated with the exposure; 2) The genetic variant was not associated with potential confounders; 3) The genetic variant was not associated with the outcome except via the way of exposure [8] (Fig 1). The first assumption can be tested by F statistics with the formula F = *β*^2^/*se*^2^. However, it is difficult to test the last two assumptions; and we can only use pleiotropy test to detect the violation of these two assumptions. When estimating the causal effect with IV analysis, additional assumptions should be satisfied, including linearity and no interactions between exposures and mediator [26]. Generally, the inverse-variance weighted (IVW) method was mainly used in estimating the genetic-driven causal effect of exposure on the outcome. Initially, we adopted the “MR-PRESSO” method to detect the outliers in the instrumental variables and removed them [27]; and then causal estimation was conducted. Bonferroni correction was used to adjust multiple testing (Bonferroni p-value < 0.05/18 = 2.77 x 10^−3^). Cochrane’s Q value was utilized to detect the heterogeneity in the linear regression and the MR-Egger intercept was used to test the horizontal pleiotropy [28], as a supplement to the “MR-PRESSO” global test [27]. MR Steiger test was employed to judge whether the SNP was more likely to be associated with the exposure than the outcome and we removed the SNP more associated with the outcome than the exposure [29]. Also, a leave-one-out sensitivity analysis was performed to find the driving genetic variants. If there was no heterogeneity or pleiotropy, the IVW estimation was adopted; and a random effect model was used when heterogeneity existed. The causal estimation would be the causal effect size if pleiotropy existed. Besides, the weighted median method was also performed as a supplement to IVW and MR-Egger methods implemented in R package “TwoSampleMR”.

All statistical analyses were conducted by R programming language (https://www.r-project.org/, version 4.0.0). The Power calculation was performed in mRnd (https://cnsgenomics.shinyapps.io/mRnd/).

## Results

**Fig 2:**
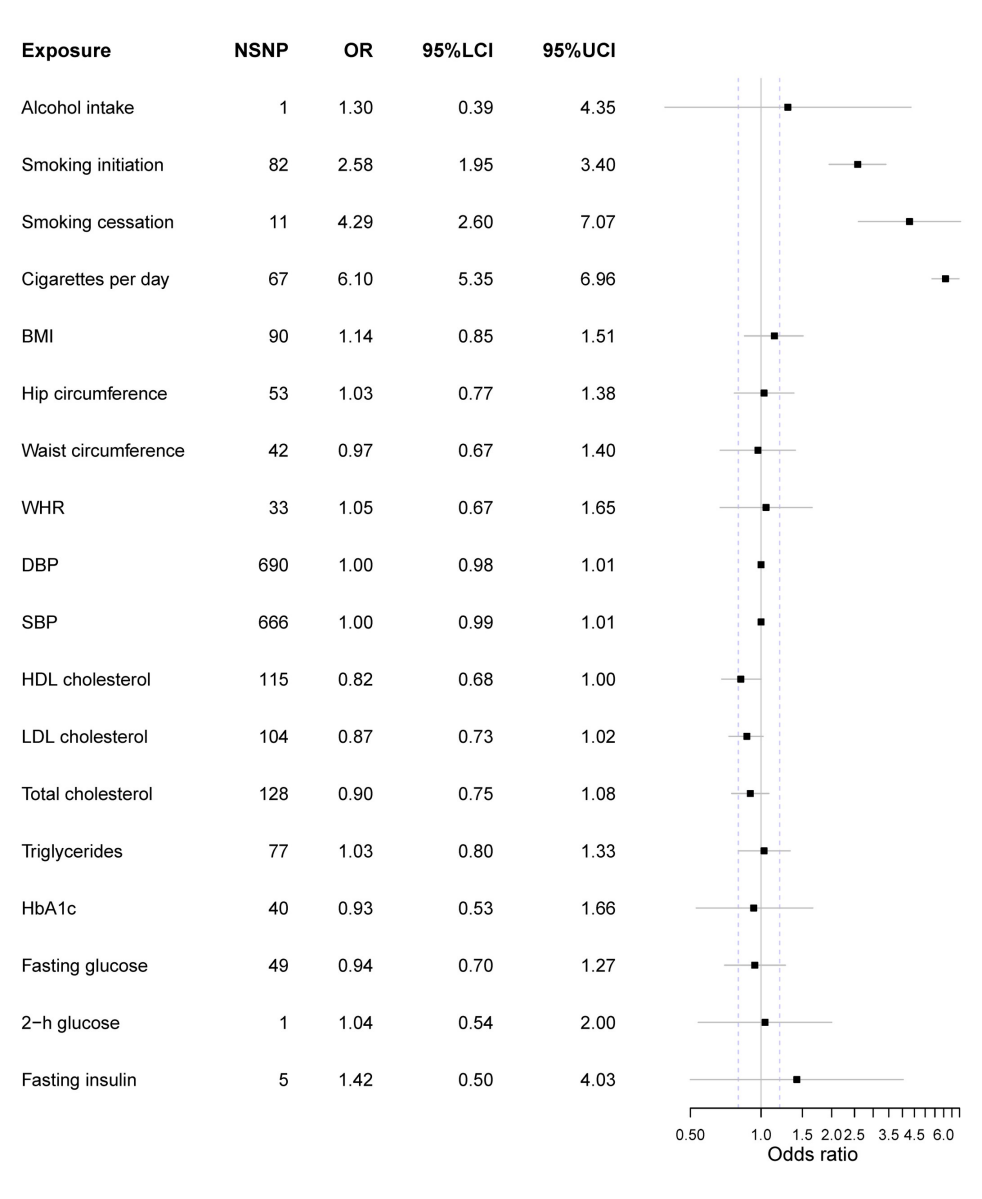
Forest plot of main MR results with lung cancer as the outcome. Exposure represents risk factors; NSNP is the number of SNPs used to estimate the causal effect size; OR is the odds ratio; 95%LCI is the lower limit of 95% confidence interval; 95%UCL is the upper limit of 95% confidence interval. BMI is the body mass index; WHR is the waist-to-hip ratio; DBP is the diastolic blood pressure; SBP is the systolic blood pressure; HDL is the high-density lipoprotein; LDL is the low-density lipoprotein; 2-h glucose is the 2-hour glucose level of the oral glucose tolerance test. The units of effect measures are per 1-SD increase for continuous exposures and 1-unit increase in log OR of binary exposures.

**Fig 3:**
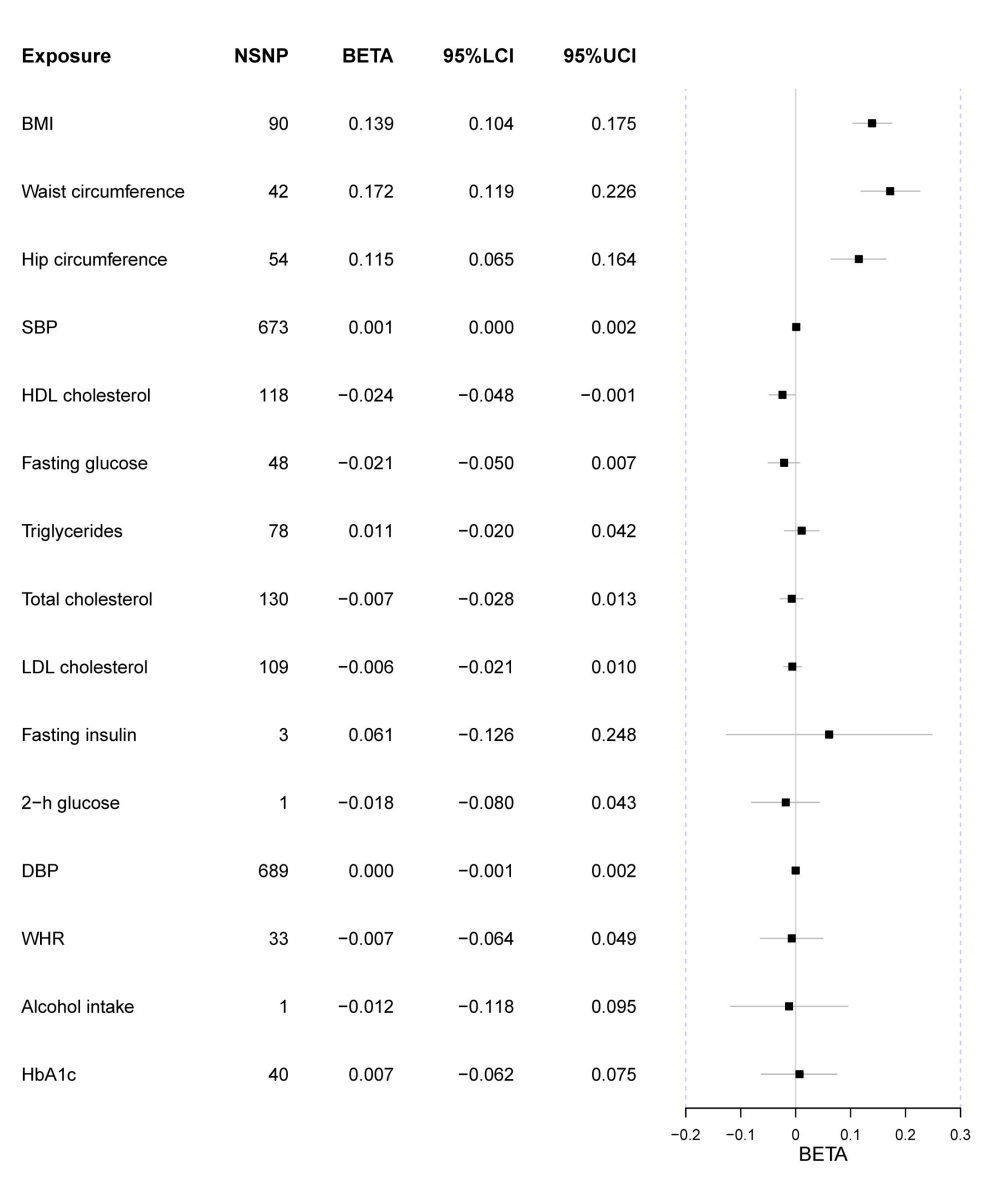
Forest plot of MR results treating the number of cigarettes per day as the outcome. Exposure represents risk factors; NSNP is the number of SNPs used to estimate the causal effect size; BETA is the effect size; 95%LCI is the lower limit of 95% confidence interval; 95%UCL is the upper limit of 95% confidence interval; BMI is the body mass index; WHR is the waist-to-hip ratio; DBP is the diastolic blood pressure; SBP is the systolic blood pressure; HDL is the high-density lipoprotein; LDL is the low-density lipoprotein; 2-h glucose is the 2-hour glucose level of the oral glucose tolerance test. The units of effect measures are per 1-SD increase for continuous exposures and 1-unit increase in log OR of binary exposures.

All genetic variants’ F statistics were more than 10, indicating there was less bias caused by potential weak instruments (S1–S18 Tables). Overall, only three kinds of smoking behavior were observed to directly causally increase the risk of lung cancer after Bonferroni correction (Fig 2). And a lower HDL level could marginally increase the risk of lung cancer. Besides, genetically-elevated BMI could increase the number of cigarettes per day (Fig 3).

### Alcohol intake & smoking-related exposures

Only 1 SNP rs1229984 located in *ADH1B* was qualified as the instrumental variable. No causal relationship was observed between habitual alcohol intake and lung cancer (OR = 1.30 [0.39, 4.35], p-value = 0.674). Also, no direct evidence supported that alcohol intake could alter the number of cigarettes per day (beta = -0.012 [-0.118, 0.095], p-value = 0.829).

**Fig 4:**
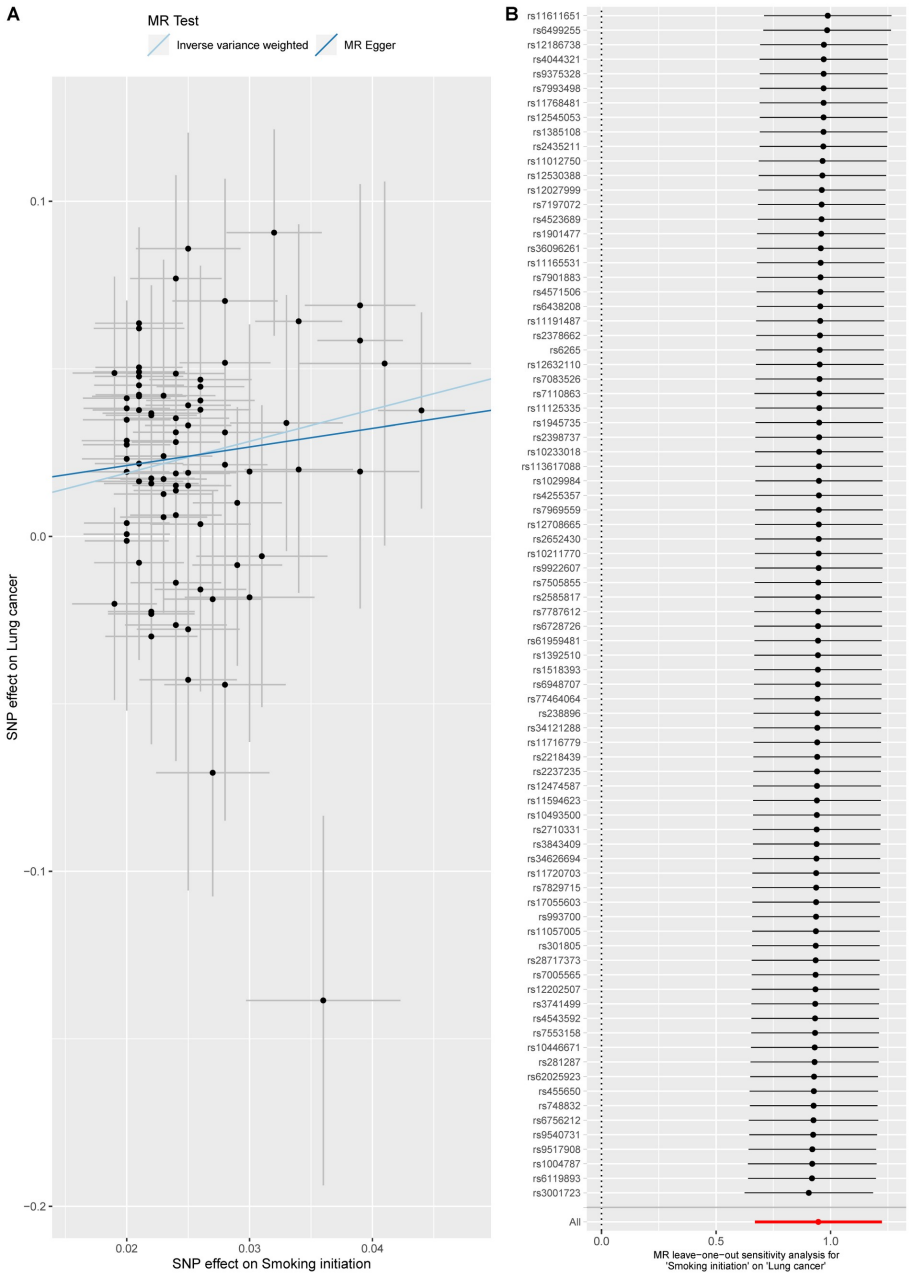
MR analysis of the effect of smoking initiation on lung cancer. Fig 4A is the scatter plot of the MR result. Fig 4B is the forest plot of the leave-one-out sensitivity result.

**Fig 5:**
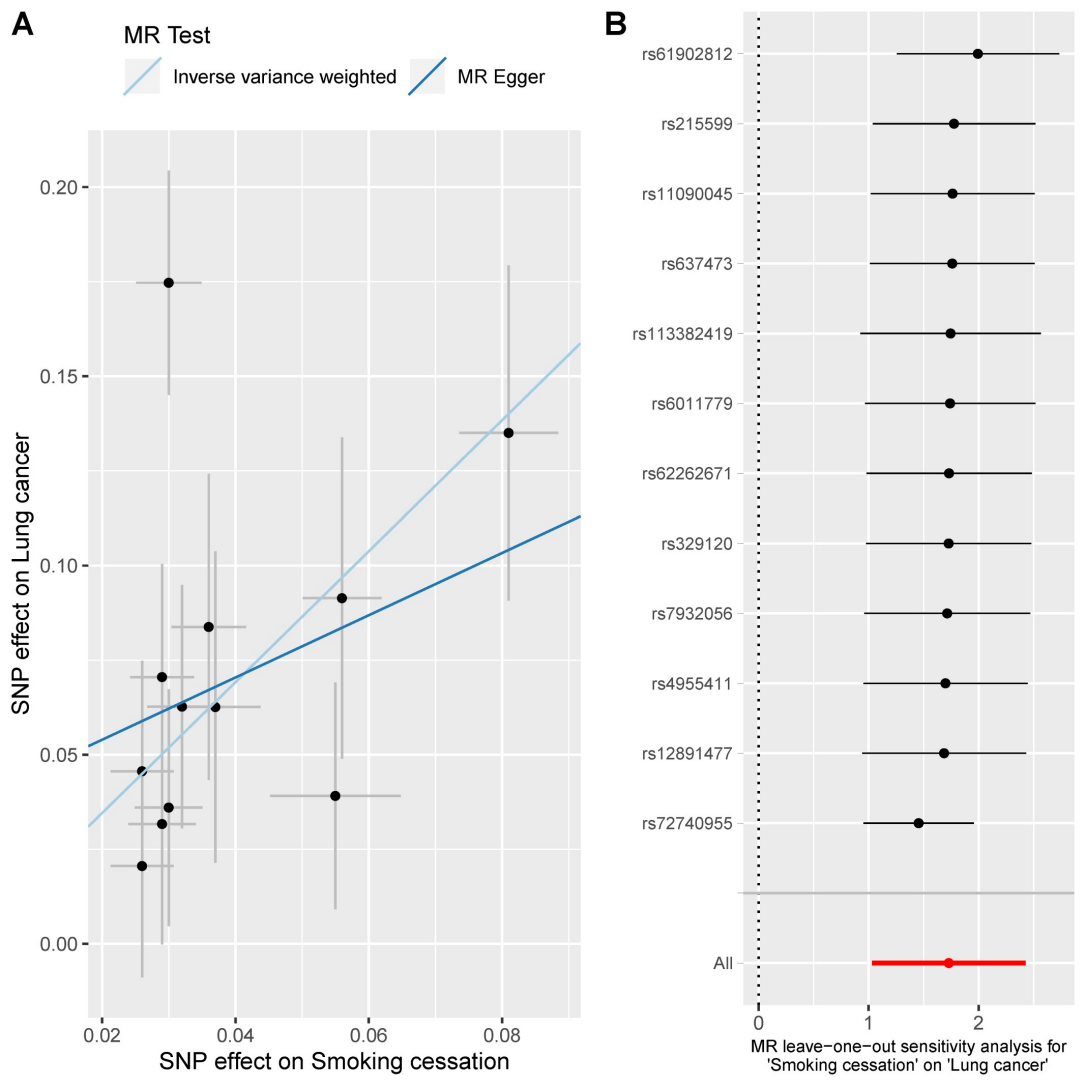
MR analysis of the effect of smoking cessation on lung cancer. Fig 5A is the scatter plot of the MR result. Fig 5B is the forest plot of the leave-one-out sensitivity result.

**Fig 6:**
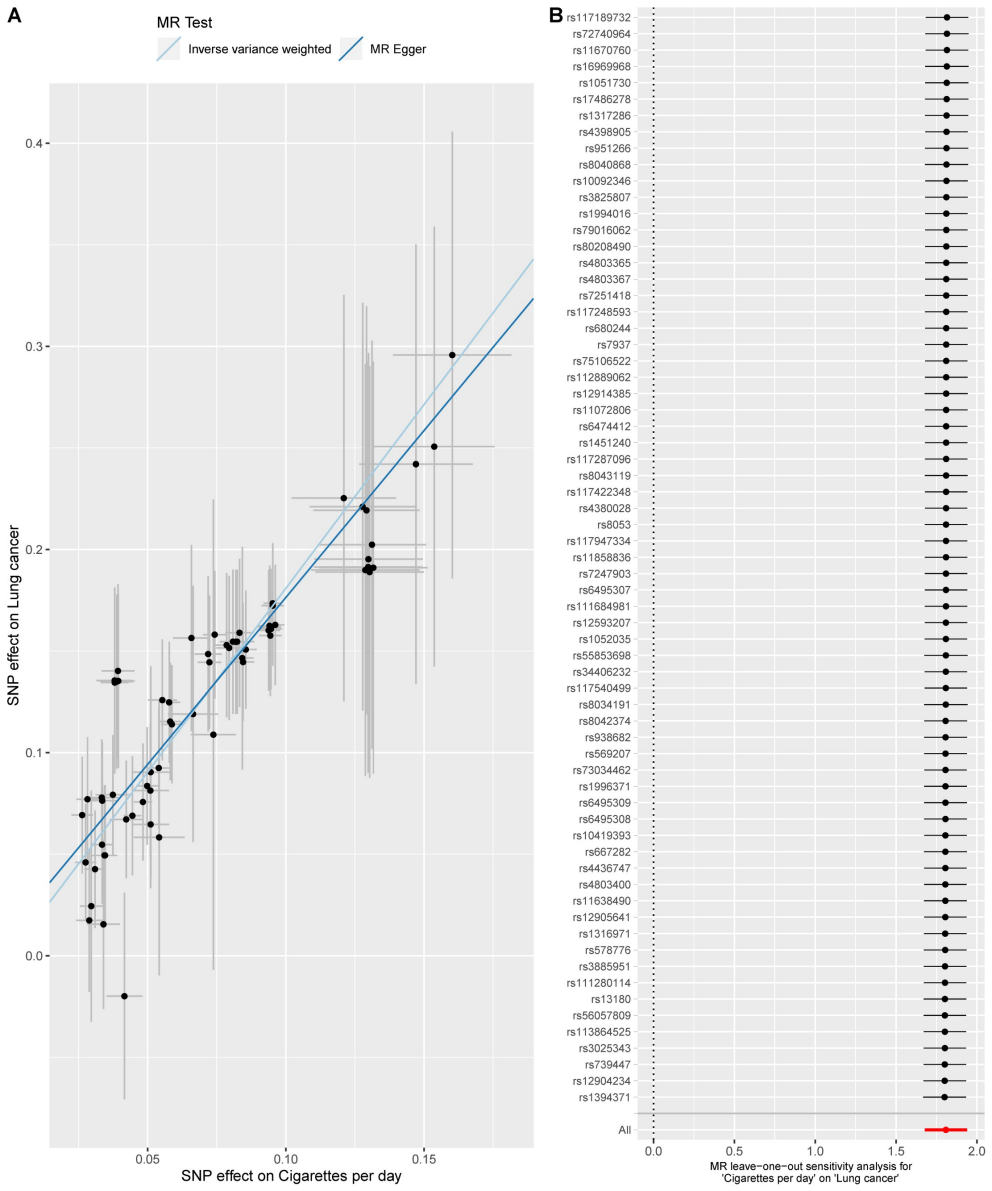
MR analysis of the effect of cigarettes per day of lung cancer. Fig 6A is the scatter plot of the MR result. Fig 6B is the forest plot of the leave-one-out sensitivity result.

Here, three smoking-related exposures were selected to appraise the causal effect of smoking on lung cancer. Overall, three exposures were all causally associated with lung cancer. For smoking initiation, the smokers were more likely to suffer from lung cancer compared with non-smokers (OR = 2.58 [1.95, 3.40], p-value = 2.07 x 10^−11^) (Fig 4). When analyzing smoking cessation, 2 SNPs (rs56113850 & rs72740955) were removed since they did not pass the outlier test and 1 SNP rs56113850 was excluded since it did not pass the leave-one-out sensitivity analysis; and the results indicated that those tended to have a higher risk of lung cancer if not quitting smoking (OR = 4.29 [2.60, 7.07], p-value = 1.23x10^-8^) (Fig 5). Besides, we found a dose-response relationship between the number of cigarettes and lung cancer (OR = 6.10 [5.35, 6.96], p-value = 4.43x10^-161^) (Fig 6). These results added evidence to the well-established causal relationship between smoking and lung cancer, indicating a necessity for quitting smoking. All MR-Egger intercept p-value and Cochrane’s p-value were more than 0.05.

### Anthropometric traits

In this study, we did not observe the causal relationship between BMI and lung cancer (OR = 1.14 [0.85, 1.51], p-value = 0.385) (S1 Fig). Also, neither heterogeneity nor pleiotropy was observed (Cochrane’s Q and MR-Egger intercept p-value > 0.05). For hip circumference, it might not lead to the change of lung cancer risk (OR = 1.03 [0.77, 1.38], p-value = 0.840) (S2 Fig) and the result was still insignificant after the adjustment of BMI (OR = 1.21 [0.93, 1.57], p-value = 0.151). Meanwhile, an increased waist circumference could not decrease the risk of lung cancer (OR = 0.97 [0.67, 1.40], p-value = 0.878) (S3 Fig), even after the adjustment of BMI (OR = 1.28 [0.90, 1.82], p-value = 0.177). Similarly, WHR was not causally associated with lung cancer, whether it was adjusted by BMI (OR = 1.11 [0.68, 1.83], p-value = 0.662) (S4 Fig) or not (OR = 1.05 [0.67, 1.65], p-value = 0.825).

However, we found BMI could increase the number of cigarettes per day (beta = 0.139 [0.104, 0.175], p-value = 1.99x10^-14^). Additionally, an increase of waist circumference and hip circumference could elevate the number of cigarettes per day as well (waist circumference beta = 0.172 [0.119, 0.226], p-value = 1.92x10^-10^; hip circumference beta = 0.115 [0.065, 0.164], p-value = 5.16x10^-6^).

Neither heterogeneity nor pleiotropy was detected in all these MR results (Cochrane’s Q p-value > 0.05; MR-Egger intercept p-value > 0.05; MR-PRESSO global test p-value > 0.05).

Overall, we did not observe the evidence that anthropometric traits, including BMI, waist circumference, hip circumference, and WHR, could directly affect the risk of lung cancer.

### Blood pressure

In our MR analysis, both SBP (OR = 1.00 [0.99, 1.01], p-value = 0.638) and DBP (OR = 1.00 [0.98, 1.01], p-value = 0.586) could not lead to the change of lung cancer risk (S5 and S6 Figs). When exploring the causal effect on smoking behavior, no significant results were observed after Bonferroni correction. However, the result indicated the elevation of SBP could increase the number of cigarettes per day (beta = 0.001 [0.0002, 0.002], p-value = 0.018). There was marginal heterogeneity in DBP (Cochrane’s p-value = 0.045) and we adopted the random effect model. Besides, we did not observe any heterogeneity and pleiotropy in other results (Cochrane’s Q p-value > 0.05; MR-Egger intercept p-value > 0.05; MR-PRESSO global test p-value > 0.05).

### Lipid traits

Four kinds of blood lipid levels were treated as exposures and the decreased HDL level was likely to increase the risk of lung cancer with a relatively marginal statistical significance (OR = 0.82 [0.68, 1.00], p-value = 0.045) (S7 Fig). Besides, LDL (OR = 0.87 [0.73, 1.02], p-value = 0.092), total cholesterol (OR = 0.90 [0.75, 1.08], p-value = 0.260) and triglycerides (OR = 1.03 [0.80,1.33], p-value = 0.824) were not causally associated with lung cancer (p-value > 0.05) (S8–S10 Figs). We did not observe significant results when treating the smoking behavior as the outcome after Bonferroni correction, but the HDL could marginally reduce the number of cigarettes per day (beta = -0.024 [-0.048, -0.001], p-value = 0.043). There was no heterogeneity and pleiotropy in these analyses (Cochrane’s Q p-value > 0.05; MR-Egger intercept p-value > 0.05; MR-PRESSO global test p-value > 0.05).

### Glycemic traits & fasting insulin

No causation was observed between glycemic traits and lung cancer, including HbA1c (OR = 0.93 [0.53, 1.66], p-value = 0.817) (S11 Fig) and fasting glucose (OR = 0.94 [0.70, 1.27], p-value = 0.681) (S12 Fig). Furthermore, a higher fasting insulin level could not lead to lung cancer (OR = 1.42 [0.50, 4.03], p-value = 0.514) (S13 Fig). Also, 2-h glucose could not change the risk of lung cancer (OR = 1.04 [0.54, 2.00], p-value = 0.911). No significant results were observed when treating the smoking behavior as the outcome. The fasting glucose result indicated the existence of heterogeneity (Cochrane’s Q p-value = 0.019) and a random effect model was employed to it. Besides, there was no pleiotropy or heterogeneity of other results (Cochrane’s Q p-value > 0.05; MR-Egger intercept p-value > 0.05; MR-PRESSO global test p-value > 0.05).

### Bidirectional MR between smoking, alcohol intake, and BMI

Considering the complex relationship between smoking, alcohol intake, and BMI, we have performed a bi-directional MR analysis for them. Our bi-directional MR suggested that alcohol intake could cause a higher BMI (beta = 0.197[0.135, 0.258], p-value = 4.59 x 10^−10^) and a higher BMI can increase the number of cigarettes per day (beta = 0.139[0.104, 0.175], p-value = 1.99 x 10^−14^). Besides, no other significant relationship was observed in this bi-directional analysis between these three risk factors. There was no pleiotropy or heterogeneity of other results (Cochrane’s Q p-value > 0.05; MR-Egger intercept p-value > 0.05; MR-PRESSO global test p-value > 0.05).

### Power calculation

The power of smoking initiation, smoking cessation, and the number of cigarettes per day were all 100%, indicating a sufficient statistical power for smoking behavior’s causal effect on lung cancer. The power for HDL is 0.69 and LDL is 0.57. Besides, no other exposure’s power was greater than 0.5.

## Discussion

In this MR study, we assessed the causal relationship between modifiable risk factors and lung cancer, hoping to help control this common cancer. These MR results indicated smoking was still the most risk factor for lung cancer. Besides, decreased level of HDL cholesterol level could marginally elevate the risk of lung cancer, but become insignificant after Bonferroni correction; and no other direct causal relationship was observed. Therein, we excluded the causal effect of blood pressure on lung cancer. When treating smoking behavior as the outcome, we found the BMI, waist circumference and hip circumference could increase the number of cigarettes per day, suggesting that genetic predisposition to obesity could increase the risk of smoking and further elevate the risk of lung cancer. However, we should take caution of it as there was no true total effect of risk factors on lung cancer except smoking. Additionally, SBP could marginally increase the number of cigarettes per day while HDL cholesterol might slightly decrease it.

The alcohol intake behavior is usually in strong correlation with smoking behavior and we are likely to observe the positive association between alcohol intake and lung cancer [1]. However, the association between alcohol consumption and lung cancer is still under debate for never-smokers [30,31]; and our results lend support for it that habitual alcohol intake cannot elevate the risk of lung cancer. Two reasons might help to account for it: (1) Only 1 genetic variant is available for this MR analysis and the statistical power is not sufficient; (2) The habitual alcohol intake cannot affect the risk of lung cancer if removing the impact of smoking. In our bi-directional MR analysis, we ruled out the causal relationship between alcohol intake and smoking, and alcohol intake cannot directly affect the risk of lung cancer. The previously reported association between alcohol intake and lung cancer might be confounded considering that alcohol intake could lead to a higher BMI.

As for smoking behavior, three subtypes of such behavior indicated smoking is the most perilous factor for lung cancer and there is a dose-response relationship between the number of cigarettes per day and lung cancer. Our results were consistent with previous MR studies [10,15]. Considering that Shen et al included the number of cigarettes per day in their study, the causal relationship between smoking cessation and lung cancer from our study indicated quitting smoking might be effective in reducing the risk of lung cancer. Thus, it is never late to quit smoking.

Obesity and related phenotypes were reported to be inversely associated with lung cancer and such phenomenon was called the “obesity paradox” [32]. Previous MR results unveiled that a higher BMI could increase the risk of lung squamous cell carcinoma, while not adenocarcinoma; and it could not increase the overall risk of lung cancer [11]. Our MR results lend strong support to it that a higher BMI could not affect the risk of lung cancer in enlarged sample size, but we could not validate the causal effect in subtypes of lung cancer due to data limits. BMI is an indicator for general obesity while WHR is for central obesity. Our results demonstrate that obesity might not directly elevate the risk of lung cancer no matter what the obesity type is. Since MR utilized genetic variants to evaluate the effect of risk factors on lung cancer, it could reduce the potential confounders to the largest extent; and the observed “obesity paradox” might be biased by potential confounders. In our bi-directional MR analysis between smoking, alcohol intake, and smoking, we found that obesity might alter smoking behavior and increase the number of cigarettes per day after Bonferroni correction. This conclusion was confirmed by Taylor et al where there was a positive causal effect of BMI on smoking [33]. Thus, we deemed that previously observed associations between obesity and lung cancer might be confounded by smoking.

A previous study indicated the association between increased blood pressure and lung cancer in men [34] while the most recent observational challenged this conclusion where blood pressure was not associated with lung cancer [14]. Our study firstly lent support to the latter using Mendelian randomization. We observed that the elevation of SBP could increase the number of cigarettes per day but it turned insignificant after the Bonferroni correction. Thus, we deemed that the previously observed association between blood pressure and lung cancer might be confounded by smoking behavior. Also, the conclusion should not be definite since we did not take sex differences in our MR study. Furthermore, these observational results might be biased due to their relatively small sample size. Overall, genetically elevated blood pressure might not increase the risk of lung cancer, but its sex-specific differences should be further investigated.

Our results indicate a very weak causal relationship between HDL cholesterol and lung cancer, and add evidence in previous observational studies where reduced lipid levels could elevate the risk of lung cancer [35]. Meanwhile, the elevation of HDL cholesterol could slightly decrease the number of cigarettes per day. However, such MR causal estimation becomes insignificant after Bonferroni correction. Our MR results for glycemic traits and fasting insulin did not observe any causal relationship. Such results seem to be inconsistent with previous observational and MR studies where the increased fasting insulin level could elevate the risk of lung cancer [11,36]. However, the direction of OR suggested high fasting insulin level could increase the risk of lung cancer, though not significant, suggesting a lack of statistical power of IVs for fasting insulin in our study. Thus, the association between fasting insulin and lung cancer should be further explored. The metabolic profile in lung cancer is much heterogenous and tumor cells can alter the original human metabolism [37]. Our MR results show that alteration of both lipid and glucose metabolism cannot increase the risk of lung cancer while not vice versa.

In our study, we performed a comprehensive MR to explore the risk factors for lung cancer and further identified risk factors for smoking behavior. Here, we strictly followed 3 assumptions for MR analysis and guaranteed the IV’s validity and power. Of the three MR assumptions, only the first 1 can be well satisfied and assumptions 2 and 3 cannot be fully met. We selected SNP reaching the genome-wide significance (p-value < 5x10^-8^) as the IV for the exposure and calculated the F statistics to appraise the power. However, assumption 2 cannot be fully tested since we cannot thoroughly rule out the association between IV and confounders. Thus, we performed the heterogeneity and pleiotropy test using Cochrane’s Q value, MR-Egger intercept, and MR-PRESSO, hoping to reduce the bias caused by assumption 2. As for assumption 3, we cannot judge whether a SNP can directly affect the outcome. Thus, we simply removed SNPs more likely to be associated with the outcome than the exposure using the MR Steiger test. However, several limitations should be pointed: (1) We cannot perform MR analysis on different subtypes of lung cancer due to data limitation; (2) We strictly selected the IVs, which might lower the statistical power; (3) We could not avoid the potential selection bias in evaluating lung cancer since individuals with multiple cancer diagnoses were classified as a case only for their first cancer. Thus, a case might suffer from more than 1 cancer and the lung cancer might be later than other cancer. Due to data limitation, we cannot obtain the individual-level data and cannot exactly appraise the potential selection bias in evaluating lung cancer due to its competing risk factors or missing genetic makeup during recruitment for exposures of interest; (4) Assumption 3 might be violated for binary exposures, but our conclusion might be robust considering that the number of cigarettes per day is a continuous variable for smoking behavior. It should be noted that the MR analysis tended to obtain more negative results than traditional observational studies since the 3 assumptions usually excluded more SNPs and lowered the statistical power. Thus, we still cannot rule out other risk factors’ effects on lung cancer.

In conclusion, our MR results indicate smoking behavior might be the sole effective modifiable way to reduce the risk of lung cancer considering three types of smoking behavior were all causally associated with lung cancer, and quitting smoking could lower the risk. Besides, the genetic liability to obesity can increase the risk of smoking. This MR study suggested the effects of other risk factors on smoking, indicating previously observed associations between risk factors and lung cancer might be confounded by smoking, such as blood pressure and HbA1c. Our study indicated smoking is the most perilous factor for lung cancer, further strengthening the need for tobacco control.

## Supporting information

S1 Fig MR analysis for the effect of BMI on lung cancer. A is the scatter plot of MR result. B is the forest plot of leave-one-out sensitivity result.
(PDF)

S2 Fig MR analysis for the effect of hip circumference on lung cancer. A is the scatter plot of MR result of the effect of hip circumference on lung cancer. B is the forest plot of leave-one-out sensitivity result.
(PDF)

S3 Fig MR analysis for the effect of waist circumference on lung cancer. A is the scatter plot of MR result of the effect of waist circumference on lung cancer. B is the forest plot of leave-one-out sensitivity result.
(PDF)

S4 Fig MR analysis for the effect of WHR on lung cancer. A is the scatter plot of MR result of the effect of WHR on lung cancer. B is the forest plot of leave-one-out sensitivity result.
(PDF)

S5 Fig MR analysis for the effect of DBP on lung cancer. A is the scatter plot of MR result for the effect of DBP on lung cancer. B is the forest plot of leave-one-out sensitivity result.
(PDF)

S6 Fig MR analysis for the effect of SBP on lung cancer. A is the scatter plot of MR result for the effect of SBP on lung cancer. B is the forest plot of leave-one-out sensitivity result.
(PDF)

S7 Fig MR analysis for the effect of HDL cholesterol on lung cancer. A is the scatter plot of MR result for the effect of HDL cholesterol on lung cancer. B is the forest plot of leave-one-out sensitivity result.
(PDF)

S8 Fig MR analysis for the effect of LDL cholesterol on lung cancer. A is the scatter plot of MR result for the effect of LDL cholesterol on lung cancer. B is the forest plot of leave-one-out sensitivity result.
(PDF)

S9 Fig MR analysis for the effect of total cholesterol on lung cancer. A is the scatter plot of MR result for the effect of total cholesterol on lung cancer. B is the forest plot of leave-one-out sensitivity result.
(PDF)

S10 Fig MR analysis for the effect of triglycerides on lung cancer. A is the scatter plot of MR result for the effect of triglycerides on lung cancer. B is the forest plot of leave-one-out sensitivity result.
(PDF)

S11 Fig MR analysis for the effect of HbA1c on lung cancer. A is the scatter plot of MR result for the effect of HbA1c on lung cancer. B is the forest plot of leave-one-out sensitivity result.
(PDF)

S12 Fig MR analysis for the effect of fasting glucose on lung cancer. A is the scatter plot of MR result for the effect of fasting glucose on lung cancer. B is the forest plot of leave-one-out sensitivity result.
(PDF)

S13 Fig MR analysis for the effect of fasting insulin on lung cancer. A is the scatter plot of MR result for the effect of fasting insulin on lung cancer. B is the forest plot of leave-one-out sensitivity result.
(PDF)

S1 Table Instrumental variables of alcohol intake. The SNP is the result of genetic variants; A1 is the effect allele; A2 is the other allele; beta is the effect size of A1 on the exposure; she is the standard error of beta; pval is the p-value of beta; F is the F statistics.
(PDF)

S2 Table Instrumental variables of smoking initiation. The SNP is the result of genetic variants; A1 is the effect allele; A2 is the other allele; beta is the effect size of A1 on the exposure; she is the standard error of beta; pval is the p-value of beta; F is the F statistics.
(PDF)

S3 Table Instrumental variables of smoking cessation. The SNP is the result of genetic variants; A1 is the effect allele; A2 is the other allele; beta is the effect size of A1 on the exposure; she is the standard error of beta; pval is the p-value of beta; F is the F statistics.
(PDF)

S4 Table Instrumental variables of cigarettes per day. The SNP is the result of genetic variants; A1 is the effect allele; A2 is the other allele; beta is the effect size of A1 on the exposure; she is the standard error of beta; pval is the p-value of beta; F is the F statistics.
(PDF)

S5 Table Instrumental variables of BMI. The SNP is the result of genetic variants; A1 is the effect allele; A2 is the other allele; beta is the effect size of A1 on the exposure; she is the standard error of beta; pval is the p-value of beta; F is the F statistics.
(PDF)

S6 Table Instrumental variables of hip circumference. The SNP is the result of genetic variants; A1 is the effect allele; A2 is the other allele; beta is the effect size of A1 on the exposure; she is the standard error of beta; pval is the p-value of beta; F is the F statistics.
(PDF)

S7 Table Instrumental variables of waist circumference. The SNP is the result of genetic variants; A1 is the effect allele; A2 is the other allele; beta is the effect size of A1 on the exposure; she is the standard error of beta; pval is the p-value of beta; F is the F statistics.
(PDF)

S8 Table Instrumental variables of WHR. The SNP is the result of genetic variants; A1 is the effect allele; A2 is the other allele; beta is the effect size of A1 on the exposure; she is the standard error of beta; pval is the p-value of beta; F is the F statistics.
(PDF)

S9 Table Instrumental variables of DBP. The SNP is the result of genetic variants; A1 is the effect allele; A2 is the other allele; beta is the effect size of A1 on the exposure; she is the standard error of beta; pval is the p-value of beta; F is the F statistics.
(PDF)

S10 Table Instrumental variables of SBP. The SNP is the result of genetic variants; A1 is the effect allele; A2 is the other allele; beta is the effect size of A1 on the exposure; she is the standard error of beta; pval is the p-value of beta; F is the F statistics.
(PDF)

S11 Table Instrumental variables of HDL cholesterol. The SNP is the result of genetic variants; A1 is the effect allele; A2 is the other allele; beta is the effect size of A1 on the exposure; she is the standard error of beta; pval is the p-value of beta; F is the F statistics.
(PDF)

S12 Table Instrumental variables of LDL cholesterol. The SNP is the result of genetic variants; A1 is the effect allele; A2 is the other allele; beta is the effect size of A1 on the exposure; she is the standard error of beta; pval is the p-value of beta; F is the F statistics.
(PDF)

S13 Table Instrumental variables of total cholesterol. The SNP is the result of genetic variants; A1 is the effect allele; A2 is the other allele; beta is the effect size of A1 on the exposure; she is the standard error of beta; pval is the p-value of beta; F is the F statistics.
(PDF)

S14 Table Instrumental variables of triglycerides. The SNP is the result of genetic variants; A1 is the effect allele; A2 is the other allele; beta is the effect size of A1 on the exposure; she is the standard error of beta; pval is the p-value of beta; F is the F statistics.
(PDF)

S15 Table Instrumental variables of HbA1c. The SNP is the result of genetic variants; A1 is the effect allele; A2 is the other allele; beta is the effect size of A1 on the exposure; she is the standard error of beta; pval is the p-value of beta; F is the F statistics.
(PDF)

S16 Table Instrumental variables of fasting glucose. The SNP is the result of genetic variants; A1 is the effect allele; A2 is the other allele; beta is the effect size of A1 on the exposure; she is the standard error of beta; pval is the p-value of beta; F is the F statistics.
(PDF)

S17 Table Instrumental variables of 2-h glucose. The SNP is the result of genetic variants; A1 is the effect allele; A2 is the other allele; beta is the effect size of A1 on the exposure; she is the standard error of beta; pval is the p-value of beta; F is the F statistics.
(PDF)

S18 Table Instrumental variables of fasting insulin. The SNP is the result of genetic variants; A1 is the effect allele; A2 is the other allele; beta is the effect size of A1 on the exposure; she is the standard error of beta; pval is the p-value of beta; F is the F statistics.
(PDF)
