# Supplementary figures and images for: Identifying modifiable risk factors of lung cancer: Indications from Mendelian randomization
Source: PLoS One. 2021 Oct 18;16(10):e0258498. doi: 10.1371/journal.pone.0258498 (PMC8523078; doi:10.1371/journal.pone.0258498)

**A**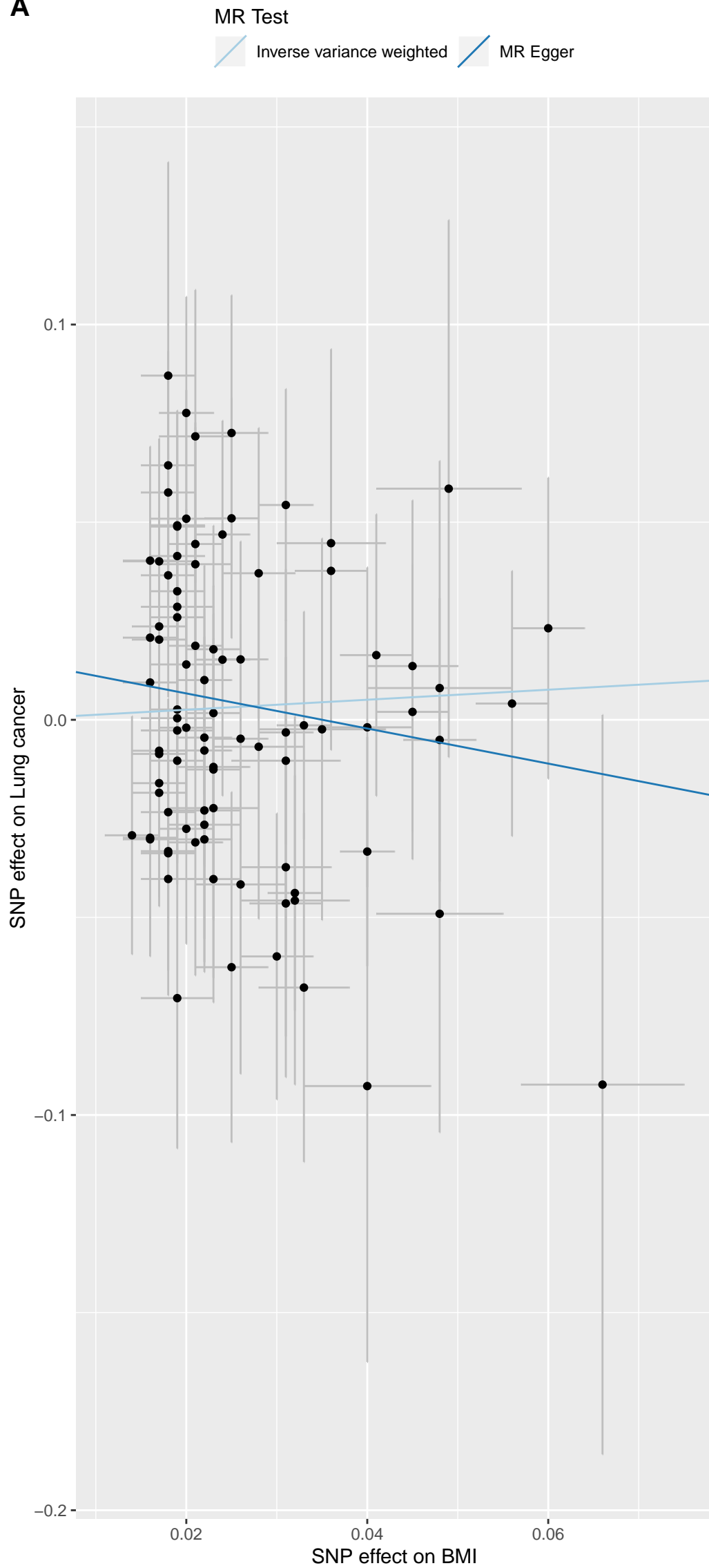**B**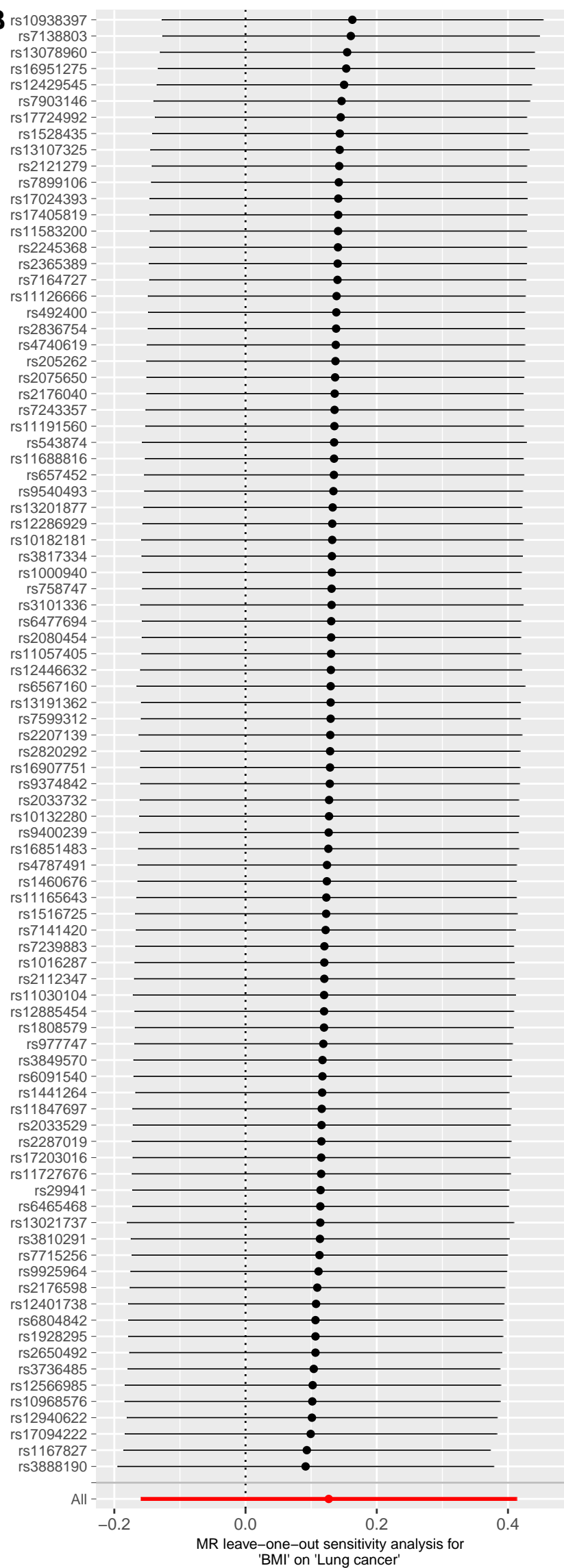

Supplement: S1 Fig — A is the scatter plot of MR result. B is the forest plot of leave-one-out sensitivity result. (PDF) [file pone.0258498.s001.pdf]

**A**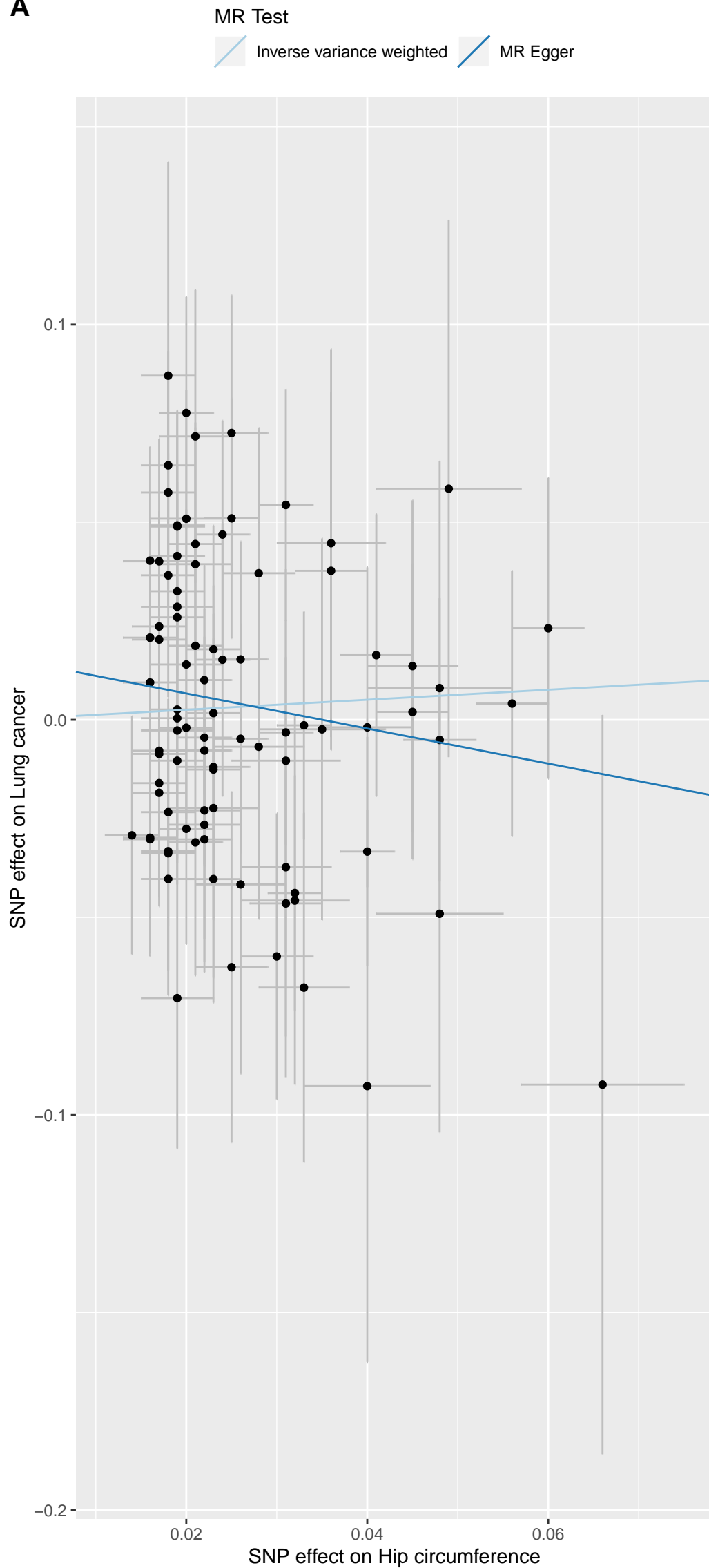**B**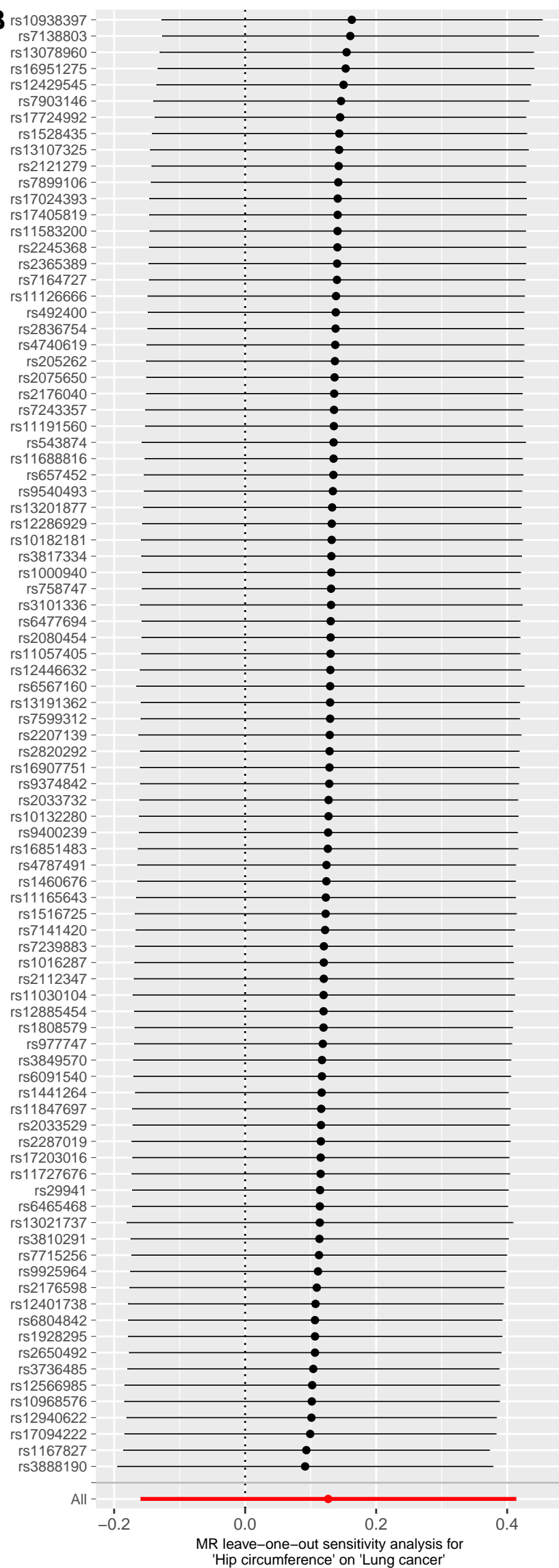

Supplement: S2 Fig — A is the scatter plot of MR result of the effect of hip circumference on lung cancer. B is the forest plot of leave-one-out sensitivity result. (PDF) [file pone.0258498.s002.pdf]

**A**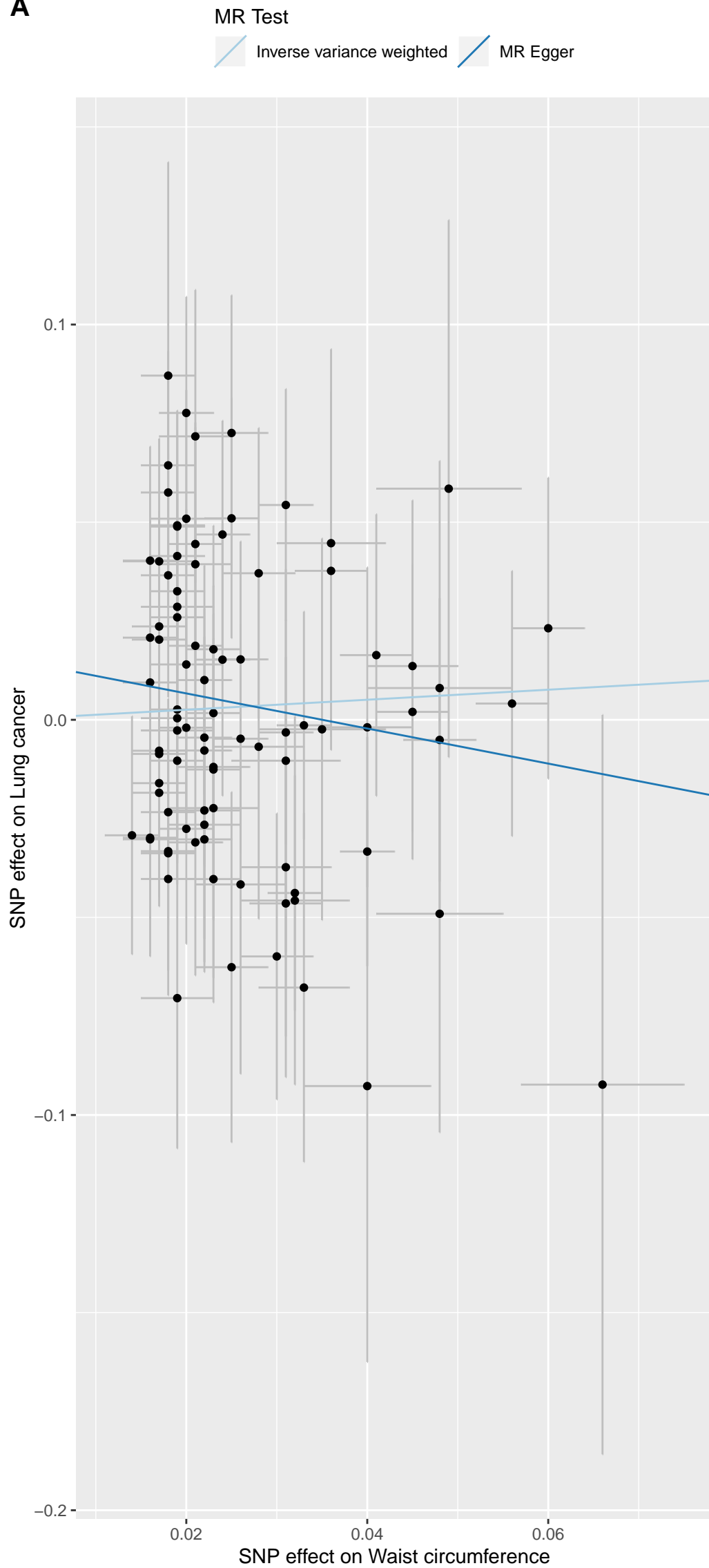**B**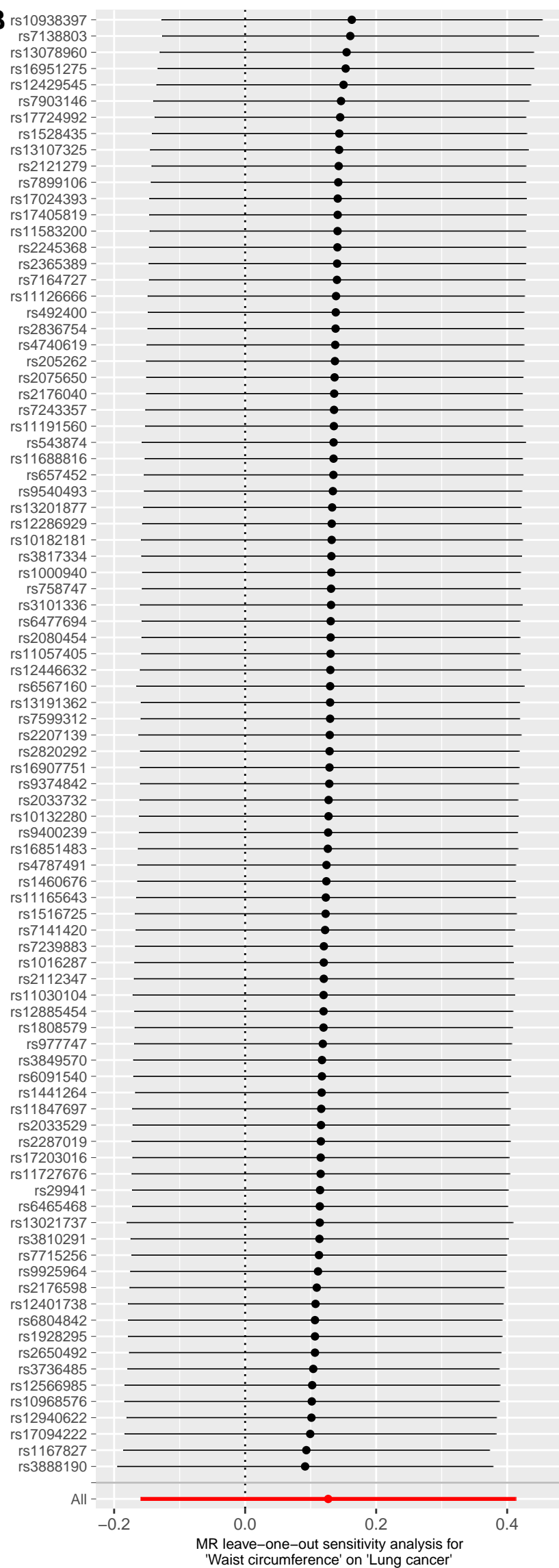

Supplement: S3 Fig — A is the scatter plot of MR result of the effect of waist circumference on lung cancer. B is the forest plot of leave-one-out sensitivity result. (PDF) [file pone.0258498.s003.pdf]

**A**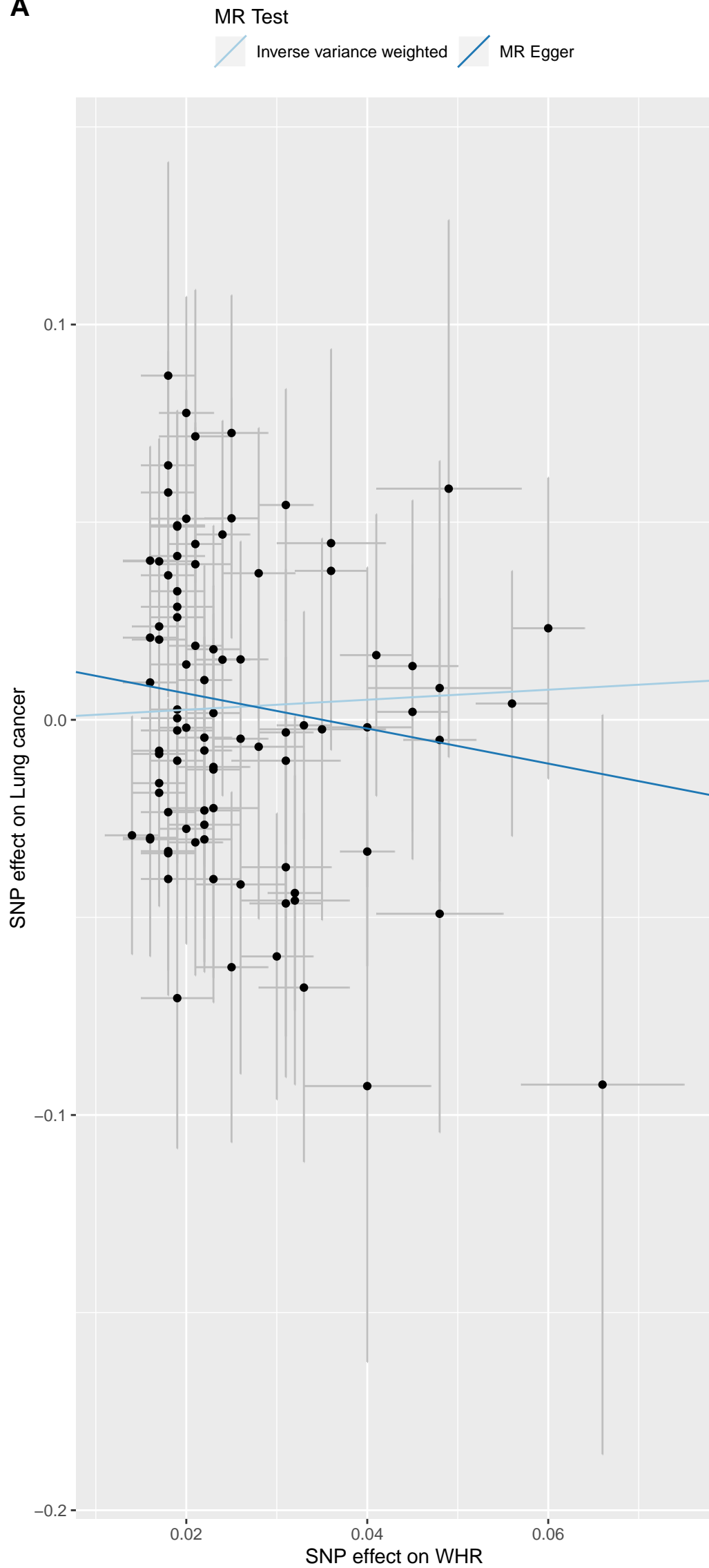**B**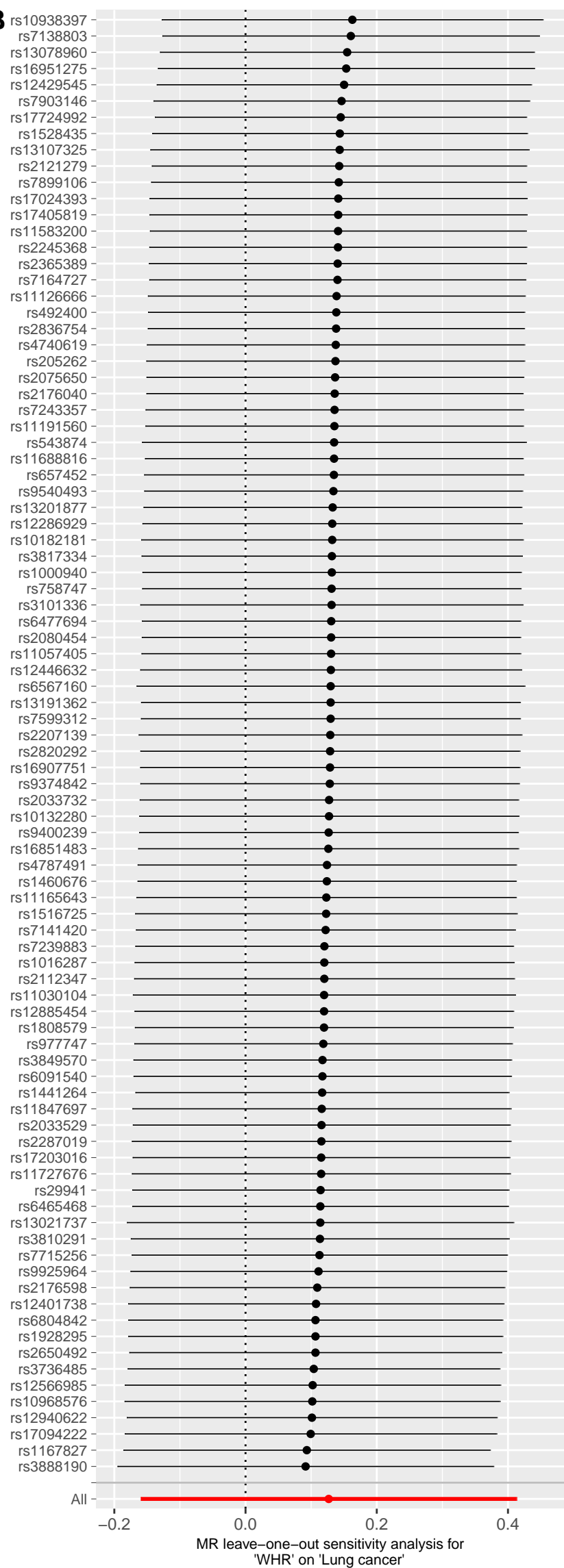

Supplement: S4 Fig — A is the scatter plot of MR result of the effect of WHR on lung cancer. B is the forest plot of leave-one-out sensitivity result. (PDF) [file pone.0258498.s004.pdf]

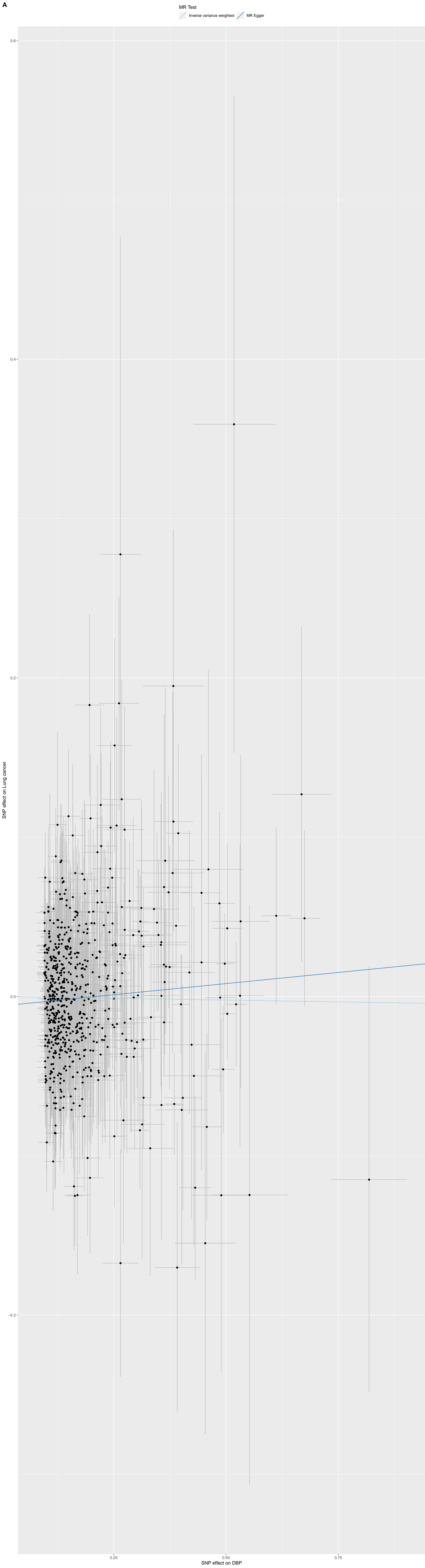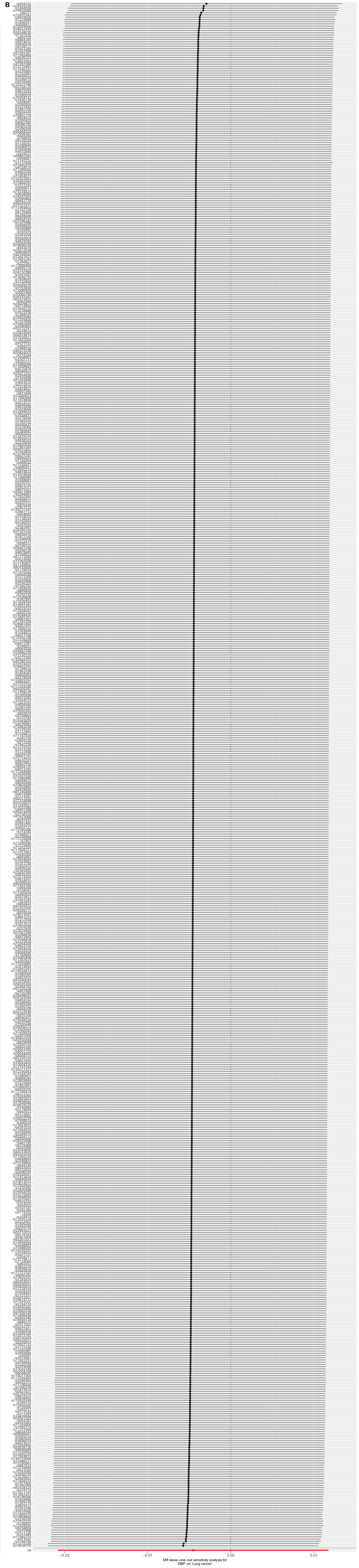

Supplement: S5 Fig — A is the scatter plot of MR result for the effect of DBP on lung cancer. B is the forest plot of leave-one-out sensitivity result. (PDF) [file pone.0258498.s005.pdf]

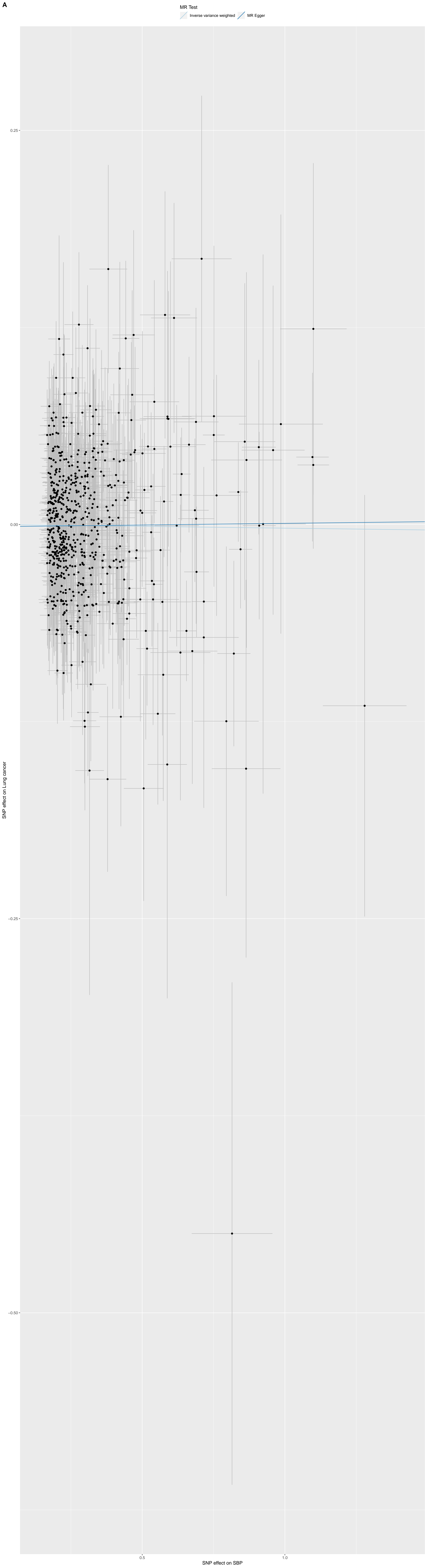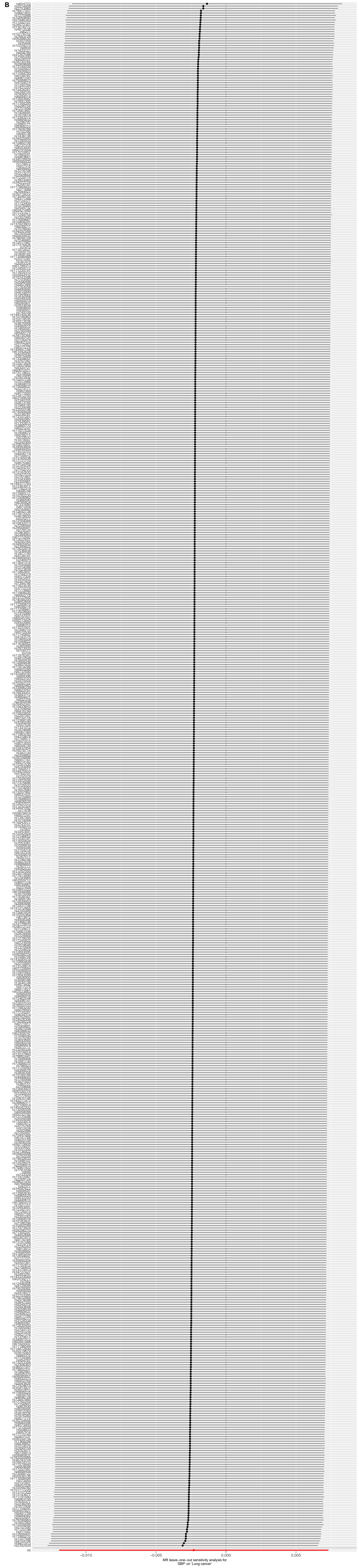

Supplement: S6 Fig — A is the scatter plot of MR result for the effect of SBP on lung cancer. B is the forest plot of leave-one-out sensitivity result. (PDF) [file pone.0258498.s006.pdf]

**A**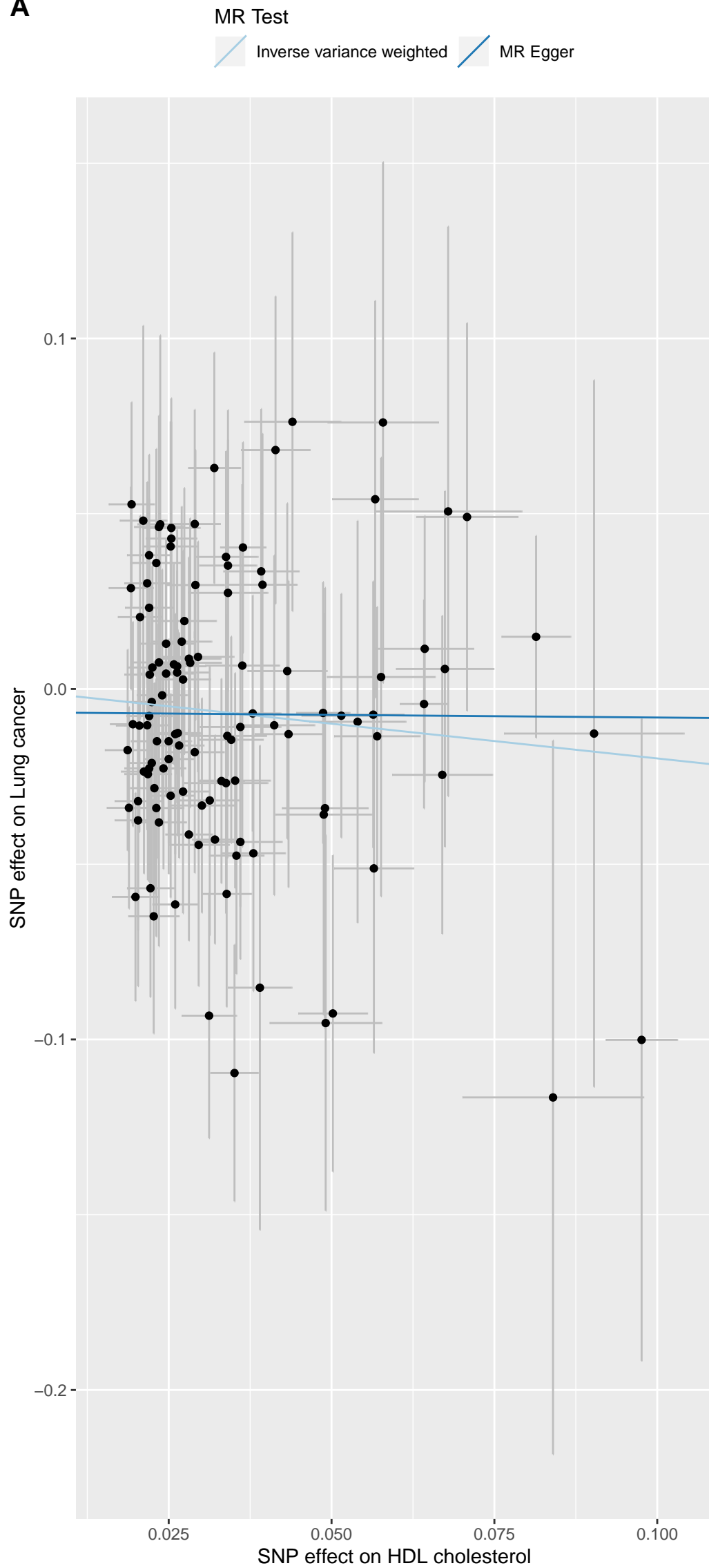**B**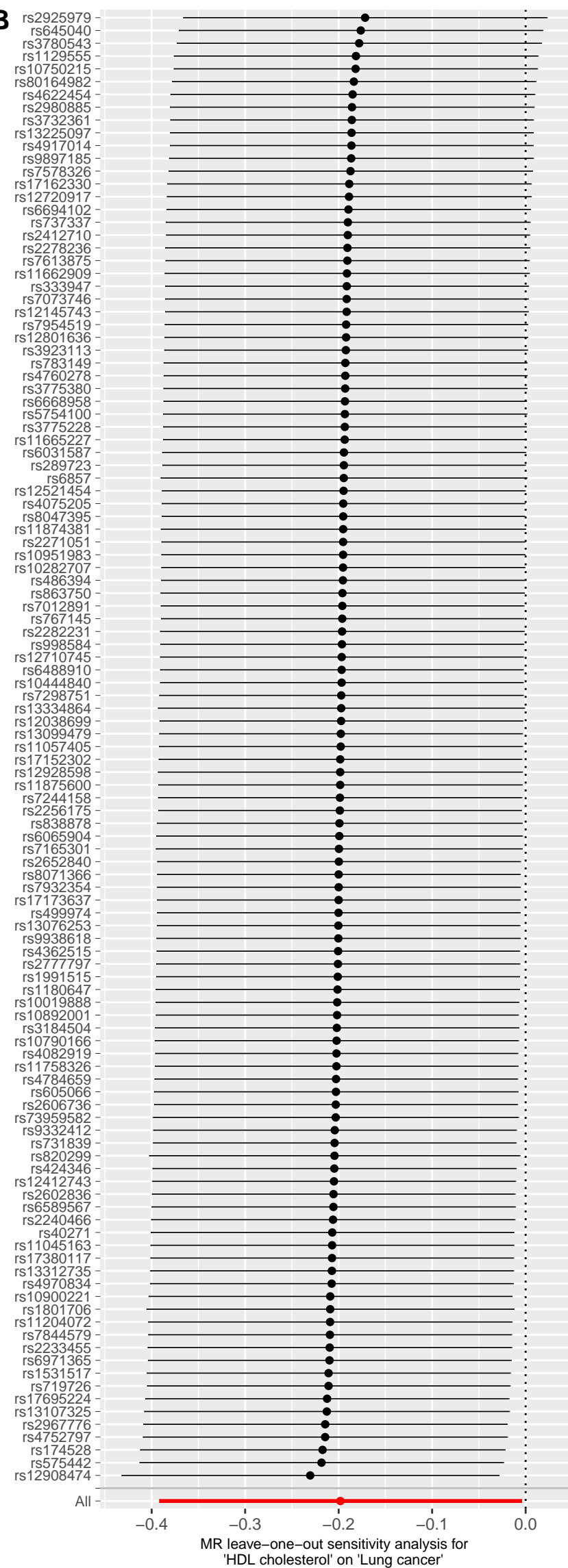

Supplement: S7 Fig — A is the scatter plot of MR result for the effect of HDL cholesterol on lung cancer. B is the forest plot of leave-one-out sensitivity result. (PDF) [file pone.0258498.s007.pdf]

**A**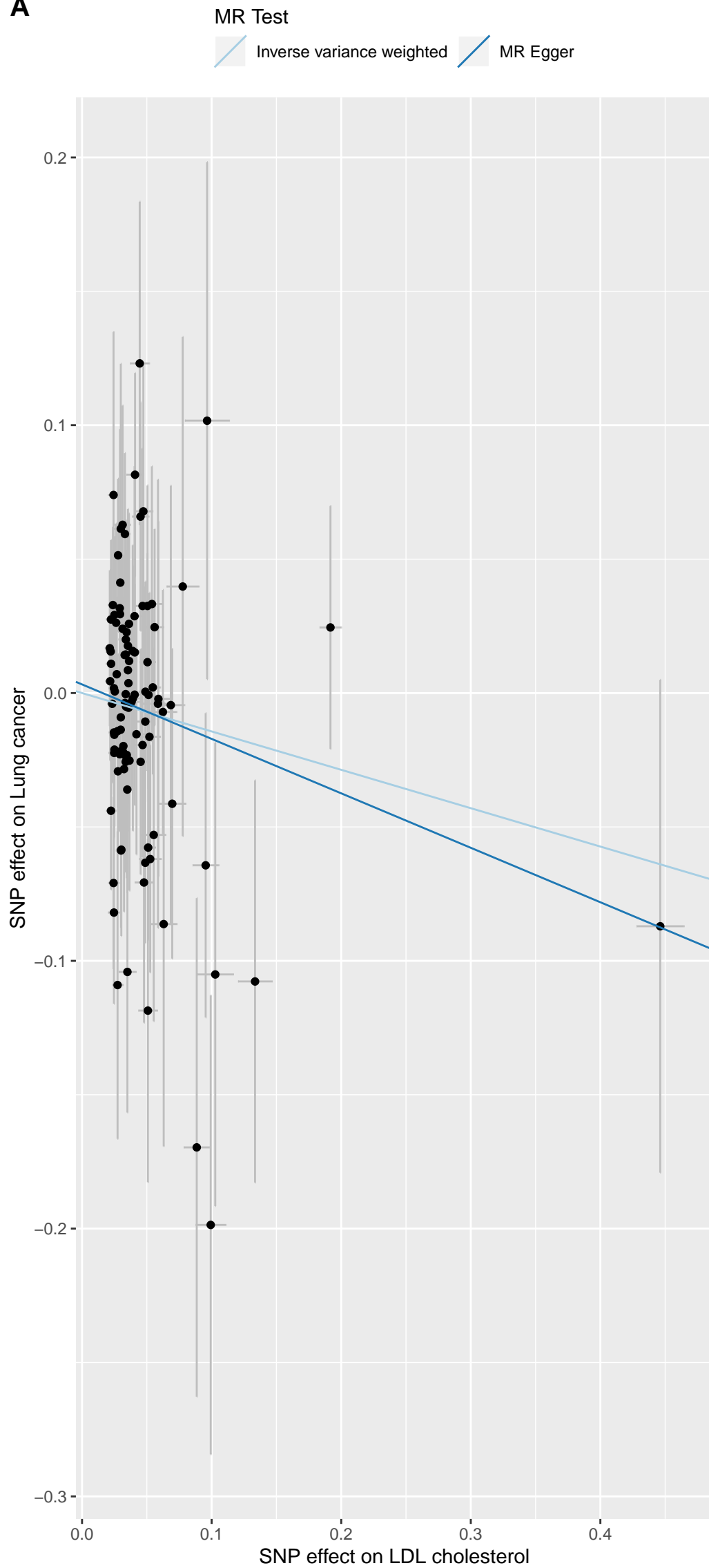**B**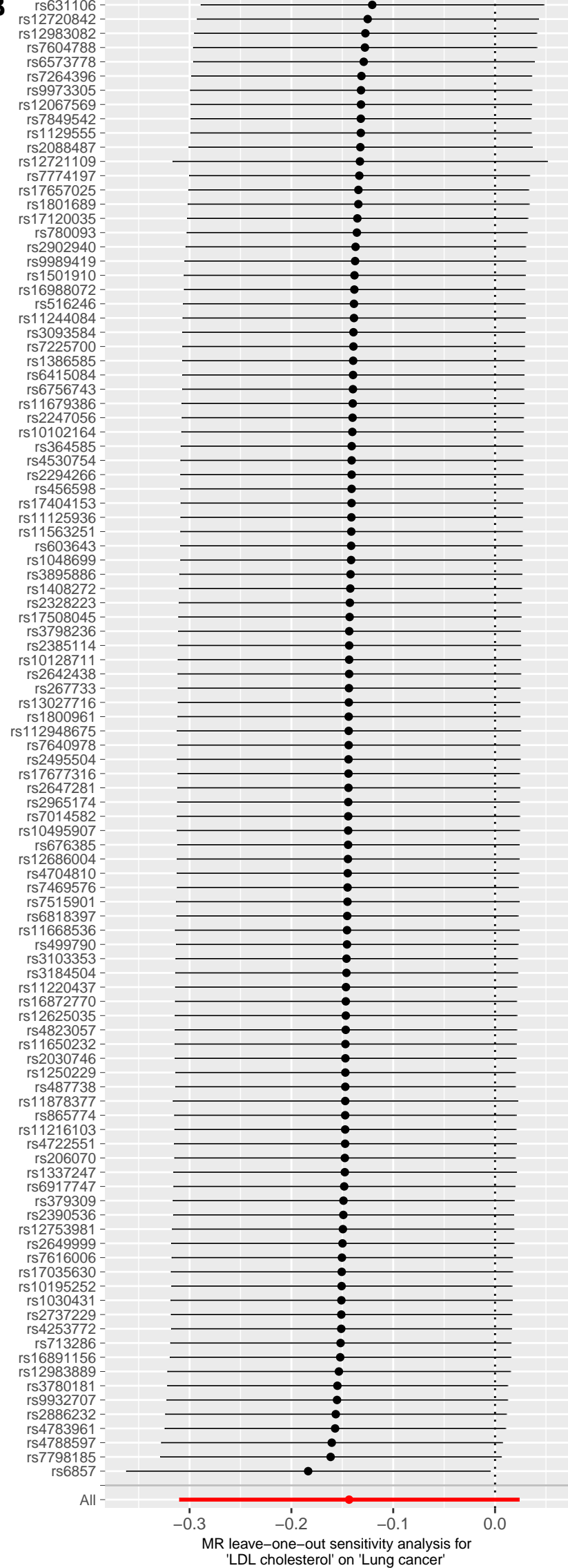

Supplement: S8 Fig — A is the scatter plot of MR result for the effect of LDL cholesterol on lung cancer. B is the forest plot of leave-one-out sensitivity result. (PDF) [file pone.0258498.s008.pdf]

**A**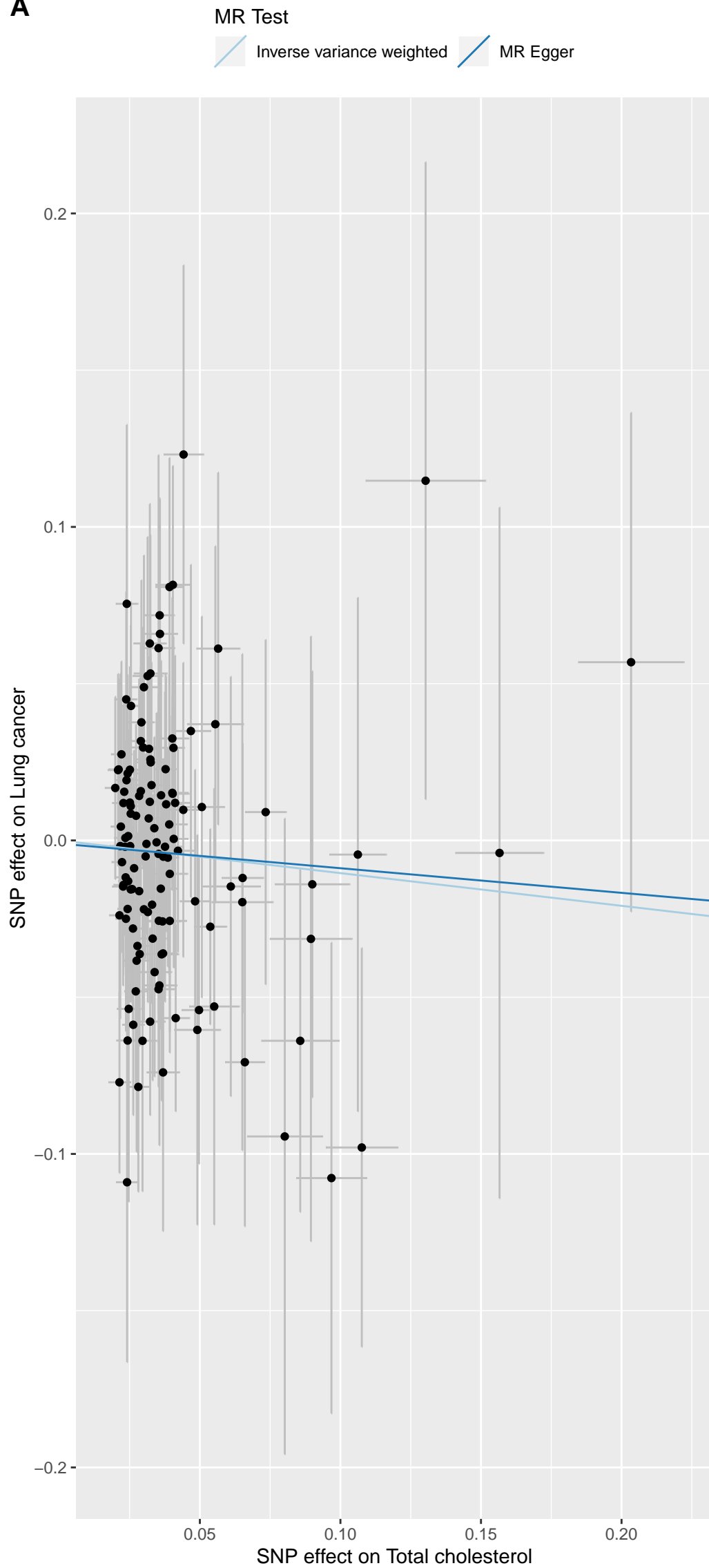**B**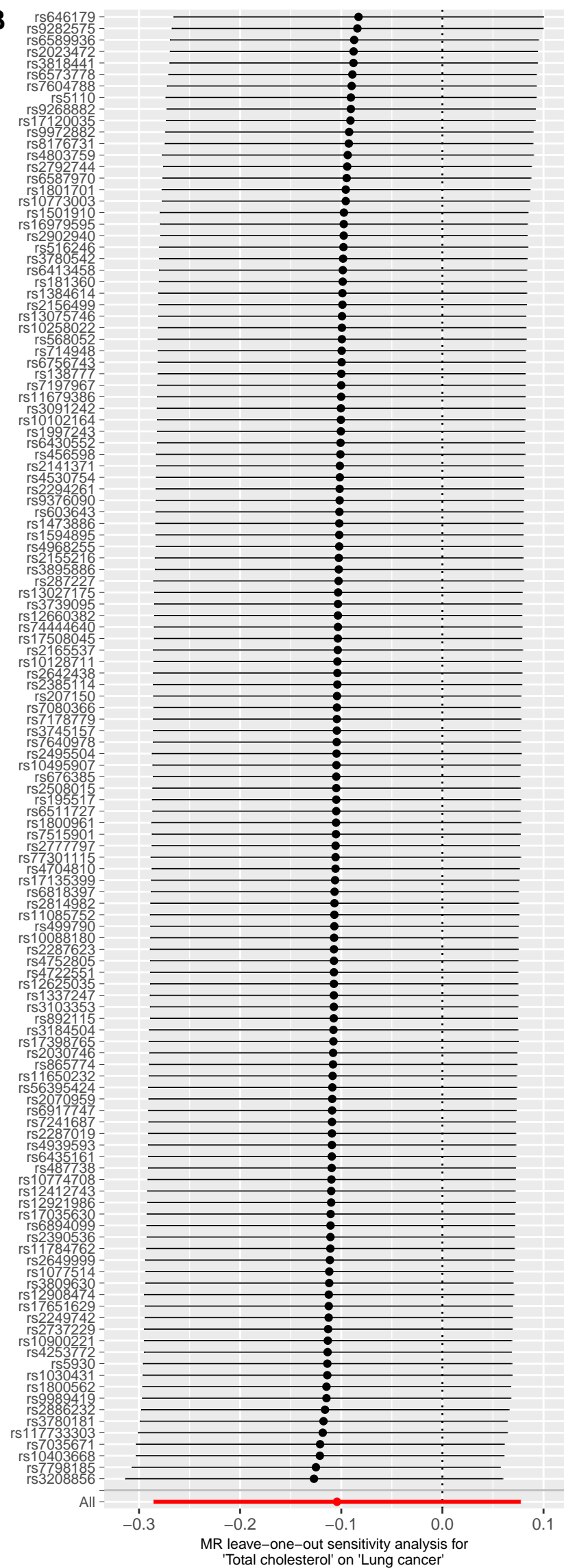

Supplement: S9 Fig — A is the scatter plot of MR result for the effect of total cholesterol on lung cancer. B is the forest plot of leave-one-out sensitivity result. (PDF) [file pone.0258498.s009.pdf]

**A**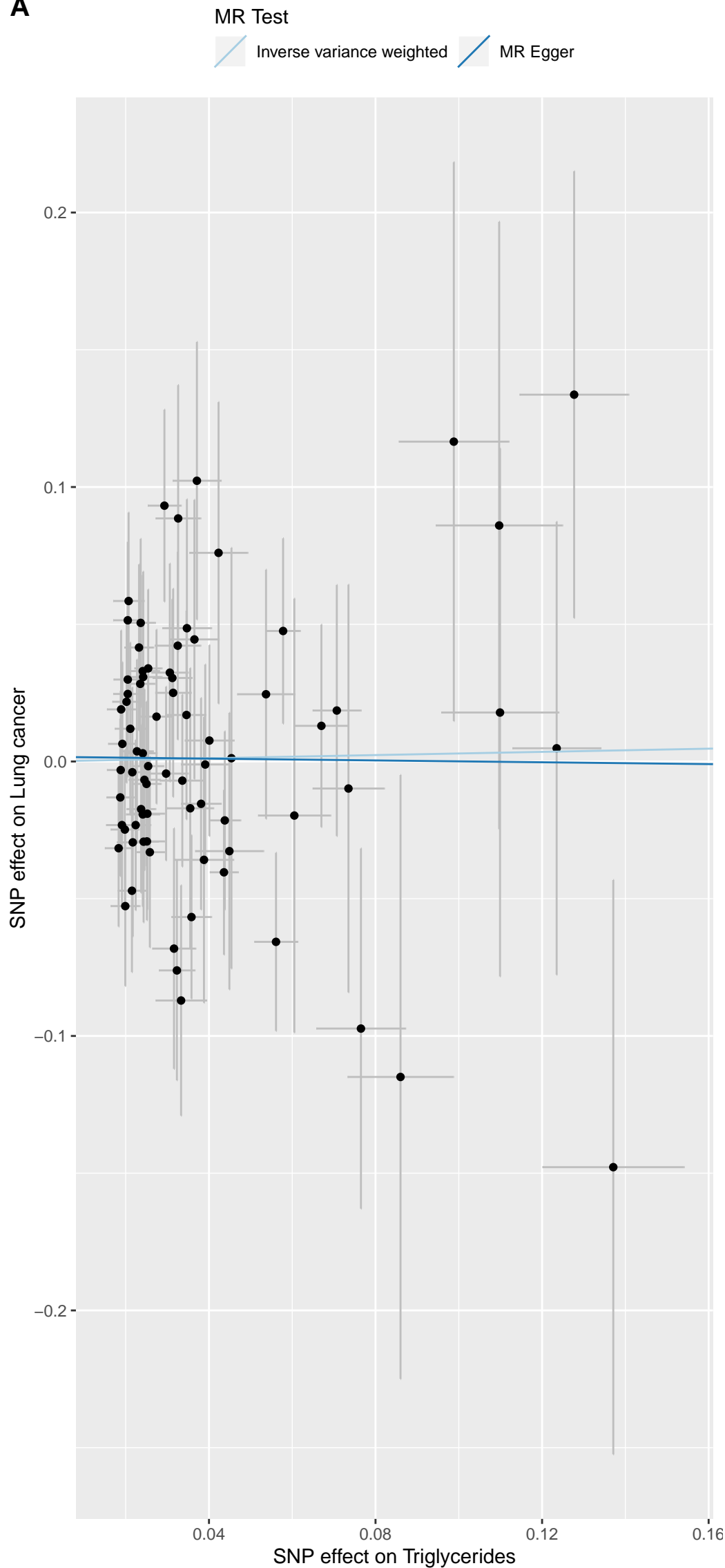**B**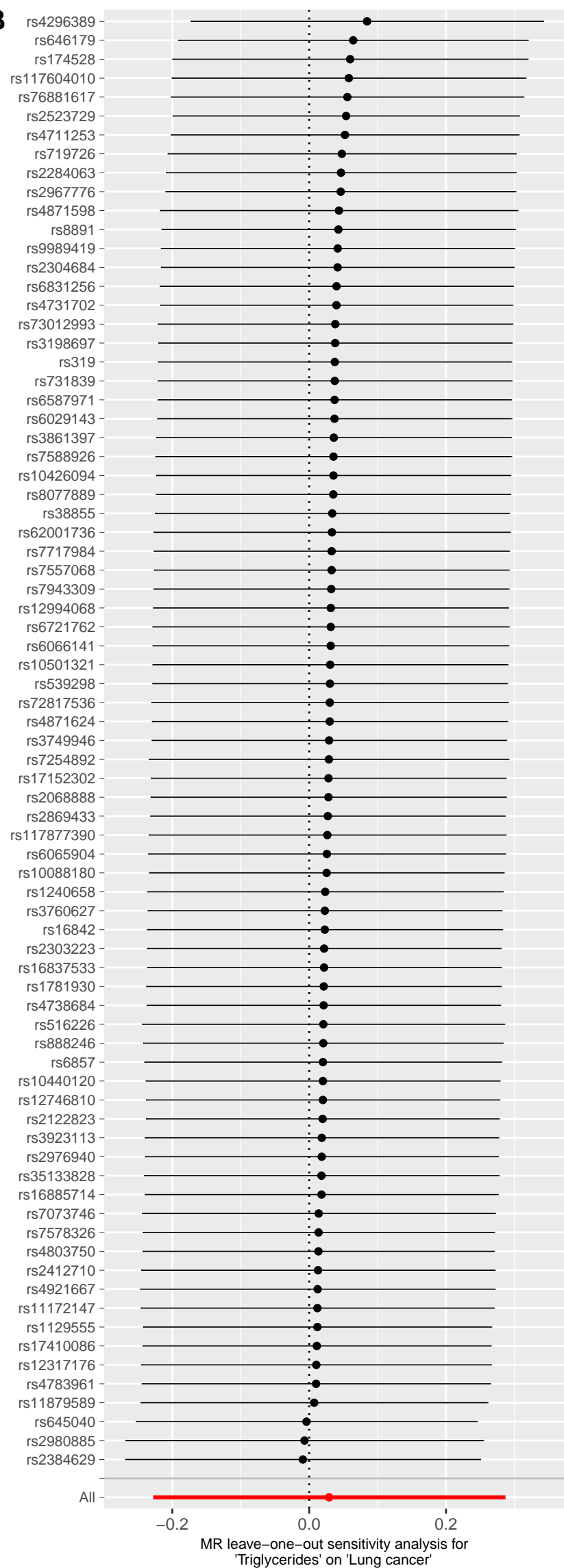

Supplement: S10 Fig — A is the scatter plot of MR result for the effect of triglycerides on lung cancer. B is the forest plot of leave-one-out sensitivity result. (PDF) [file pone.0258498.s010.pdf]

**A**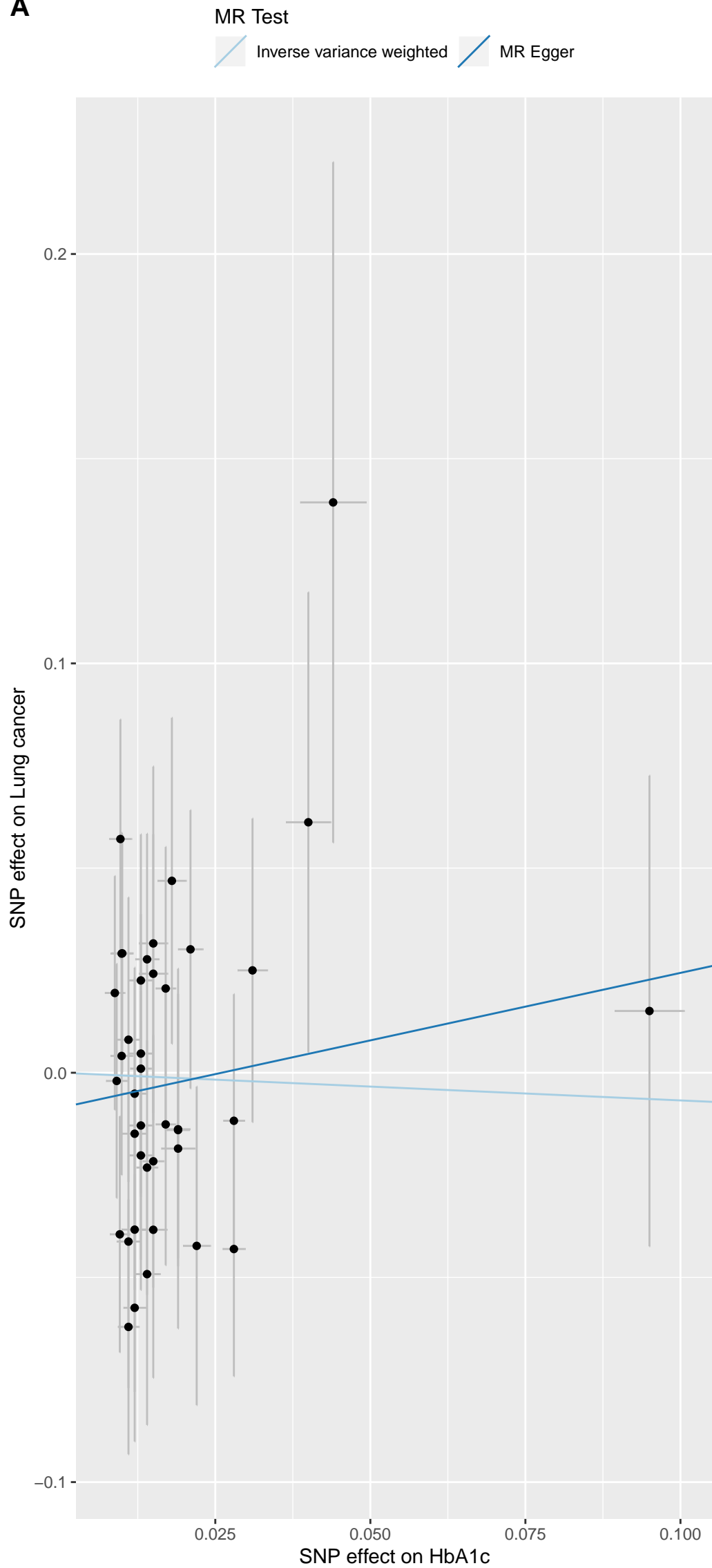**B**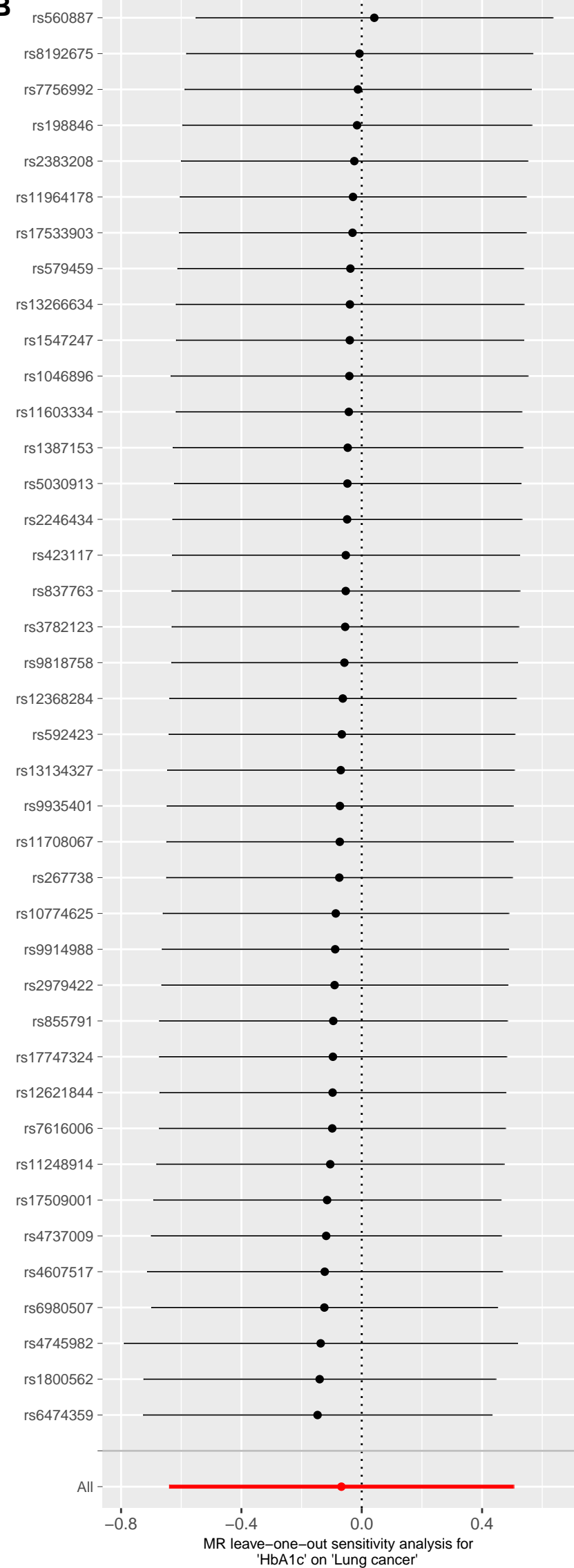

Supplement: S11 Fig — A is the scatter plot of MR result for the effect of HbA1c on lung cancer. B is the forest plot of leave-one-out sensitivity result. (PDF) [file pone.0258498.s011.pdf]

**A**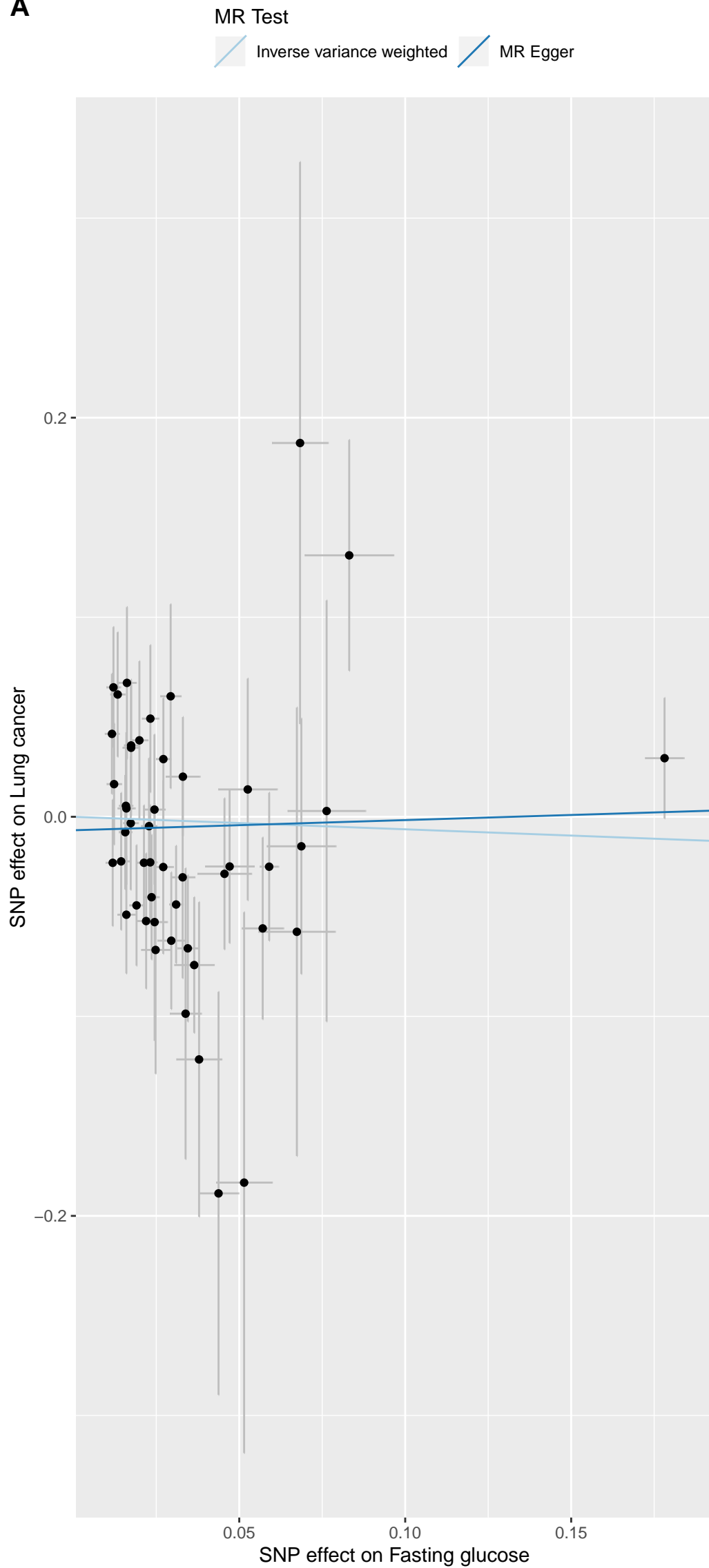**B**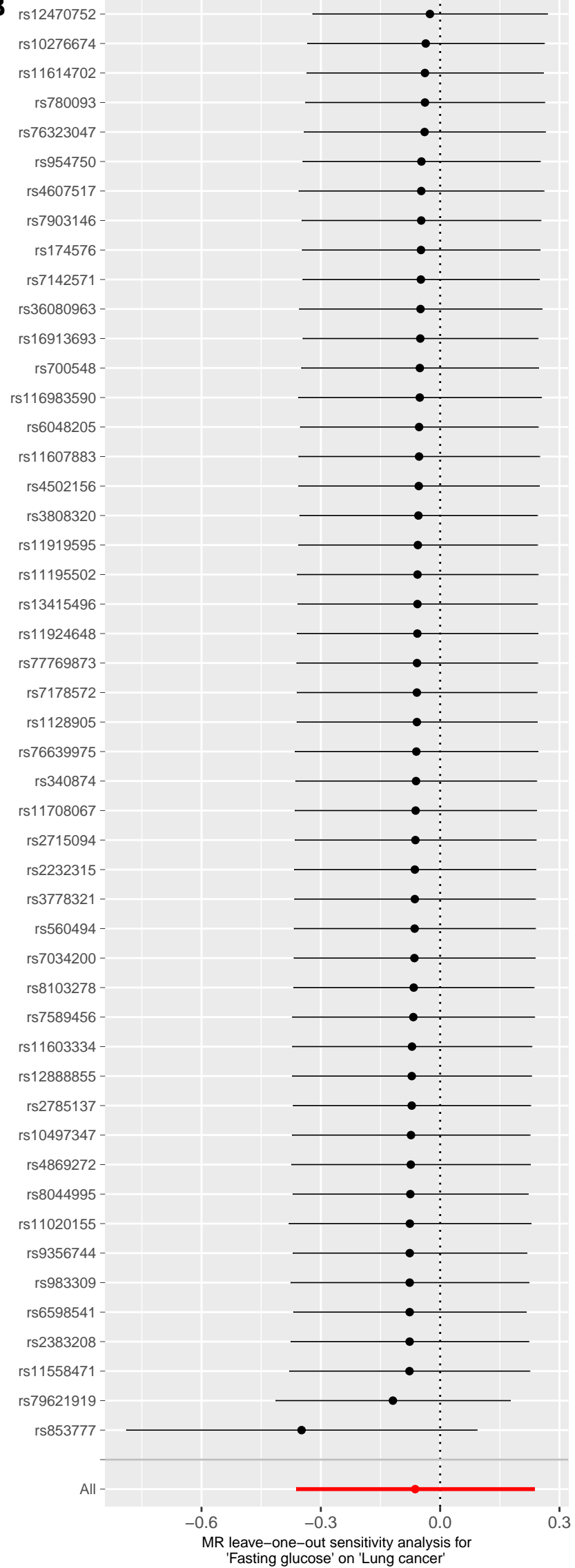

Supplement: S12 Fig — A is the scatter plot of MR result for the effect of fasting glucose on lung cancer. B is the forest plot of leave-one-out sensitivity result. (PDF) [file pone.0258498.s012.pdf]

**A**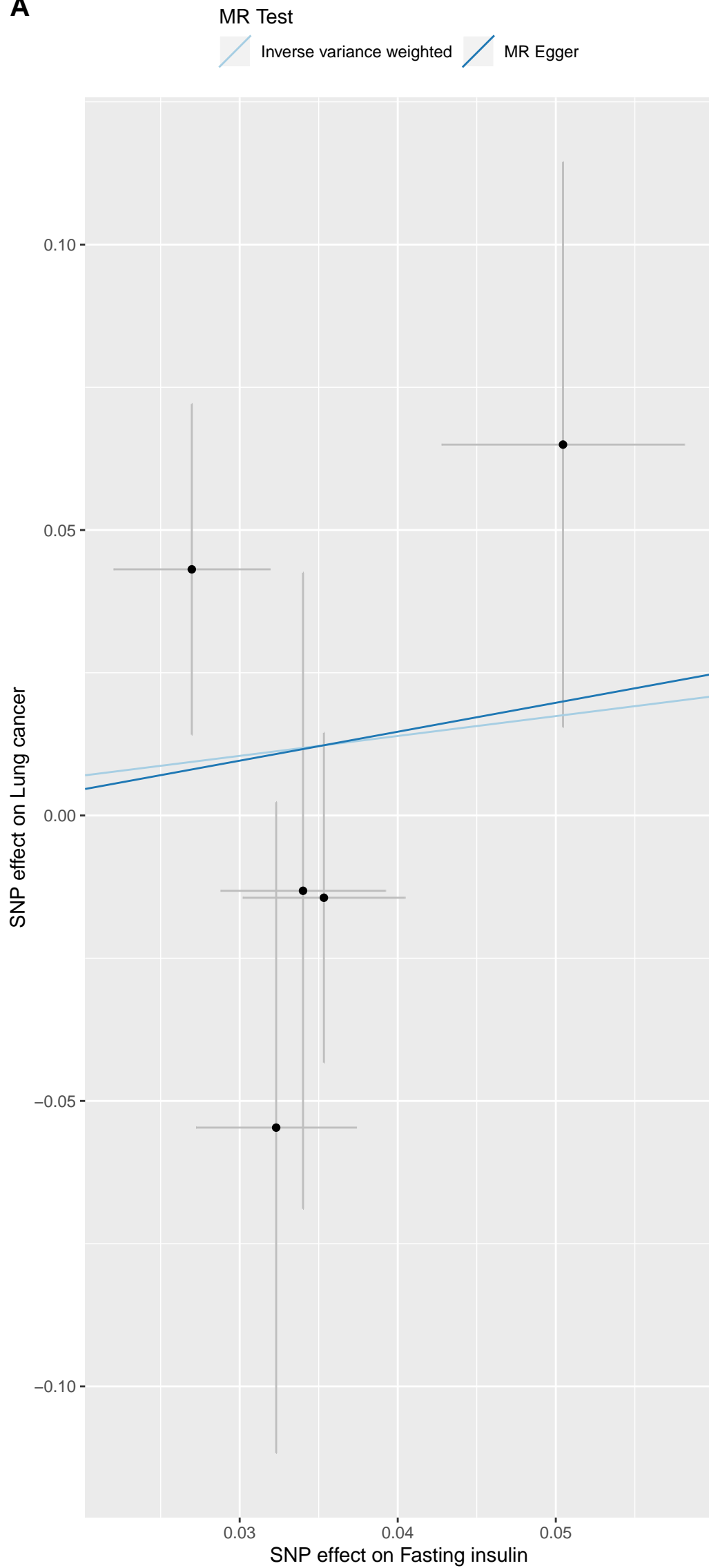**B**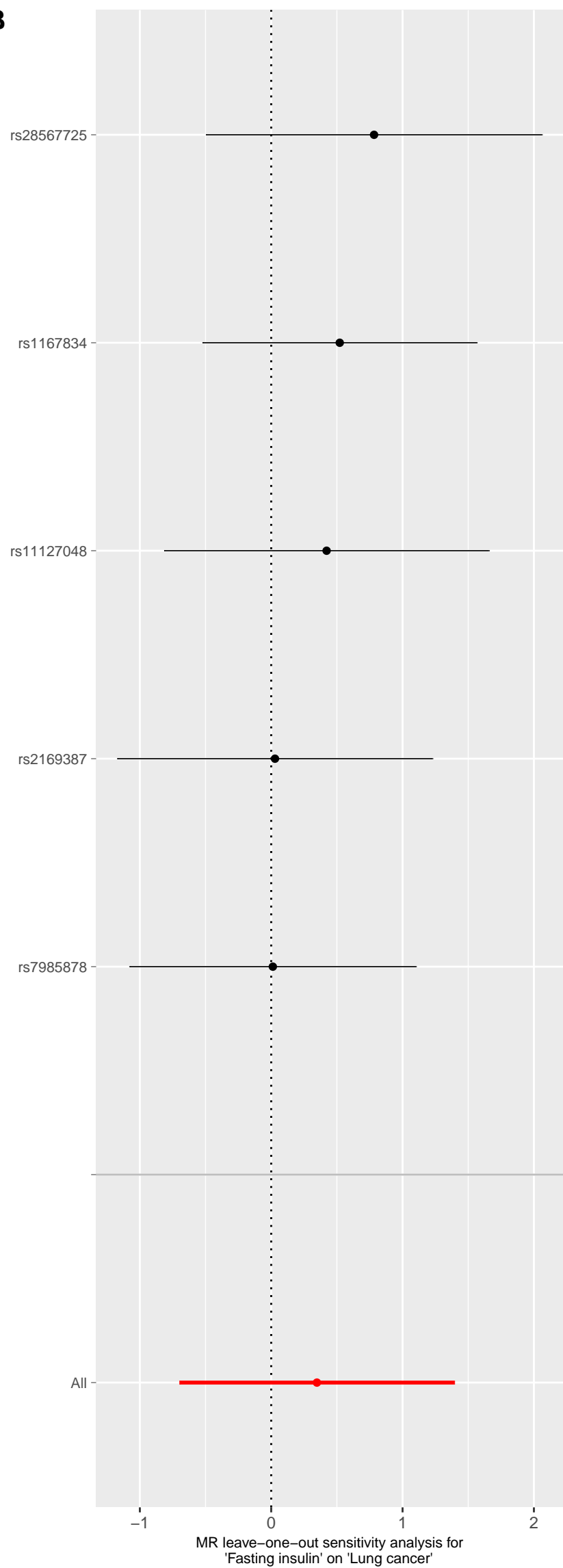

Supplement: S13 Fig — A is the scatter plot of MR result for the effect of fasting insulin on lung cancer. B is the forest plot of leave-one-out sensitivity result. (PDF) [file pone.0258498.s013.pdf]
