# Supplementary material for: Identifying modifiable risk factors of lung cancer: Indications from Mendelian randomization
Source: PLoS One. 2021 Oct 18;16(10):e0258498. doi: 10.1371/journal.pone.0258498 (PMC8523078; doi:10.1371/journal.pone.0258498)
Supplement: S2 Table — The SNP is the result of genetic variants; A1 is the effect allele; A2 is the other allele; beta is the effect size of A1 on the exposure; she is the standard error of beta; pval is the p-value of beta; F is the F statistics. (PDF) [file pone.0258498.s015.pdf]

S2 Table: Instrumental variables of smoking initiation. SNP is the rsID of genetic variants; A1 is the effect allele; A2 is the other allele; beta is the effect size of A1 on the exposure; se is the standard error of beta; pval is the p value of beta; F is the F statistics.

SNP	A1	A2	beta	se	pval	F
rs1004787	G	A	-0.034	0.004	6.90E-22	92.45
rs10211770	T	C	0.021	0.004	5.20E-09	34.11
rs10233018	A	G	-0.028	0.003	4.60E-16	65.96
rs1029984	G	T	-0.020	0.004	1.20E-08	32.49
rs10446671	T	C	0.021	0.004	3.10E-09	35.12
rs10493500	G	A	-0.026	0.004	3.70E-09	34.78
rs11012750	G	A	0.024	0.004	7.50E-11	42.38
rs11057005	G	A	-0.020	0.004	2.10E-08	31.40
rs11125335	C	T	0.024	0.004	1.10E-11	46.14
rs11165531	G	T	-0.020	0.003	4.70E-09	34.31
rs11191487	G	A	0.039	0.005	6.10E-16	65.40
rs113617088	A	G	0.030	0.005	3.30E-08	30.52
rs11594623	C	T	0.026	0.004	1.70E-10	40.78
rs11611651	A	G	0.036	0.006	1.00E-08	32.84
rs11716779	G	A	0.022	0.004	2.80E-08	30.84
rs11720703	T	C	0.020	0.004	1.20E-08	32.49
rs11768481	A	C	-0.026	0.004	3.00E-12	48.69
rs12027999	C	T	-0.030	0.005	1.30E-08	32.33
rs12186738	T	G	-0.028	0.005	1.40E-08	32.19
rs12202507	C	T	0.021	0.004	8.50E-09	33.16
rs12474587	T	G	0.025	0.004	1.10E-12	50.66
rs12530388	A	C	0.019	0.003	3.40E-08	30.46
rs12545053	G	A	0.022	0.004	4.20E-10	39.02
rs12632110	A	G	0.024	0.004	1.20E-10	41.47
rs12708665	A	G	-0.022	0.004	1.10E-08	32.66
rs1385108	T	C	0.025	0.004	2.20E-09	35.79
rs1392510	C	T	0.028	0.005	3.10E-08	30.64
rs1518393	A	C	-0.020	0.004	3.80E-08	30.25
rs17055603	A	G	-0.021	0.004	3.80E-08	30.25
rs1901477	A	G	-0.029	0.004	1.20E-15	64.07
rs1945735	G	A	-0.023	0.004	1.40E-08	32.19
rs2218439	A	G	0.024	0.003	5.80E-12	47.40
rs2237235	G	A	-0.023	0.004	5.00E-08	29.72
rs2378662	G	A	-0.020	0.003	9.10E-09	33.02
rs238896	A	G	-0.020	0.004	1.70E-08	31.81
rs2398737	G	T	-0.024	0.003	2.90E-12	48.76
rs2435211	T	C	0.022	0.004	4.40E-09	34.44
rs2585817	G	A	-0.021	0.004	5.20E-09	34.11
rs2652430	A	G	-0.022	0.004	4.10E-10	39.06

rs2710331	C	T	0.022	0.004	3.00E-09	35.18
rs281287	G	A	0.028	0.004	3.80E-14	57.27
rs28717373	T	C	-0.026	0.004	3.00E-13	53.21
rs3001723	A	G	0.032	0.004	1.60E-16	68.04
rs301805	T	G	-0.021	0.004	2.80E-09	35.32
rs34121288	T	C	0.020	0.004	4.90E-08	29.76
rs34626694	T	C	0.021	0.004	2.50E-08	31.06
rs36096261	A	G	0.031	0.005	6.30E-09	33.74
rs3741499	T	C	-0.021	0.004	2.00E-08	31.49
rs3843409	T	C	0.020	0.004	2.10E-08	31.40
rs4044321	A	G	0.029	0.004	2.20E-15	62.88
rs4255357	G	A	-0.023	0.004	5.90E-11	42.85
rs4523689	G	A	-0.021	0.004	8.90E-09	33.07
rs4543592	C	T	0.019	0.003	2.40E-08	31.14
rs455650	C	T	0.039	0.004	4.10E-18	75.27
rs4571506	T	C	-0.023	0.004	8.80E-11	42.07
rs6119893	T	G	0.024	0.004	1.20E-10	41.47
rs61959481	A	G	-0.024	0.004	3.10E-08	30.64
rs62025923	C	T	-0.028	0.004	6.00E-11	42.82
rs6265	T	C	-0.034	0.004	1.30E-14	59.38
rs6438208	A	G	-0.025	0.004	2.90E-10	39.74
rs6499255	G	A	0.027	0.005	4.70E-09	34.31
rs6728726	T	C	-0.033	0.005	6.00E-13	51.85
rs6756212	C	T	0.039	0.003	2.90E-29	126.12
rs6948707	G	T	0.024	0.004	2.10E-11	44.88
rs7005565	C	T	0.024	0.004	1.50E-09	36.53
rs7083526	C	T	-0.025	0.003	9.10E-13	51.03
rs7110863	G	A	0.044	0.004	1.60E-35	154.73
rs7197072	T	C	-0.024	0.004	7.10E-09	33.51
rs748832	G	A	0.021	0.004	1.20E-08	32.49
rs7505855	C	T	0.020	0.003	8.90E-09	33.07
rs7553158	G	A	0.021	0.004	5.40E-09	34.04
rs77464064	T	C	0.041	0.007	5.60E-09	33.97
rs7787612	T	C	-0.023	0.004	6.60E-09	33.65
rs7829715	T	C	0.025	0.003	5.70E-13	51.95
rs7901883	A	G	-0.026	0.004	2.10E-10	40.37
rs7969559	A	G	0.025	0.004	1.60E-10	40.90
rs7993498	T	C	-0.022	0.004	4.70E-10	38.80
rs9375328	C	T	0.027	0.004	1.30E-11	45.81
rs9517908	G	A	0.025	0.004	4.30E-09	34.48
rs9540731	C	T	0.021	0.004	4.00E-09	34.62
rs9922607	T	C	-0.024	0.004	4.60E-08	29.88
rs993700	T	C	0.026	0.004	4.20E-10	39.02
