# Supplementary material for: Identifying modifiable risk factors of lung cancer: Indications from Mendelian randomization
Source: PLoS One. 2021 Oct 18;16(10):e0258498. doi: 10.1371/journal.pone.0258498 (PMC8523078; doi:10.1371/journal.pone.0258498)
Supplement: S3 Table — The SNP is the result of genetic variants; A1 is the effect allele; A2 is the other allele; beta is the effect size of A1 on the exposure; she is the standard error of beta; pval is the p-value of beta; F is the F statistics. (PDF) [file pone.0258498.s016.pdf]

S3 Table: Instrumental variables of smoking cessation. SNP is the rsID of genetic variants; A1 is the effect allele; A2 is the other allele; beta is the effect size of A1 on the exposure; se is the standard error of beta; pval is the p value of beta; F is the F statistics.

SNP	A1	A2	beta	se	pval	F
rs11090045	G	A	0.029	0.005	9.90E-09	32.86
rs113382419	C	A	-0.081	0.007	1.00E-27	119.09
rs12891477	C	T	-0.029	0.005	1.60E-09	36.41
rs215599	T	C	-0.026	0.005	3.60E-08	30.35
rs329120	C	T	0.026	0.005	3.40E-08	30.46
rs4955411	G	A	-0.036	0.006	1.30E-10	41.31
rs6011779	T	C	-0.056	0.006	2.40E-21	89.98
rs61902812	C	A	0.055	0.010	1.90E-08	31.59
rs62262671	A	G	-0.037	0.007	4.10E-08	30.10
rs637473	T	C	0.030	0.005	3.60E-09	34.83
rs7932056	G	A	-0.032	0.005	1.00E-09	37.32
