# Supplementary material for: Identifying modifiable risk factors of lung cancer: Indications from Mendelian randomization
Source: PLoS One. 2021 Oct 18;16(10):e0258498. doi: 10.1371/journal.pone.0258498 (PMC8523078; doi:10.1371/journal.pone.0258498)
Supplement: S4 Table — The SNP is the result of genetic variants; A1 is the effect allele; A2 is the other allele; beta is the effect size of A1 on the exposure; she is the standard error of beta; pval is the p-value of beta; F is the F statistics. (PDF) [file pone.0258498.s017.pdf]

S4 Table: Instrumental variables of cigarettes per day. SNP is the rsID of genetic variants; A1 is the effect allele; A2 is the other allele; beta is the effect size of A1 on the exposure; se is the standard error of beta; pval is the p value of beta; F is the F statistics.

SNP	A1	A2	beta	se	pval	F
rs10092346	C	T	-0.029	0.005	6.86E-10	38.06
rs10419393	G	A	0.034	0.006	1.96E-08	31.54
rs1051730	A	G	0.095	0.004	5.51E-121	547.05
rs1052035	C	T	0.066	0.009	2.06E-13	53.95
rs11072806	A	G	0.050	0.004	9.11E-38	165.01
rs111280114	G	A	0.038	0.007	7.87E-09	33.31
rs111684981	G	A	-0.128	0.019	2.61E-11	44.45
rs112889062	T	C	-0.131	0.020	1.97E-11	45.00
rs113864525	A	C	0.039	0.006	4.18E-11	43.53
rs11638490	T	C	0.058	0.004	3.20E-50	222.07
rs11670760	C	T	0.051	0.007	2.17E-14	58.37
rs117189732	T	C	0.042	0.006	1.25E-10	41.38
rs117248593	T	C	-0.130	0.020	3.67E-11	43.78
rs117287096	A	G	-0.154	0.022	1.83E-12	49.66
rs117422348	T	C	-0.147	0.021	8.14E-13	51.25
rs117540499	A	G	-0.160	0.021	7.49E-14	55.94
rs117947334	T	C	-0.129	0.019	1.69E-11	45.31
rs11858836	A	G	0.084	0.004	9.04E-96	431.17
rs12593207	A	G	-0.051	0.007	3.60E-14	57.38
rs12904234	T	C	0.055	0.005	2.46E-26	112.74
rs12905641	T	C	-0.028	0.004	3.51E-12	48.38
rs12914385	T	C	0.086	0.004	3.44E-105	474.45
rs1316971	G	A	0.072	0.005	1.36E-48	214.60
rs1317286	G	A	0.094	0.004	4.39E-118	533.72
rs13180	T	C	0.058	0.004	5.65E-48	211.77
rs1394371	T	C	0.074	0.004	1.64E-67	301.39
rs1451240	G	A	0.034	0.005	6.47E-14	56.22
rs16969968	A	G	0.096	0.003	7.58E-185	840.72
rs17486278	C	A	0.094	0.004	8.32E-117	527.84
rs1994016	T	C	0.045	0.003	2.25E-43	190.69
rs1996371	C	T	0.059	0.004	5.22E-51	225.68
rs3025343	A	G	0.038	0.005	1.13E-14	59.66
rs34406232	A	C	-0.121	0.019	1.51E-10	41.01
rs3825807	G	A	0.048	0.003	6.26E-48	211.56
rs3885951	G	A	0.066	0.007	8.35E-23	96.63
rs4380028	T	C	0.030	0.004	6.55E-13	51.68
rs4398905	T	C	-0.034	0.006	6.78E-09	33.60
rs4436747	G	A	-0.026	0.004	9.30E-12	46.47
rs4803365	A	G	-0.130	0.020	3.47E-11	43.89

rs4803367	G	T	-0.129	0.020	5.58E-11	42.96
rs4803400	G	T	0.033	0.006	2.31E-08	31.21
rs55853698	G	T	0.095	0.004	4.40E-121	547.50
rs56057809	T	C	0.039	0.006	7.97E-11	42.27
rs569207	T	C	-0.080	0.005	2.18E-65	291.64
rs578776	A	G	-0.072	0.004	5.28E-64	285.29
rs6474412	T	C	0.035	0.004	2.15E-21	90.21
rs6495307	T	C	-0.028	0.004	1.11E-12	50.64
rs6495308	C	T	-0.081	0.005	9.82E-69	307.00
rs6495309	T	C	-0.083	0.005	1.00E-59	265.66
rs667282	C	T	-0.079	0.005	6.74E-65	289.39
rs680244	C	T	0.031	0.004	8.87E-16	64.67
rs7247903	G	A	-0.074	0.008	1.62E-19	81.66
rs7251418	G	A	0.051	0.007	1.87E-14	58.67
rs72740964	A	G	0.094	0.004	2.67E-117	530.11
rs73034462	A	G	0.037	0.006	2.09E-09	35.89
rs739447	T	C	0.039	0.006	4.47E-11	43.40
rs75106522	A	G	0.054	0.009	3.42E-09	34.93
rs79016062	T	G	-0.132	0.020	1.86E-11	45.12
rs7937	T	C	0.042	0.006	1.32E-12	50.30
rs80208490	G	A	-0.130	0.020	3.18E-11	44.06
rs8034191	C	T	0.095	0.003	8.62E-183	831.26
rs8040868	C	T	0.085	0.004	7.96E-103	463.59
rs8042374	G	A	-0.082	0.006	1.21E-39	173.60
rs8043119	A	G	0.054	0.004	2.23E-43	190.71
rs8053	C	T	0.034	0.004	3.16E-18	75.78
rs938682	A	G	0.082	0.004	2.24E-107	484.50
rs951266	A	G	0.094	0.004	1.58E-117	531.16
