# Supplementary material for: Identifying modifiable risk factors of lung cancer: Indications from Mendelian randomization
Source: PLoS One. 2021 Oct 18;16(10):e0258498. doi: 10.1371/journal.pone.0258498 (PMC8523078; doi:10.1371/journal.pone.0258498)
Supplement: S5 Table — The SNP is the result of genetic variants; A1 is the effect allele; A2 is the other allele; beta is the effect size of A1 on the exposure; she is the standard error of beta; pval is the p-value of beta; F is the F statistics. (PDF) [file pone.0258498.s018.pdf]

S5 Table: Instrumental variables of BMI. SNP is the rsID of genetic variants; A1 is the effect allele; A2 is the other allele; beta is the effect size of A1 on the exposure; se is the standard error of beta; pval is the p value of beta; F is the F statistics.

SNP	A1	A2	beta	se	pval	F
rs1000940	G	A	0.019	0.003	1.28E-08	40.11
rs10132280	C	A	0.023	0.003	1.14E-11	58.78
rs1016287	T	C	0.023	0.003	2.25E-11	58.78
rs10182181	G	A	0.031	0.003	8.78E-24	106.78
rs10938397	G	A	0.040	0.003	3.21E-38	177.78
rs10968576	G	A	0.025	0.003	6.61E-14	69.44
rs11030104	A	G	0.041	0.004	5.56E-28	105.06
rs11057405	G	A	0.031	0.006	2.02E-08	26.69
rs11126666	A	G	0.021	0.003	1.33E-09	49.00
rs11165643	T	C	0.022	0.003	2.07E-12	53.78
rs11191560	C	T	0.031	0.005	8.45E-09	38.44
rs11583200	C	T	0.018	0.003	1.48E-08	36.00
rs1167827	G	A	0.020	0.003	6.33E-10	44.44
rs11688816	G	A	0.017	0.003	1.89E-08	32.11
rs11727676	T	C	0.036	0.006	2.55E-08	36.00
rs11847697	T	C	0.049	0.008	3.99E-09	37.52
rs12286929	G	A	0.022	0.003	1.31E-12	53.78
rs12401738	A	G	0.021	0.003	1.15E-10	49.00
rs12429545	A	G	0.033	0.005	1.09E-12	43.56
rs12446632	G	A	0.040	0.005	1.48E-18	64.00
rs12566985	G	A	0.024	0.003	3.28E-15	64.00
rs12885454	C	A	0.021	0.003	1.94E-10	49.00
rs12940622	G	A	0.018	0.003	2.49E-09	36.00
rs13021737	G	A	0.060	0.004	1.11E-50	225.00
rs13078960	G	T	0.030	0.004	1.74E-14	56.25
rs13107325	T	C	0.048	0.007	1.83E-12	47.02
rs13191362	A	G	0.028	0.005	7.34E-09	31.36
rs13201877	G	A	0.023	0.005	4.29E-08	21.16
rs1441264	A	G	0.018	0.003	2.96E-08	36.00
rs1460676	C	T	0.020	0.004	4.98E-08	25.00
rs1516725	C	T	0.045	0.005	1.89E-22	81.00
rs1528435	T	C	0.018	0.003	1.20E-08	36.00
rs16851483	T	G	0.048	0.008	3.55E-10	36.00
rs16907751	C	T	0.035	0.007	3.89E-08	25.00
rs16951275	T	C	0.031	0.004	1.91E-17	60.06
rs17024393	C	T	0.066	0.009	7.03E-14	53.78
rs17094222	C	T	0.025	0.004	5.94E-11	39.06
rs17203016	G	A	0.021	0.004	3.41E-08	27.56
rs17405819	T	C	0.022	0.003	2.07E-11	53.78

rs17724992	A	G	0.019	0.004	3.42E-08	22.56
rs1808579	C	T	0.017	0.003	4.17E-08	32.11
rs1928295	T	C	0.019	0.003	7.91E-10	40.11
rs2033529	G	A	0.019	0.003	1.39E-08	40.11
rs2033732	C	T	0.019	0.004	4.89E-08	22.56
rs205262	G	A	0.022	0.004	1.75E-10	30.25
rs2075650	A	G	0.026	0.005	1.25E-08	27.04
rs2080454	C	A	0.017	0.003	8.60E-09	32.11
rs2112347	T	G	0.026	0.003	6.19E-17	75.11
rs2121279	T	C	0.025	0.004	2.31E-08	39.06
rs2176040	A	G	0.014	0.003	9.99E-09	21.78
rs2176598	T	C	0.020	0.004	2.97E-08	25.00
rs2207139	G	A	0.045	0.004	4.13E-29	126.56
rs2245368	C	T	0.032	0.006	3.19E-08	28.44
rs2287019	C	T	0.036	0.004	4.59E-18	81.00
rs2365389	C	T	0.020	0.003	1.63E-10	44.44
rs2650492	A	G	0.021	0.004	1.92E-09	27.56
rs2820292	C	A	0.020	0.003	1.83E-10	44.44
rs2836754	C	T	0.016	0.003	1.61E-08	28.44
rs29941	G	A	0.018	0.003	2.41E-08	36.00
rs3101336	C	T	0.033	0.003	2.66E-26	121.00
rs3736485	A	G	0.018	0.003	7.41E-09	36.00
rs3810291	A	G	0.028	0.004	4.81E-15	49.00
rs3817334	T	C	0.026	0.003	5.15E-17	75.11
rs3849570	A	C	0.019	0.003	2.60E-08	40.11
rs3888190	A	C	0.031	0.003	3.14E-23	106.78
rs4740619	T	C	0.018	0.003	4.56E-09	36.00
rs4787491	G	A	0.016	0.003	2.70E-08	28.44
rs492400	C	T	0.016	0.003	6.78E-09	28.44
rs543874	G	A	0.048	0.004	2.62E-35	144.00
rs6091540	C	T	0.019	0.004	2.15E-11	22.56
rs6465468	T	G	0.017	0.004	4.98E-08	18.06
rs6477694	C	T	0.017	0.003	2.67E-08	32.11
rs6567160	C	T	0.056	0.004	3.93E-53	196.00
rs657452	A	G	0.023	0.003	5.48E-13	58.78
rs6804842	G	A	0.019	0.003	2.48E-09	40.11
rs7138803	A	G	0.032	0.003	8.15E-24	113.78
rs7141420	T	C	0.024	0.003	1.23E-14	64.00
rs7164727	T	C	0.018	0.003	3.92E-09	36.00
rs7239883	G	A	0.016	0.003	1.51E-08	28.44
rs7243357	T	G	0.022	0.004	3.86E-08	30.25
rs758747	T	C	0.023	0.004	7.47E-10	33.06
rs7599312	G	A	0.022	0.003	1.17E-10	53.78
rs7715256	G	T	0.016	0.003	8.85E-09	28.44

rs7899106	G	A	0.040	0.007	2.96E-08	32.65
rs7903146	C	T	0.023	0.003	1.11E-11	58.78
rs9374842	T	C	0.019	0.004	2.67E-08	22.56
rs9400239	C	T	0.019	0.003	1.61E-08	40.11
rs9540493	A	G	0.017	0.003	4.97E-08	32.11
rs977747	T	G	0.017	0.003	2.18E-08	32.11
rs9925964	A	G	0.019	0.003	8.11E-10	40.11
