# Supplementary material for: Identifying modifiable risk factors of lung cancer: Indications from Mendelian randomization
Source: PLoS One. 2021 Oct 18;16(10):e0258498. doi: 10.1371/journal.pone.0258498 (PMC8523078; doi:10.1371/journal.pone.0258498)
Supplement: S6 Table — The SNP is the result of genetic variants; A1 is the effect allele; A2 is the other allele; beta is the effect size of A1 on the exposure; she is the standard error of beta; pval is the p-value of beta; F is the F statistics. (PDF) [file pone.0258498.s019.pdf]

S6 Table: Instrumental variables of hip circumference. SNP is the rsID of genetic variants; A1 is the effect allele; A2 is the other allele; beta is the effect size of A1 on the exposure; se is the standard error of beta; pval is the p value of beta; F is the F statistics.

SNP	A1	A2	beta	se	pval	F
rs10132280	A	C	-0.022	0.004	4.80E-09	33.52
rs10929925	C	A	0.019	0.004	4.50E-08	27.85
rs10938397	A	G	-0.030	0.004	9.30E-17	65.74
rs11057405	A	G	-0.040	0.006	2.10E-10	40.31
rs11165623	A	G	0.022	0.004	3.00E-10	39.51
rs11672660	C	T	0.028	0.005	7.10E-10	38.72
rs11676272	G	A	0.026	0.004	3.80E-11	44.44
rs12086130	T	C	0.038	0.006	2.60E-09	36.38
rs12446632	A	G	-0.036	0.005	1.10E-11	47.93
rs1294409	T	C	-0.020	0.004	3.00E-08	29.22
rs13098327	G	A	-0.027	0.004	1.20E-09	37.65
rs1351394	T	C	0.023	0.004	6.40E-11	43.18
rs13695	C	T	-0.024	0.004	4.30E-08	29.75
rs143384	A	G	-0.026	0.004	3.90E-12	46.81
rs1516725	T	C	-0.031	0.005	1.00E-08	32.96
rs1548457	T	C	-0.025	0.005	1.70E-08	30.86
rs16894959	C	T	0.037	0.005	9.60E-14	57.02
rs16905212	C	T	-0.021	0.004	1.90E-08	30.54
rs17024393	C	T	0.063	0.010	2.80E-10	39.69
rs17066842	G	A	0.059	0.010	1.70E-09	37.00
rs17391694	T	C	0.039	0.006	3.00E-10	40.88
rs1808579	T	C	-0.023	0.004	2.10E-10	43.18
rs2112347	G	T	-0.025	0.004	9.70E-12	48.23
rs2206277	T	C	0.039	0.005	3.10E-17	71.88
rs2293576	A	G	-0.023	0.004	2.10E-09	36.63
rs2301573	T	C	-0.035	0.006	2.10E-08	31.87
rs2820443	C	T	0.034	0.004	4.90E-18	76.00
rs3087591	A	G	-0.021	0.004	2.50E-08	30.54
rs355838	T	G	-0.022	0.004	1.60E-09	37.35
rs3800229	T	G	0.021	0.004	3.20E-08	30.54
rs3810291	A	G	0.023	0.004	2.40E-08	31.47
rs3888190	A	C	0.035	0.004	9.40E-22	94.52
rs4132228	C	T	-0.021	0.004	3.10E-08	28.99
rs4883723	G	A	-0.032	0.005	5.20E-10	39.37
rs4889606	G	A	-0.021	0.004	8.80E-09	34.03
rs543874	G	A	0.045	0.005	1.80E-23	100.00
rs6163	C	A	-0.021	0.004	1.60E-08	32.21
rs6265	C	T	0.034	0.005	1.50E-14	57.09
rs6569648	T	C	-0.029	0.004	6.50E-12	47.68

rs663129	A	G	0.050	0.004	1.40E-32	141.72
rs6755502	T	C	-0.054	0.005	2.40E-30	132.01
rs7138803	G	A	-0.029	0.004	1.90E-15	61.43
rs7144011	T	G	0.030	0.004	8.60E-13	51.02
rs7183263	G	T	0.019	0.004	4.80E-08	27.85
rs7531118	T	C	-0.024	0.004	4.00E-11	42.07
rs7632381	T	C	-0.036	0.004	5.30E-24	105.80
rs7903146	T	C	-0.026	0.004	2.10E-11	44.44
rs798528	A	C	0.021	0.004	1.70E-08	30.54
rs806794	G	A	-0.032	0.004	2.80E-16	64.00
rs879620	C	T	-0.029	0.005	8.90E-10	38.07
rs887912	T	C	0.022	0.004	1.60E-08	31.82
rs951252	G	A	-0.028	0.004	4.50E-15	64.00
rs9939973	A	G	0.070	0.004	2.30E-86	378.09
