# Supplementary material for: Identifying modifiable risk factors of lung cancer: Indications from Mendelian randomization
Source: PLoS One. 2021 Oct 18;16(10):e0258498. doi: 10.1371/journal.pone.0258498 (PMC8523078; doi:10.1371/journal.pone.0258498)
Supplement: S7 Table — The SNP is the result of genetic variants; A1 is the effect allele; A2 is the other allele; beta is the effect size of A1 on the exposure; she is the standard error of beta; pval is the p-value of beta; F is the F statistics. (PDF) [file pone.0258498.s020.pdf]

S7 Table: Instrumental variables of waist circumference. SNP is the rsID of genetic variants; A1 is the effect allele; A2 is the other allele; beta is the effect size of A1 on the exposure; se is the standard error of beta; pval is the p value of beta; F is the F statistics.

SNP	A1	A2	beta	se	pval	F
rs10132280	A	C	-0.022	0.004	2.20E-09	35.35
rs10840100	G	A	0.020	0.004	5.40E-09	32.65
rs10938397	A	G	-0.032	0.004	6.10E-20	83.59
rs10968576	G	A	0.025	0.004	1.20E-11	48.23
rs11165623	A	G	0.020	0.003	5.20E-09	34.60
rs11873305	A	C	0.058	0.009	3.10E-10	39.74
rs12429545	G	A	-0.031	0.005	2.50E-09	35.54
rs12446632	A	G	-0.036	0.005	5.20E-13	51.84
rs12885454	C	A	0.020	0.004	2.60E-08	32.65
rs1516725	T	C	-0.031	0.005	1.70E-09	36.95
rs1549293	T	C	-0.020	0.004	7.30E-09	32.65
rs16894959	C	T	0.026	0.005	3.40E-08	29.34
rs16996700	T	C	0.023	0.004	1.50E-09	38.64
rs2075650	A	G	0.031	0.005	8.90E-10	38.44
rs2112347	G	T	-0.025	0.004	3.20E-13	51.02
rs2287019	C	T	0.035	0.005	1.70E-14	57.89
rs2293576	A	G	-0.022	0.004	9.40E-10	37.35
rs2325036	A	C	0.023	0.004	2.10E-11	43.18
rs2489623	C	A	0.019	0.003	3.40E-08	31.23
rs2531992	A	G	-0.028	0.005	3.00E-09	34.03
rs2820292	A	C	-0.019	0.003	2.40E-08	31.23
rs3127553	G	A	0.023	0.004	1.60E-10	43.18
rs3810291	A	G	0.026	0.004	1.70E-10	42.25
rs3849570	A	C	0.021	0.004	2.20E-08	30.54
rs4130548	C	T	0.022	0.004	3.40E-10	39.51
rs6163	C	A	-0.019	0.004	3.70E-08	29.47
rs633715	C	T	0.043	0.004	3.30E-23	100.00
rs6440003	G	A	-0.021	0.003	2.90E-10	38.15
rs6545714	G	A	0.022	0.004	1.90E-10	39.51
rs6567160	C	T	0.048	0.004	2.60E-33	144.00
rs6755502	T	C	-0.051	0.005	2.00E-30	128.44
rs7138803	G	A	-0.028	0.004	1.60E-15	64.00
rs7144011	T	G	0.033	0.004	9.40E-16	64.78
rs7239883	G	A	0.021	0.004	2.30E-09	36.00
rs7498665	G	A	0.034	0.004	1.40E-22	94.37
rs7531118	T	C	-0.027	0.004	1.50E-14	59.51
rs7550711	T	C	0.058	0.010	3.40E-09	35.03
rs7903146	T	C	-0.022	0.004	3.90E-09	35.35
rs806794	G	A	-0.022	0.004	2.10E-09	35.35

rs929641	A	G	0.021	0.003	1.20E-09	38.15
rs9400239	C	T	0.024	0.004	1.90E-11	44.44
rs943005	T	C	0.039	0.004	7.20E-19	78.56
