# Supplementary material for: Identifying modifiable risk factors of lung cancer: Indications from Mendelian randomization
Source: PLoS One. 2021 Oct 18;16(10):e0258498. doi: 10.1371/journal.pone.0258498 (PMC8523078; doi:10.1371/journal.pone.0258498)
Supplement: S8 Table — The SNP is the result of genetic variants; A1 is the effect allele; A2 is the other allele; beta is the effect size of A1 on the exposure; she is the standard error of beta; pval is the p-value of beta; F is the F statistics. (PDF) [file pone.0258498.s021.pdf]

S8 Table: Instrumental variables of WHR. SNP is the rsID of genetic variants; A1 is the effect allele; A2 is the other allele; beta is the effect size of A1 on the exposure; se is the standard error of beta; pval is the p value of beta; F is the F statistics.

SNP	A1	A2	beta	se	pval	F
rs1011731	G	A	0.019	0.003	1.10E-08	33.15
rs10245353	A	C	0.027	0.004	1.60E-10	41.33
rs10783615	G	A	0.035	0.005	7.00E-13	51.02
rs10876528	A	C	0.028	0.004	1.10E-12	51.55
rs11048470	T	G	0.025	0.004	6.30E-12	45.65
rs1121980	A	G	0.043	0.003	1.30E-38	169.79
rs1128249	G	T	0.021	0.003	1.60E-09	38.15
rs11663816	C	T	0.025	0.004	2.70E-11	43.28
rs11755724	G	A	0.019	0.004	1.70E-08	29.47
rs12549058	G	T	0.040	0.006	3.20E-10	39.06
rs1294421	G	T	0.025	0.003	6.90E-14	54.07
rs1316952	T	C	0.028	0.005	7.30E-09	32.65
rs1440372	C	T	0.021	0.004	7.60E-09	32.21
rs1563355	T	C	-0.031	0.004	1.70E-12	49.64
rs1569135	A	G	0.024	0.003	1.00E-12	52.89
rs16996700	T	C	0.021	0.004	1.60E-08	32.21
rs17109256	A	G	0.023	0.004	3.00E-08	31.47
rs17451107	T	C	0.023	0.004	3.50E-11	43.18
rs2075650	A	G	0.029	0.005	6.40E-09	35.03
rs2179129	A	G	0.021	0.003	1.20E-09	38.15
rs2207139	G	A	0.025	0.004	1.40E-08	32.28
rs2287019	C	T	0.026	0.005	4.30E-09	33.38
rs2745359	C	T	0.063	0.009	3.80E-13	52.44
rs2765539	C	T	-0.027	0.004	1.10E-12	50.48
rs2972164	C	T	0.019	0.003	2.40E-08	33.15
rs3786897	G	A	0.022	0.003	4.00E-11	41.87
rs459193	A	G	0.026	0.004	6.00E-12	46.81
rs4640244	G	A	0.021	0.004	3.10E-08	32.21
rs4929927	G	A	0.020	0.003	7.60E-09	34.60
rs904453	T	G	0.018	0.003	4.30E-08	28.03
rs929641	A	G	0.020	0.003	4.20E-09	36.73
rs9860730	A	G	0.023	0.004	2.80E-10	40.82
rs998584	A	C	0.029	0.004	5.00E-15	61.43
