# Supplementary material for: Identifying modifiable risk factors of lung cancer: Indications from Mendelian randomization
Source: PLoS One. 2021 Oct 18;16(10):e0258498. doi: 10.1371/journal.pone.0258498 (PMC8523078; doi:10.1371/journal.pone.0258498)
Supplement: S9 Table — The SNP is the result of genetic variants; A1 is the effect allele; A2 is the other allele; beta is the effect size of A1 on the exposure; she is the standard error of beta; pval is the p-value of beta; F is the F statistics. (PDF) [file pone.0258498.s022.pdf]

S9 Table: Instrumental variables of DBP. SNP is the rsID of genetic variants; A1 is the effect allele; A2 is the other allele; beta is the effect size of A1 on the exposure; se is the standard error of beta; pval is the p value of beta; F is the F statistics.

SNP	A1	A2	beta	se	pval	F
rs1000423	T	C	0.192	0.020	9.54E-22	91.68
rs10048404	T	C	-0.110	0.018	2.01E-09	35.87
rs10054208	T	C	0.119	0.019	1.49E-10	41.17
rs10062036	T	C	-0.152	0.022	2.13E-12	49.32
rs10062049	T	C	0.221	0.026	4.50E-18	74.98
rs1006545	T	G	0.363	0.028	7.96E-40	174.53
rs10066799	T	G	0.138	0.019	9.42E-13	50.98
rs10069690	T	C	0.162	0.021	1.42E-14	59.14
rs10164193	T	G	-0.220	0.033	1.87E-11	45.10
rs10170954	A	C	-0.105	0.018	6.90E-09	33.46
rs10244142	T	C	0.131	0.019	5.15E-12	47.61
rs10251493	T	C	0.126	0.023	3.92E-08	30.32
rs1025655	A	G	-0.108	0.018	1.32E-09	36.96
rs1035514	A	G	0.161	0.026	8.25E-10	37.62
rs1035673	T	C	0.163	0.018	3.00E-20	85.25
rs10424224	T	C	0.104	0.018	1.05E-08	32.78
rs10434099	A	C	0.105	0.018	4.42E-09	34.21
rs1043809	T	C	0.159	0.022	9.77E-13	50.84
rs1044822	T	C	-0.133	0.024	4.14E-08	30.14
rs10468291	A	C	-0.117	0.018	3.70E-11	43.89
rs1047891	A	C	-0.141	0.019	8.19E-14	55.85
rs10490923	A	G	0.153	0.026	5.02E-09	34.24
rs10493408	A	C	0.158	0.026	5.09E-10	38.59
rs10502214	T	C	0.125	0.021	9.40E-10	37.42
rs10740993	T	C	0.219	0.017	3.83E-36	157.69
rs10743086	A	G	-0.132	0.021	7.46E-10	37.87
rs10759344	T	C	0.195	0.032	9.34E-10	37.56
rs10759697	A	G	0.131	0.017	3.94E-14	57.16
rs10761530	T	C	0.117	0.017	1.14E-11	46.27
rs1077393	A	G	-0.248	0.018	2.32E-43	191.34
rs10776752	T	G	0.457	0.033	1.25E-43	192.03
rs1077795	A	G	0.199	0.020	1.62E-23	99.70
rs10804330	T	C	0.133	0.018	4.60E-14	57.19
rs10807003	T	G	-0.136	0.017	6.76E-15	60.91
rs10828399	A	G	-0.108	0.017	5.09E-10	38.68
rs10832013	T	G	0.152	0.019	4.51E-16	66.24
rs10832300	A	G	-0.130	0.018	2.11E-13	53.86
rs10832586	A	C	-0.308	0.022	2.53E-46	203.72
rs10835161	T	G	-0.139	0.018	1.01E-14	60.04

rs10835353	A	G	-0.108	0.017	4.50E-10	38.81
rs10838702	T	G	0.238	0.018	1.27E-40	178.03
rs10840378	T	C	0.278	0.022	3.33E-36	158.24
rs10850526	A	G	-0.202	0.019	1.61E-26	114.00
rs10864859	T	G	0.196	0.033	1.52E-09	36.44
rs10873612	T	C	-0.110	0.018	9.51E-10	37.49
rs10906391	T	C	0.129	0.019	7.57E-12	46.79
rs10941043	T	G	-0.127	0.019	2.52E-11	44.61
rs10980408	T	C	-0.375	0.048	4.17E-15	61.64
rs11001051	A	C	-0.108	0.020	3.25E-08	30.59
rs11026586	A	G	0.290	0.034	2.68E-17	71.39
rs11055034	A	C	-0.135	0.020	5.90E-12	47.57
rs11065129	A	G	0.137	0.018	6.30E-15	60.93
rs11070245	T	G	-0.129	0.017	1.57E-13	54.71
rs11077961	A	G	0.107	0.019	8.55E-09	33.28
rs11108209	T	C	-0.190	0.030	2.40E-10	40.15
rs1111462	A	G	-0.163	0.020	1.20E-15	64.24
rs111384899	T	C	-0.143	0.023	4.62E-10	38.87
rs11141731	T	C	-0.126	0.021	1.31E-09	36.93
rs1114347	A	G	-0.179	0.017	3.32E-25	107.30
rs11145807	A	G	0.155	0.018	4.10E-17	70.96
rs11153730	T	C	0.155	0.017	2.57E-19	80.38
rs11167039	A	G	-0.178	0.030	3.60E-09	34.89
rs111777102	T	C	0.214	0.035	1.56E-09	36.54
rs11187939	A	G	0.105	0.018	2.11E-09	35.73
rs11196549	A	G	0.445	0.045	2.94E-23	98.84
rs112280096	A	C	-0.119	0.019	6.95E-10	38.16
rs112332688	A	G	-0.114	0.021	4.43E-08	29.88
rs11252324	T	G	-0.234	0.033	1.03E-12	50.85
rs112559865	T	C	-0.276	0.042	5.59E-11	42.92
rs11259905	A	C	0.130	0.022	1.29E-09	36.79
rs113086489	T	C	0.179	0.018	4.40E-24	102.73
rs1133400	A	G	-0.132	0.022	8.30E-10	37.58
rs113783450	A	G	-0.126	0.019	4.75E-11	43.07
rs1138293	T	C	-0.128	0.022	7.05E-09	33.64
rs114503346	T	C	-0.268	0.043	3.10E-10	39.52
rs11466111	T	C	0.668	0.067	1.94E-23	99.43
rs115011309	T	C	0.185	0.031	2.55E-09	35.46
rs115245297	T	C	-0.312	0.044	1.06E-12	50.84
rs11543651	T	C	-0.116	0.019	6.64E-10	38.07
rs115521166	A	G	0.391	0.051	1.57E-14	59.10
rs11556924	T	C	-0.181	0.018	1.83E-23	100.00
rs11578696	A	G	0.146	0.026	1.65E-08	31.98
rs11592107	A	G	0.120	0.019	1.23E-10	41.39

rs116039813	T	G	0.356	0.059	1.33E-09	36.78
rs11605229	T	C	-0.247	0.028	2.40E-18	76.53
rs11608122	T	C	0.124	0.020	2.97E-10	39.88
rs116287796	T	C	-0.423	0.066	1.00E-10	41.77
rs11636952	T	C	0.400	0.019	5.21E-99	447.24
rs11661473	A	G	0.201	0.020	1.54E-24	104.85
rs11721984	T	C	-0.141	0.018	1.89E-15	63.37
rs117324780	A	G	-0.275	0.049	2.48E-08	31.13
rs117464403	A	G	0.454	0.068	2.95E-11	44.26
rs11778153	T	C	0.119	0.018	5.84E-11	42.90
rs117785419	A	G	0.381	0.069	2.99E-08	30.70
rs11781001	A	G	0.155	0.024	1.11E-10	41.55
rs117828113	T	C	0.235	0.035	3.52E-11	43.88
rs1178979	T	C	0.150	0.022	9.96E-12	46.31
rs11837982	A	C	0.150	0.018	2.14E-16	67.37
rs11853359	A	G	-0.166	0.018	1.30E-19	82.28
rs11854184	A	C	0.134	0.021	2.32E-10	40.09
rs11859505	A	G	-0.104	0.018	9.76E-09	32.82
rs11923343	A	G	-0.114	0.018	3.10E-10	39.53
rs11945489	T	C	-0.139	0.019	3.99E-13	52.56
rs11960210	T	C	0.247	0.018	3.36E-43	188.91
rs11961593	T	C	-0.316	0.035	1.49E-19	81.88
rs11966966	T	C	0.251	0.020	3.10E-36	158.58
rs11984138	A	G	0.134	0.021	2.13E-10	40.27
rs12110481	T	G	0.159	0.019	7.37E-17	69.56
rs12114418	A	G	-0.148	0.021	1.13E-12	50.77
rs12142296	T	G	-0.164	0.025	8.89E-11	42.17
rs12143164	T	C	-0.100	0.018	4.62E-08	29.98
rs1215469	A	C	-0.138	0.021	5.23E-11	42.96
rs12192720	A	G	-0.109	0.019	1.18E-08	32.63
rs12216886	T	G	0.129	0.022	4.76E-09	34.18
rs12323265	A	G	-0.116	0.018	1.00E-10	42.00
rs1232482	T	C	-0.121	0.018	6.10E-12	47.58
rs12337056	T	C	0.136	0.023	2.18E-09	35.79
rs12405515	T	G	-0.170	0.017	1.92E-22	95.23
rs12426178	A	C	0.172	0.024	8.34E-13	51.00
rs1243876	T	C	-0.106	0.019	2.14E-08	31.30
rs12446456	T	C	-0.181	0.018	3.99E-25	106.97
rs12486605	T	C	-0.151	0.018	1.01E-17	73.41
rs12496226	T	G	0.146	0.019	5.32E-15	61.12
rs12509595	T	C	-0.497	0.019	1.58E-148	670.59
rs12515541	T	G	0.116	0.018	6.23E-11	42.65
rs12536606	T	C	-0.123	0.020	3.18E-10	39.79

rs12555129	T	G	-0.169	0.022	1.31E-14	59.27
rs12574332	T	C	0.207	0.027	6.14E-15	60.68
rs12609484	T	G	-0.140	0.019	1.16E-13	55.30
rs12623637	A	G	-0.102	0.018	5.96E-09	33.79
rs12630496	T	C	-0.116	0.018	1.81E-10	40.83
rs1263671	T	C	-0.139	0.024	4.69E-09	34.31
rs1265842	T	C	0.111	0.017	1.70E-10	40.92
rs12665161	A	G	0.121	0.018	1.63E-11	45.19
rs12693302	A	G	-0.238	0.018	2.16E-39	172.61
rs12703989	A	G	0.103	0.018	4.35E-09	34.64
rs1271309	A	G	-0.198	0.024	1.45E-16	67.99
rs12728150	A	G	-0.205	0.032	1.28E-10	41.36
rs12730750	A	G	0.112	0.019	1.95E-09	35.94
rs1275988	T	C	-0.295	0.018	1.92E-62	276.84
rs12790943	T	C	-0.100	0.018	1.14E-08	32.78
rs12809048	T	C	0.232	0.042	2.21E-08	31.36
rs12866098	A	G	0.103	0.019	2.73E-08	30.84
rs12906962	T	C	-0.238	0.019	8.73E-37	160.00
rs12919839	T	C	-0.110	0.019	1.04E-08	32.70
rs12929303	A	G	0.157	0.017	1.58E-19	81.62
rs12983238	A	G	-0.127	0.020	3.23E-10	39.67
rs12990959	T	C	-0.127	0.019	1.11E-11	46.20
rs13001283	A	G	0.152	0.024	1.92E-10	40.55
rs13014371	T	C	-0.118	0.018	1.68E-11	45.16
rs13042148	T	C	-0.167	0.024	7.24E-12	47.07
rs13044147	T	G	-0.231	0.040	5.88E-09	33.89
rs13107325	T	C	-0.675	0.034	3.72E-88	396.12
rs13139571	A	C	-0.241	0.020	2.29E-32	140.71
rs13149808	T	C	-0.102	0.019	4.45E-08	29.93
rs13179413	T	C	0.125	0.020	3.04E-10	39.86
rs13195550	T	C	0.132	0.019	1.69E-12	49.83
rs13215166	A	G	-0.309	0.017	1.79E-70	316.19
rs1322639	A	G	-0.158	0.021	3.87E-14	57.44
rs13240040	A	G	0.119	0.019	3.98E-10	38.96
rs13270541	A	G	0.120	0.021	1.33E-08	32.34
rs1327235	A	G	-0.302	0.017	4.76E-68	304.33
rs13358657	A	G	-0.224	0.026	1.70E-18	77.16
rs134041	T	C	0.122	0.018	3.05E-12	48.84
rs137923903	T	C	-0.818	0.084	1.59E-22	95.38
rs1422278	T	G	0.232	0.025	2.28E-20	85.73
rs142449193	T	C	-0.257	0.043	1.51E-09	36.48
rs1425486	T	C	-0.133	0.019	1.11E-12	50.66
rs1436138	A	G	0.199	0.018	7.33E-28	119.67
rs145211473	A	G	0.362	0.063	1.14E-08	32.62

rs1454393	A	C	-0.101	0.017	4.54E-09	34.29
rs1465249	T	C	0.096	0.018	3.56E-08	30.34
rs1468520	A	G	-0.164	0.023	2.49E-12	49.00
rs148401029	A	C	-0.312	0.049	1.32E-10	41.27
rs148775688	T	C	0.383	0.068	1.40E-08	32.14
rs1490384	T	C	0.098	0.017	1.59E-08	31.89
rs149731397	A	G	0.552	0.085	8.52E-11	42.11
rs150816167	T	C	-0.287	0.045	1.17E-10	41.50
rs152130	A	G	-0.118	0.019	9.70E-10	37.52
rs1536608	T	G	0.106	0.018	1.70E-09	36.34
rs1543310	T	C	-0.139	0.018	7.31E-15	60.56
rs1561719	T	C	-0.149	0.025	1.77E-09	36.29
rs1565716	A	G	0.214	0.034	3.45E-10	39.35
rs1571737	T	C	0.130	0.023	1.45E-08	32.23
rs1582931	A	G	0.216	0.018	4.51E-35	152.49
rs162887	A	G	0.112	0.019	3.87E-09	34.62
rs1641547	A	G	0.146	0.018	2.83E-16	67.18
rs167479	T	G	-0.362	0.019	1.67E-82	370.77
rs16848690	T	C	0.183	0.029	1.90E-10	40.51
rs16852778	T	C	0.161	0.024	4.03E-11	43.68
rs16853084	T	C	-0.272	0.049	2.57E-08	30.98
rs1687295	T	C	0.206	0.019	2.99E-26	112.86
rs16875357	T	G	-0.121	0.020	2.70E-09	35.24
rs1688796	T	C	0.136	0.020	1.15E-11	45.97
rs17004519	A	G	0.365	0.066	3.48E-08	30.42
rs17224476	A	G	0.160	0.028	7.37E-09	33.48
rs1727311	T	G	-0.203	0.022	3.96E-20	84.37
rs17321041	T	C	0.231	0.036	1.78E-10	40.60
rs17361679	T	C	0.164	0.029	1.52E-08	32.12
rs17362588	A	G	0.333	0.031	9.27E-28	119.20
rs17376426	T	C	0.313	0.050	3.42E-10	39.45
rs17396055	A	G	-0.115	0.018	4.13E-10	39.06
rs17422386	A	G	0.262	0.044	3.13E-09	35.11
rs17454517	A	G	0.122	0.017	2.65E-12	48.84
rs17473424	A	G	0.273	0.029	1.23E-21	91.12
rs17611141	T	G	-0.383	0.043	8.88E-19	78.20
rs17626956	A	G	-0.340	0.053	1.38E-10	41.24
rs17880989	A	G	0.401	0.059	1.11E-11	46.13
rs1807870	A	G	0.103	0.018	5.87E-09	33.99
rs1819663	A	G	0.115	0.017	4.63E-11	43.45
rs1821295	T	C	-0.138	0.019	3.13E-13	53.31
rs1842646	A	C	0.098	0.018	2.65E-08	30.91
rs1848510	A	G	0.126	0.018	4.10E-12	48.15
rs1861881	T	G	0.115	0.019	6.49E-10	38.23

rs1863798	A	G	-0.100	0.018	3.26E-08	30.59
rs1867624	T	C	0.141	0.018	2.08E-15	62.93
rs1871190	T	G	0.108	0.019	6.63E-09	33.59
rs1876490	A	G	0.136	0.019	1.16E-12	50.47
rs1879056	T	C	-0.216	0.022	6.19E-23	97.19
rs1882961	T	C	0.127	0.019	1.40E-11	45.78
rs189110	A	G	-0.256	0.045	1.56E-08	32.04
rs1906672	A	G	0.140	0.021	8.48E-12	46.77
rs1947228	T	C	-0.145	0.018	2.59E-16	66.93
rs1950500	T	C	0.140	0.019	2.20E-13	53.98
rs195486	T	C	0.121	0.018	1.18E-11	46.13
rs1969539	A	G	0.098	0.017	1.68E-08	31.76
rs1980607	A	G	0.123	0.019	1.51E-10	40.91
rs1984195	A	G	0.174	0.017	1.43E-23	100.69
rs1986971	A	G	0.105	0.019	4.60E-08	29.74
rs198851	T	G	0.389	0.024	2.93E-57	254.04
rs1996992	T	G	-0.297	0.039	4.71E-14	56.71
rs2009733	A	G	0.122	0.018	5.10E-12	47.81
rs2012831	A	G	0.107	0.019	1.83E-08	31.60
rs2060664	T	C	0.109	0.020	4.65E-08	29.76
rs2065152	T	C	0.110	0.018	8.95E-10	37.55
rs2065498	T	G	-0.146	0.023	4.19E-10	39.00
rs2085600	A	G	-0.131	0.023	7.00E-09	33.45
rs2098839	T	C	0.175	0.018	1.31E-21	91.34
rs2105388	A	C	0.140	0.018	1.28E-15	63.91
rs2146315	T	C	-0.120	0.021	5.03E-09	34.09
rs2162003	T	C	0.128	0.018	3.20E-12	48.85
rs2165702	A	G	-0.142	0.018	2.01E-15	63.02
rs217209	T	C	0.157	0.025	3.53E-10	39.50
rs2173957	A	G	0.108	0.018	2.09E-09	35.87
rs223361	T	C	0.170	0.018	2.70E-20	85.16
rs2236295	T	G	-0.207	0.018	1.42E-31	136.77
rs2239268	A	G	0.110	0.019	7.40E-09	33.34
rs2240075	A	G	-0.148	0.020	4.32E-14	56.94
rs2242338	A	C	0.346	0.033	1.25E-25	109.46
rs2252865	T	C	0.119	0.018	5.51E-11	43.15
rs2269579	A	G	-0.177	0.029	1.09E-09	37.30
rs2273171	T	C	-0.098	0.017	1.30E-08	32.35
rs227426	T	G	0.112	0.018	1.75E-10	40.89
rs2282823	T	C	0.135	0.024	3.03E-08	30.61
rs2288464	A	C	-0.168	0.029	1.04E-08	32.69
rs2289123	T	G	-0.119	0.022	2.91E-08	30.63
rs2298807	T	C	0.123	0.021	4.89E-09	34.15
rs2306363	T	G	-0.264	0.022	1.63E-34	149.72

rs2307111	T	C	-0.174	0.018	1.62E-22	95.78
rs234614	T	G	-0.154	0.019	1.58E-15	63.34
rs2384061	A	G	-0.174	0.018	2.25E-23	98.97
rs2425757	T	C	-0.108	0.019	1.14E-08	32.70
rs2439371	A	C	0.135	0.024	1.30E-08	32.40
rs2450128	A	G	-0.151	0.024	3.53E-10	39.32
rs2478835	T	C	-0.109	0.018	5.04E-10	38.57
rs2484294	A	G	0.317	0.020	1.17E-58	260.76
rs2493288	A	G	0.254	0.025	1.97E-23	99.69
rs2513877	A	G	-0.129	0.022	4.22E-09	34.60
rs2546963	T	C	0.097	0.018	3.66E-08	30.44
rs2569882	T	C	0.120	0.018	4.28E-11	43.40
rs2586408	A	G	0.115	0.020	4.09E-09	34.60
rs2586970	A	G	-0.149	0.018	1.56E-17	72.79
rs2589254	A	G	-0.121	0.020	1.11E-09	37.22
rs2601831	T	C	-0.166	0.027	7.84E-10	37.85
rs2627316	A	G	-0.154	0.017	7.37E-19	78.74
rs2629665	A	C	-0.119	0.018	1.52E-11	45.43
rs2638395	T	G	0.126	0.021	9.88E-10	37.17
rs2643826	T	C	0.186	0.018	2.83E-26	112.60
rs2680663	A	G	-0.178	0.019	7.33E-22	92.47
rs2681485	A	G	0.295	0.018	1.31E-62	279.99
rs2693560	A	G	-0.150	0.018	1.09E-16	68.77
rs2695258	T	C	0.116	0.021	4.35E-08	30.02
rs2744133	A	G	0.144	0.019	1.17E-13	55.28
rs2801008	T	G	-0.105	0.019	2.14E-08	31.41
rs28362590	T	G	0.124	0.020	8.70E-10	37.43
rs2836411	T	C	0.156	0.018	1.72E-17	72.07
rs28377357	A	G	-0.124	0.019	6.03E-11	42.80
rs28429256	A	G	0.164	0.019	2.83E-18	75.73
rs28453037	A	C	-0.179	0.022	9.08E-16	64.72
rs28498052	A	G	-0.255	0.043	3.53E-09	34.79
rs28544928	T	G	0.154	0.020	9.13E-15	60.12
rs28564120	A	G	-0.125	0.022	1.22E-08	32.42
rs28570096	T	C	0.140	0.019	1.15E-13	55.14
rs28661492	T	C	-0.136	0.022	9.56E-10	37.47
rs28675079	A	G	-0.144	0.022	8.34E-11	42.31
rs2888691	A	G	-0.194	0.024	2.33E-15	62.89
rs2906152	A	G	-0.187	0.018	5.55E-25	107.08
rs2921604	T	C	-0.096	0.018	4.46E-08	29.75
rs2925345	T	C	0.189	0.017	1.60E-27	117.98
rs2927071	T	C	-0.139	0.019	6.43E-13	51.48
rs2940928	A	G	-0.126	0.022	7.56E-09	33.31
rs2957468	A	G	0.138	0.019	8.43E-14	55.40

rs297690	T	G	-0.130	0.020	5.54E-11	42.91
rs2978098	A	C	0.155	0.018	1.33E-18	77.36
rs2994648	T	C	0.131	0.022	3.33E-09	34.82
rs2999159	A	G	-0.306	0.023	1.79E-40	177.24
rs303217	T	C	-0.096	0.018	4.06E-08	30.09
rs306209	A	G	0.109	0.020	2.52E-08	31.10
rs3107147	A	G	0.106	0.018	3.26E-09	35.07
rs311564	A	G	-0.133	0.018	4.23E-13	52.82
rs3117736	T	C	0.237	0.020	9.71E-34	146.71
rs3118904	A	G	0.116	0.018	1.75E-10	40.69
rs314376	A	G	-0.136	0.017	3.79E-15	62.16
rs318719	T	C	-0.243	0.033	3.16E-13	52.98
rs3213545	A	G	0.111	0.020	1.06E-08	32.58
rs334414	T	C	-0.102	0.018	1.64E-08	32.01
rs33996239	T	C	-0.251	0.038	2.86E-11	44.20
rs34029821	A	G	-0.167	0.019	6.97E-18	74.01
rs34130368	T	G	-0.203	0.028	8.77E-13	50.94
rs34163044	A	C	0.149	0.018	9.63E-17	68.92
rs34196696	T	C	0.134	0.021	1.86E-10	40.53
rs342130	A	G	0.110	0.018	6.17E-10	38.40
rs34245489	T	C	0.242	0.042	8.00E-09	33.25
rs34297584	A	G	0.489	0.064	2.78E-14	57.93
rs342989	A	G	0.163	0.021	3.05E-15	62.08
rs34517439	A	C	-0.251	0.028	2.02E-19	81.19
rs34678172	A	G	-0.172	0.021	8.28E-17	69.39
rs347585	T	C	0.151	0.019	1.57E-15	63.49
rs35091929	T	C	0.183	0.018	6.46E-25	106.66
rs35100429	A	C	-0.096	0.018	3.46E-08	30.28
rs35213536	T	G	0.204	0.021	2.54E-23	99.42
rs35287509	T	C	-0.108	0.018	4.17E-09	34.58
rs35413927	A	G	-0.127	0.019	1.77E-11	45.44
rs35552228	T	G	-0.118	0.021	3.09E-08	30.53
rs35783704	A	G	-0.213	0.029	2.28E-13	53.68
rs35927325	T	C	0.222	0.036	1.01E-09	37.23
rs36092215	A	G	-0.265	0.046	7.25E-09	33.48
rs36152087	A	G	-0.131	0.018	2.73E-13	53.13
rs3735318	A	G	-0.101	0.017	4.72E-09	34.35
rs3735533	T	C	-0.487	0.033	6.32E-49	216.47
rs3744010	A	G	-0.143	0.020	2.01E-12	49.41
rs3766694	T	C	0.110	0.018	4.07E-10	39.28
rs3771300	T	G	-0.098	0.017	1.72E-08	31.76
rs3772219	A	C	0.175	0.019	2.94E-21	89.89
rs3774702	A	G	0.147	0.023	1.18E-10	41.57
rs3776299	A	G	0.127	0.018	5.06E-13	52.33

rs3782480	T	G	0.151	0.027	3.35E-08	30.47
rs3795320	T	G	-0.118	0.020	4.44E-09	34.29
rs3798293	A	G	-0.133	0.021	2.70E-10	39.99
rs3802230	A	C	-0.161	0.017	2.75E-20	85.08
rs3807101	T	C	-0.174	0.027	4.57E-11	43.26
rs3821843	A	G	0.171	0.019	5.39E-19	78.95
rs3861113	A	C	0.213	0.032	3.95E-11	43.59
rs387865	T	C	-0.106	0.019	3.17E-08	30.74
rs3898618	T	C	-0.268	0.038	2.49E-12	48.96
rs3918226	T	C	0.612	0.033	5.31E-77	345.69
rs39281	A	G	0.128	0.018	3.97E-13	52.46
rs3943093	T	C	0.248	0.018	3.95E-41	181.22
rs3996330	A	C	-0.129	0.018	4.00E-13	52.79
rs4007357	A	G	0.102	0.019	4.23E-08	29.87
rs404682	A	G	0.106	0.018	1.49E-09	36.69
rs4074812	A	G	-0.134	0.018	2.07E-14	58.28
rs4077158	T	C	-0.183	0.017	3.09E-26	112.14
rs41267086	A	G	0.265	0.040	2.90E-11	44.14
rs4141663	T	C	-0.150	0.018	1.41E-17	73.08
rs41475048	A	G	-0.123	0.020	9.93E-10	37.26
rs41525648	A	G	0.145	0.018	2.48E-16	66.83
rs4240358	A	G	0.126	0.021	4.23E-09	34.39
rs425011	A	G	-0.132	0.019	1.10E-11	46.16
rs4270476	T	C	0.118	0.018	5.24E-11	43.27
rs4300482	A	G	-0.101	0.018	4.64E-08	29.83
rs4315061	T	C	0.125	0.018	2.24E-12	49.00
rs4320727	A	G	0.143	0.018	1.80E-15	63.11
rs4362428	A	C	-0.113	0.018	1.45E-10	41.00
rs4420291	A	G	0.097	0.017	2.17E-08	31.14
rs4421757	A	G	0.098	0.017	1.63E-08	31.79
rs4424827	T	C	-0.098	0.018	2.11E-08	31.42
rs4507125	A	C	-0.124	0.021	3.60E-09	34.76
rs45474499	T	C	0.356	0.042	8.50E-18	73.67
rs45572038	T	C	0.356	0.051	2.27E-12	49.19
rs4615669	A	G	-0.114	0.017	6.10E-11	42.93
rs4634143	T	C	0.116	0.019	7.89E-10	37.73
rs4651224	T	C	0.110	0.018	3.39E-10	39.65
rs4675297	A	G	-0.128	0.018	3.16E-12	48.70
rs4675682	T	C	-0.141	0.017	4.49E-16	66.33
rs4704514	T	C	0.109	0.019	1.71E-08	31.72
rs4705695	T	C	-0.144	0.017	1.18E-16	68.78
rs4709746	T	C	-0.145	0.026	2.21E-08	31.21
rs4713650	T	C	-0.120	0.018	2.23E-11	44.67
rs4725579	A	C	0.146	0.022	2.96E-11	44.14

rs4729628	T	C	-0.185	0.031	3.11E-09	35.01
rs4761524	A	G	0.096	0.017	2.77E-08	30.86
rs4773140	A	G	-0.139	0.021	2.90E-11	44.34
rs4788444	A	G	-0.145	0.024	1.36E-09	36.76
rs4800420	A	G	0.119	0.019	5.18E-10	38.54
rs4808046	A	G	-0.140	0.021	1.30E-11	45.68
rs4810846	T	C	0.142	0.018	8.98E-16	64.73
rs481795	T	C	0.168	0.024	1.81E-12	49.59
rs4818833	A	G	-0.156	0.018	1.93E-18	77.01
rs4837127	T	C	0.117	0.021	2.61E-08	31.09
rs4864421	T	C	-0.127	0.018	8.75E-13	51.32
rs4873492	T	C	0.140	0.023	1.28E-09	36.78
rs4875958	A	G	0.106	0.019	4.39E-08	29.94
rs4891258	A	G	-0.116	0.019	5.72E-10	38.41
rs4903064	T	C	0.154	0.021	7.84E-14	56.10
rs4908671	A	C	0.108	0.018	1.36E-09	36.68
rs4924570	T	C	-0.169	0.018	9.62E-21	87.39
rs4926923	T	C	0.192	0.031	4.75E-10	38.78
rs4932373	A	C	-0.366	0.019	7.71E-84	375.83
rs4936099	A	C	0.175	0.018	1.16E-22	96.11
rs4948550	T	C	-0.111	0.019	1.00E-08	32.97
rs4948643	T	C	0.159	0.019	2.26E-16	67.26
rs4952668	A	G	-0.192	0.018	1.13E-26	113.78
rs4954192	T	C	-0.123	0.018	8.15E-12	46.83
rs4974072	A	G	0.105	0.017	1.63E-09	36.28
rs4984496	T	G	0.176	0.019	4.93E-21	88.88
rs4998802	A	G	-0.107	0.020	4.42E-08	29.91
rs5025116	T	C	0.096	0.018	4.97E-08	29.57
rs504217	T	C	0.275	0.034	2.51E-16	67.14
rs504691	A	C	-0.118	0.018	3.14E-11	44.22
rs507666	A	G	-0.285	0.022	2.27E-37	163.79
rs521033	A	G	-0.180	0.025	1.10E-12	50.73
rs55641580	T	C	0.175	0.027	4.79E-11	43.36
rs55684003	A	G	0.122	0.019	1.01E-10	41.67
rs55706574	A	G	-0.115	0.019	3.20E-09	35.20
rs55710016	A	G	0.116	0.017	2.72E-11	44.21
rs55747751	A	G	-0.224	0.033	1.39E-11	45.76
rs55770741	T	C	-0.128	0.018	2.20E-13	53.58
rs55857306	A	G	-0.522	0.024	5.05E-109	494.16
rs55935819	A	G	0.127	0.018	1.96E-12	49.31
rs55938136	A	G	0.141	0.023	4.21E-10	39.16
rs55944332	A	G	-0.237	0.020	3.27E-31	134.40
rs55993676	T	G	-0.210	0.019	3.82E-28	120.54

rs56074814	T	C	0.124	0.020	4.96E-10	38.69
rs56233017	A	G	-0.274	0.044	4.37E-10	38.86
rs56254331	A	C	-0.145	0.025	3.43E-09	34.98
rs56256111	A	G	0.193	0.026	2.60E-13	53.63
rs56335308	A	G	0.355	0.057	6.31E-10	38.25
rs56345595	A	G	0.133	0.018	5.20E-14	56.38
rs56388530	T	C	0.229	0.021	7.45E-29	123.68
rs56401299	T	C	0.100	0.018	4.50E-08	30.10
rs56411469	A	C	0.206	0.032	7.22E-11	42.56
rs568032	A	G	-0.267	0.039	6.28E-12	47.15
rs56810307	T	C	-0.155	0.027	1.29E-08	32.24
rs569550	T	G	-0.269	0.018	1.23E-49	220.55
rs57091267	A	G	-0.180	0.022	2.72E-16	66.87
rs57327054	T	C	-0.117	0.019	8.07E-10	37.72
rs57361399	T	C	-0.165	0.021	1.20E-15	64.16
rs57490543	A	C	0.169	0.030	1.22E-08	32.53
rs57786342	A	G	0.142	0.022	4.37E-11	43.40
rs588273	A	G	-0.115	0.018	7.85E-11	42.55
rs5992929	T	C	0.168	0.019	3.07E-18	76.13
rs60143695	T	C	-0.174	0.028	6.64E-10	38.20
rs6021247	A	G	0.130	0.017	8.21E-14	55.56
rs6031431	A	G	-0.115	0.018	4.94E-11	43.41
rs6039211	A	G	0.178	0.018	6.82E-23	97.15
rs6040421	A	G	0.096	0.017	4.17E-08	30.12
rs604723	T	C	-0.385	0.019	2.32E-87	393.43
rs6060262	A	C	0.180	0.024	4.80E-14	56.60
rs6062295	A	G	-0.120	0.018	1.17E-11	45.81
rs6062533	A	G	-0.144	0.021	3.32E-12	48.66
rs6076983	T	C	0.125	0.018	1.61E-12	49.87
rs6081555	T	G	-0.102	0.018	3.06E-08	30.76
rs61408836	T	C	-0.128	0.022	5.69E-09	34.00
rs61772592	A	G	-0.151	0.026	7.42E-09	33.43
rs61789369	A	G	-0.304	0.044	3.07E-12	48.58
rs61879810	A	G	0.165	0.024	1.35E-11	45.83
rs61926149	T	G	0.518	0.091	1.30E-08	32.31
rs61932870	T	G	-0.418	0.054	1.42E-14	59.15
rs62004794	A	G	-0.096	0.017	3.38E-08	30.57
rs62011935	T	C	0.136	0.022	2.80E-10	39.82
rs62012629	A	C	-0.186	0.021	2.41E-19	80.91
rs62030049	A	G	0.134	0.021	1.55E-10	40.86
rs62155750	A	G	-0.218	0.020	8.27E-29	123.37
rs62158170	A	G	0.165	0.021	6.63E-15	60.78
rs62169544	A	G	-0.121	0.018	4.96E-12	47.57
rs62250937	T	C	0.186	0.032	6.35E-09	33.82

rs62264113	A	G	0.153	0.028	4.66E-08	29.86
rs62301873	A	G	-0.173	0.028	1.06E-09	37.28
rs62380354	A	C	0.183	0.029	3.68E-10	39.33
rs62413470	A	G	-0.166	0.024	9.07E-12	46.61
rs62434124	T	C	-0.485	0.034	7.83E-47	206.15
rs62503324	T	C	0.203	0.020	2.11E-23	99.31
rs62505281	A	G	0.114	0.020	6.08E-09	33.77
rs6271	T	C	-0.431	0.035	1.72E-34	150.13
rs6442105	A	G	-0.249	0.019	3.10E-41	180.43
rs6442608	T	C	-0.134	0.023	7.49E-09	33.31
rs645144	T	C	0.101	0.019	4.15E-08	30.04
rs645394	A	G	-0.099	0.018	1.95E-08	31.45
rs6457718	A	G	-0.117	0.018	7.96E-11	42.32
rs6458374	T	C	0.155	0.022	2.33E-12	49.38
rs6464165	T	C	-0.217	0.020	7.34E-29	123.84
rs6504163	T	C	-0.184	0.018	6.30E-24	101.32
rs650724	A	G	-0.197	0.031	1.30E-10	41.30
rs6511291	T	C	-0.116	0.018	6.89E-11	42.80
rs6544610	A	G	0.136	0.021	9.52E-11	41.76
rs6546810	T	C	-0.120	0.018	3.16E-11	43.95
rs6547850	T	G	-0.111	0.017	2.07E-10	40.55
rs6555205	T	C	-0.097	0.018	3.84E-08	30.41
rs6555948	A	G	0.096	0.018	4.71E-08	29.75
rs6565174	A	C	-0.195	0.028	2.42E-12	49.20
rs6580970	T	C	-0.166	0.019	4.03E-18	75.63
rs668459	T	C	-0.113	0.018	1.01E-10	41.84
rs6686889	T	C	0.192	0.020	6.95E-22	92.89
rs66887589	T	C	-0.161	0.017	1.83E-20	85.62
rs6708660	T	C	0.117	0.018	3.97E-11	43.47
rs67535236	A	G	0.168	0.023	1.39E-13	54.77
rs6758859	T	C	0.121	0.018	1.45E-11	45.77
rs6776964	A	G	0.097	0.017	1.86E-08	31.63
rs6777317	A	G	0.125	0.020	1.51E-10	41.03
rs6779368	A	G	-0.179	0.018	2.28E-22	94.74
rs6807945	T	C	-0.187	0.024	1.80E-15	63.05
rs68085857	T	C	0.191	0.021	9.83E-21	86.81
rs6819297	T	G	0.108	0.019	9.27E-09	33.05
rs682681	T	C	-0.145	0.019	4.47E-15	61.77
rs6838416	A	G	-0.121	0.019	8.21E-11	42.01
rs6875967	A	G	0.134	0.018	1.21E-13	55.14
rs687914	T	G	0.169	0.020	6.49E-17	69.83
rs6889240	A	G	0.120	0.020	1.05E-09	37.29
rs6892983	A	C	0.200	0.018	9.45E-30	128.62
rs6905288	A	G	0.176	0.018	7.79E-23	96.57

rs6912283	A	G	-0.133	0.017	2.49E-14	57.99
rs693974	T	C	-0.185	0.018	1.76E-25	108.89
rs6957161	A	G	0.127	0.020	1.34E-10	41.21
rs699	A	G	-0.236	0.018	1.30E-40	177.63
rs705696	A	G	-0.105	0.018	1.21E-08	32.32
rs7084783	A	G	0.113	0.017	7.77E-11	42.47
rs7090758	T	C	-0.153	0.017	6.89E-19	78.52
rs7098414	A	C	0.112	0.020	1.03E-08	32.77
rs7116797	A	G	0.184	0.028	5.19E-11	43.02
rs71326977	A	C	-0.137	0.023	2.75E-09	35.38
rs7134060	A	G	-0.106	0.017	1.13E-09	36.97
rs7137749	T	C	0.141	0.018	7.23E-15	60.86
rs7137828	T	C	-0.503	0.018	4.80E-180	815.82
rs71409374	A	G	-0.252	0.039	5.95E-11	42.81
rs714785	A	G	-0.265	0.047	1.53E-08	32.01
rs7151887	A	G	0.121	0.020	3.14E-09	34.95
rs715299	T	G	0.169	0.020	1.10E-17	73.25
rs7155504	T	C	0.229	0.032	5.16E-13	52.00
rs7169864	T	C	-0.113	0.021	3.40E-08	30.49
rs7171498	T	C	-0.114	0.018	6.23E-11	42.66
rs7184016	T	G	0.111	0.020	4.43E-08	29.84
rs7213273	A	G	-0.180	0.018	3.58E-23	98.03
rs7215084	T	C	0.112	0.017	1.17E-10	41.61
rs7217916	A	G	0.111	0.018	5.63E-10	38.52
rs7221807	T	C	-0.121	0.018	5.40E-12	47.34
rs7227492	T	C	0.181	0.023	1.43E-15	63.58
rs722783	A	G	-0.209	0.021	9.03E-24	101.25
rs7235890	T	G	-0.169	0.029	4.12E-09	34.52
rs7257694	T	C	0.184	0.018	6.28E-25	106.51
rs72639047	A	G	-0.153	0.026	2.29E-09	35.67
rs72663521	A	G	0.126	0.022	1.42E-08	32.26
rs72683923	T	C	0.533	0.064	5.02E-17	70.32
rs72719149	T	C	-0.128	0.019	6.34E-12	47.28
rs72759896	A	G	0.159	0.028	1.06E-08	32.63
rs72827873	T	C	0.181	0.030	9.05E-10	37.56
rs72831343	T	G	0.494	0.025	4.77E-88	396.14
rs72842207	T	C	-0.211	0.021	1.10E-23	100.19
rs72902690	A	G	0.148	0.023	8.62E-11	41.94
rs72936986	A	C	0.114	0.019	3.33E-09	34.89
rs72976750	T	C	-0.172	0.025	7.37E-12	46.85
rs72999033	T	C	0.279	0.036	5.95E-15	60.87
rs73033340	A	G	0.531	0.053	5.06E-24	102.38
rs73046792	A	G	-0.152	0.025	5.87E-10	38.39

rs7306523	A	G	0.138	0.024	1.25E-08	32.34
rs73075659	A	G	0.251	0.019	6.69E-42	183.93
rs73105827	T	G	-0.188	0.031	2.07E-09	36.00
rs73174353	T	C	0.446	0.078	1.28E-08	32.37
rs7321688	A	C	0.151	0.021	1.99E-13	54.04
rs73280613	A	G	-0.216	0.038	1.75E-08	31.69
rs7350752	A	G	-0.150	0.027	1.97E-08	31.49
rs7387891	A	G	0.108	0.019	4.73E-09	34.33
rs74048200	A	G	-0.197	0.033	1.47E-09	36.67
rs7407	T	C	-0.134	0.018	8.81E-14	55.75
rs7418070	T	C	-0.141	0.019	1.30E-13	55.07
rs7427249	A	G	-0.110	0.018	4.34E-10	38.92
rs74439044	T	C	-0.350	0.029	1.38E-32	141.40
rs74734425	T	C	0.404	0.042	3.52E-22	93.77
rs7487608	T	G	-0.141	0.018	1.77E-15	63.46
rs7500448	A	G	-0.130	0.020	1.14E-10	41.61
rs7513240	T	C	-0.109	0.018	3.11E-09	35.29
rs751984	T	C	0.394	0.028	1.38E-46	204.96
rs7523897	A	C	0.133	0.024	2.77E-08	30.78
rs7524019	T	C	0.104	0.017	2.60E-09	35.45
rs7548671	T	C	0.111	0.018	3.28E-10	39.42
rs75507123	T	G	-0.143	0.026	3.94E-08	30.19
rs75511781	A	G	-0.372	0.047	2.45E-15	62.68
rs7576060	T	C	-0.102	0.018	2.05E-08	31.57
rs7592578	T	G	-0.200	0.022	4.71E-19	79.56
rs7606205	A	C	-0.128	0.019	2.36E-11	44.63
rs76073047	A	G	0.230	0.042	2.84E-08	30.80
rs7608483	A	C	0.117	0.018	2.83E-11	44.27
rs7611674	T	G	0.158	0.022	1.67E-12	49.95
rs76164690	T	G	-0.154	0.025	7.19E-10	37.95
rs7623706	A	G	0.098	0.018	2.84E-08	30.69
rs762384	T	C	0.151	0.021	1.97E-13	54.26
rs76304369	T	C	0.461	0.079	4.92E-09	34.20
rs76326501	A	C	0.362	0.031	2.17E-32	140.71
rs76452347	T	C	-0.225	0.023	9.37E-23	96.19
rs76785130	A	G	-0.429	0.066	9.36E-11	41.90
rs7694643	A	G	-0.132	0.018	3.07E-13	53.02
rs76954792	T	C	0.121	0.021	5.06E-09	34.01
rs7708525	T	C	-0.136	0.021	4.12E-11	43.52
rs77151571	T	C	0.331	0.051	8.11E-11	42.20
rs7731530	A	C	0.136	0.019	6.41E-13	51.46
rs77375686	A	G	-0.205	0.028	1.77E-13	54.32
rs7753695	T	C	0.103	0.018	4.77E-09	34.32
rs7763102	A	C	0.112	0.020	2.61E-08	30.99

rs7788746	T	G	-0.164	0.018	3.19E-19	80.71
rs77924615	A	G	-0.316	0.022	3.72E-45	199.39
rs78151625	T	C	-0.187	0.023	1.04E-15	64.34
rs7817000	A	C	0.107	0.019	1.82E-08	31.81
rs78550103	A	G	-0.181	0.025	3.48E-13	53.01
rs78554005	T	G	-0.188	0.032	3.07E-09	35.13
rs78648104	T	C	-0.241	0.031	8.14E-15	60.20
rs786921	A	G	-0.115	0.018	8.63E-11	42.32
rs78809139	A	G	-0.228	0.029	2.58E-15	62.73
rs79043825	A	C	0.293	0.046	2.13E-10	40.33
rs79208229	T	G	0.213	0.033	6.53E-11	42.61
rs7926335	T	C	0.180	0.020	2.05E-20	85.59
rs79269465	T	G	0.221	0.040	2.12E-08	31.33
rs7927515	A	C	0.120	0.018	5.92E-11	42.86
rs7927966	T	C	-0.153	0.020	2.85E-14	57.67
rs7951348	T	C	0.153	0.017	1.00E-18	77.91
rs7959649	T	C	0.117	0.020	8.14E-09	33.32
rs7965392	A	G	0.112	0.018	4.16E-10	39.01
rs7967705	T	C	0.269	0.018	1.54E-51	229.06
rs7977406	A	G	-0.189	0.019	1.36E-23	99.79
rs79780963	T	C	-0.503	0.032	2.89E-54	240.73
rs798502	A	C	-0.109	0.019	9.65E-09	32.79
rs80095680	A	G	-0.157	0.020	2.81E-15	62.55
rs8014182	T	C	-0.194	0.026	3.94E-14	57.10
rs8046697	T	C	-0.129	0.018	6.10E-13	51.86
rs8068773	T	C	0.208	0.037	1.92E-08	31.52
rs8108717	A	G	0.132	0.018	1.39E-13	54.63
rs8128234	T	C	0.117	0.021	3.97E-08	30.28
rs8133022	A	G	-0.135	0.024	1.31E-08	32.25
rs818508	T	C	-0.120	0.021	1.46E-08	32.25
rs824522	A	G	0.169	0.021	9.52E-16	64.46
rs843078	A	G	-0.107	0.018	1.27E-09	36.96
rs848309	T	C	-0.138	0.017	2.75E-15	62.63
rs851966	A	G	-0.099	0.017	1.30E-08	32.31
rs870448	T	G	0.115	0.017	3.60E-11	43.83
rs871606	T	C	-0.184	0.028	8.56E-11	42.16
rs875106	A	G	-0.133	0.017	1.71E-14	59.01
rs880315	T	C	-0.258	0.019	1.99E-44	194.94
rs882624	T	C	-0.157	0.019	2.33E-17	72.11
rs896312	T	C	0.149	0.018	5.72E-16	65.66
rs907612	T	C	0.165	0.018	3.71E-19	80.22
rs908951	T	C	-0.198	0.018	7.73E-28	120.03
rs911547	A	G	-0.199	0.025	9.48E-16	64.58
rs9286351	A	G	-0.141	0.018	1.61E-15	63.64

rs929698	A	C	0.105	0.018	2.40E-09	35.46
rs932316	T	C	-0.240	0.022	1.14E-27	118.61
rs9326869	T	C	0.110	0.020	3.99E-08	30.03
rs9372082	A	G	-0.137	0.022	2.77E-10	39.98
rs9378123	A	G	-0.179	0.028	1.91E-10	40.58
rs9386780	A	G	0.098	0.017	1.46E-08	32.22
rs9399136	T	C	0.117	0.020	3.77E-09	34.86
rs9399437	A	G	0.125	0.020	2.09E-10	40.39
rs9401398	T	C	-0.107	0.018	1.41E-09	36.61
rs9406076	T	C	0.101	0.019	4.65E-08	29.81
rs9419374	A	G	0.116	0.019	3.44E-10	39.59
rs9431431	A	G	-0.134	0.019	1.71E-12	49.74
rs9456648	T	C	-0.117	0.019	2.76E-10	39.72
rs9472135	T	C	0.155	0.019	4.29E-16	66.38
rs9478282	T	C	-0.199	0.028	8.70E-13	51.08
rs9479509	A	G	-0.115	0.019	1.21E-09	36.76
rs9496614	T	C	0.131	0.021	1.63E-10	40.96
rs9508495	T	C	-0.194	0.020	1.34E-21	90.81
rs9526707	A	G	-0.122	0.019	6.59E-11	42.81
rs9532101	A	G	0.118	0.020	2.51E-09	35.40
rs9532243	A	C	0.134	0.017	1.09E-14	59.91
rs954767	A	C	-0.150	0.020	4.24E-14	57.09
rs9563529	T	G	0.122	0.022	1.38E-08	32.30
rs9609429	T	C	0.120	0.020	6.32E-10	38.06
rs962369	T	C	0.168	0.019	6.02E-19	79.39
rs9638084	A	G	0.115	0.018	8.51E-11	42.03
rs9652858	A	G	0.136	0.023	3.45E-09	35.02
rs9716164	T	C	0.101	0.018	7.22E-09	33.44
rs9787495	A	G	0.098	0.018	2.47E-08	31.00
rs983353	A	G	-0.109	0.019	8.52E-09	32.97
rs9870517	A	C	0.145	0.018	1.88E-16	67.48
rs9922726	A	C	0.098	0.018	3.17E-08	30.47
rs9932220	A	G	-0.159	0.021	3.76E-14	57.40
rs9932866	A	G	0.115	0.018	2.90E-10	39.77
rs9937801	T	C	0.155	0.017	4.81E-19	79.76
rs9939182	T	C	0.253	0.042	1.29E-09	36.78
rs994446	A	G	-0.221	0.021	6.01E-25	106.84
rs9967367	T	C	0.140	0.019	2.04E-13	53.80
