# Supplementary material for: Identifying modifiable risk factors of lung cancer: Indications from Mendelian randomization
Source: PLoS One. 2021 Oct 18;16(10):e0258498. doi: 10.1371/journal.pone.0258498 (PMC8523078; doi:10.1371/journal.pone.0258498)
Supplement: S10 Table — The SNP is the result of genetic variants; A1 is the effect allele; A2 is the other allele; beta is the effect size of A1 on the exposure; she is the standard error of beta; pval is the p-value of beta; F is the F statistics. (PDF) [file pone.0258498.s023.pdf]

S10 Table: Instrumental variables of SBP. SNP is the rsID of genetic variants; A1 is the effect allele; A2 is the other allele; beta is the effect size of A1 on the exposure; se is the standard error of beta; pval is the p value of beta; F is the F statistics.

SNP	A1	A2	beta	se	pval	F
rs1000423	T	C	0.414	0.035	6.50E-33	143.03
rs10008637	T	C	0.216	0.030	9.24E-13	51.01
rs10043077	T	C	-0.193	0.032	2.52E-09	35.52
rs10048404	T	C	-0.261	0.032	1.91E-16	67.63
rs10048760	T	G	-0.186	0.030	6.56E-10	38.27
rs1006545	T	G	0.685	0.048	3.50E-46	203.42
rs10069690	T	C	0.310	0.037	4.47E-17	70.49
rs10183431	T	C	-0.197	0.035	2.10E-08	31.47
rs10189186	A	G	0.189	0.030	3.91E-10	39.29
rs1019264	A	G	-0.187	0.031	2.03E-09	36.04
rs10205364	T	C	0.190	0.030	2.92E-10	39.72
rs10207726	T	C	-0.214	0.033	8.06E-11	42.13
rs10214535	A	G	0.181	0.033	3.35E-08	30.50
rs10224210	T	C	-0.383	0.034	1.60E-29	126.96
rs10254757	A	C	0.181	0.031	4.31E-09	34.46
rs10282122	T	C	-0.302	0.033	2.46E-20	85.29
rs1043069	T	G	0.234	0.031	5.26E-14	56.61
rs10433643	T	C	-0.232	0.042	3.83E-08	30.28
rs10437954	A	G	-0.410	0.053	1.60E-14	58.98
rs1044822	T	C	-0.248	0.042	5.16E-09	34.21
rs10460108	A	G	0.214	0.030	1.12E-12	50.59
rs10468291	A	C	-0.187	0.031	9.38E-10	37.51
rs1047891	A	C	-0.253	0.033	1.37E-14	59.40
rs10493891	T	C	-0.277	0.034	1.28E-16	68.37
rs10501410	A	G	0.412	0.061	1.10E-11	46.11
rs1052501	T	C	0.226	0.041	4.14E-08	30.14
rs10743348	T	C	0.292	0.034	1.06E-17	73.51
rs10746963	A	G	-0.218	0.039	2.05E-08	31.48
rs10761530	T	C	0.200	0.030	2.54E-11	44.62
rs1076485	T	C	0.339	0.046	1.19E-13	54.96
rs10776752	T	G	0.821	0.058	4.61E-46	203.21
rs10782230	A	G	0.211	0.030	2.91E-12	48.63
rs10820855	T	C	-0.190	0.033	1.05E-08	32.65
rs10832013	T	G	0.190	0.033	5.88E-09	33.97
rs10835148	A	G	0.192	0.031	8.69E-10	37.63
rs10841520	T	C	-0.249	0.038	4.97E-11	43.16
rs10875907	A	G	-0.716	0.122	4.28E-09	34.48
rs10895025	T	C	0.188	0.032	3.63E-09	34.81
rs10950289	A	G	0.234	0.041	1.52E-08	32.05

rs10980408	T	C	-0.761	0.083	3.83E-20	84.59
rs1098708	A	G	-0.197	0.030	8.21E-11	42.23
rs11031051	A	C	-0.224	0.033	7.73E-12	46.84
rs11044793	T	C	0.299	0.052	1.05E-08	32.71
rs11074093	T	C	-0.192	0.031	4.58E-10	38.77
rs11120093	T	C	-0.179	0.031	5.13E-09	34.07
rs111230791	T	C	-0.478	0.039	5.17E-35	152.81
rs1113480	A	C	0.215	0.034	1.50E-10	41.15
rs11145807	A	G	0.214	0.032	3.54E-11	43.96
rs11159091	A	G	0.198	0.030	6.79E-11	42.62
rs11168250	T	G	-0.279	0.036	5.40E-15	61.21
rs11191580	T	C	1.100	0.055	7.74E-89	399.64
rs11196549	A	G	0.688	0.078	1.58E-18	77.10
rs11197813	A	G	-0.182	0.033	3.53E-08	30.42
rs11210029	A	G	-0.203	0.031	8.92E-11	42.06
rs112260610	T	C	0.259	0.044	2.69E-09	35.40
rs11229115	A	G	-0.260	0.036	4.98E-13	52.08
rs11252324	T	G	-0.416	0.057	3.61E-13	52.81
rs11257593	A	G	0.176	0.031	8.55E-09	33.19
rs113006694	A	G	-0.381	0.066	7.76E-09	33.32
rs113086489	T	C	0.325	0.031	3.80E-26	112.00
rs1133400	A	G	-0.298	0.038	2.53E-15	62.60
rs114472600	T	C	-0.465	0.077	1.30E-09	36.82
rs1154214	T	G	-0.203	0.031	3.27E-11	44.05
rs11579440	T	C	0.267	0.043	3.24E-10	39.59
rs11592107	A	G	0.302	0.033	1.55E-20	86.05
rs11604310	T	C	-0.278	0.041	1.46E-11	45.69
rs11623535	A	G	0.210	0.034	1.02E-09	37.31
rs11629850	A	G	0.230	0.030	2.26E-14	58.24
rs11636952	T	C	0.531	0.033	4.22E-59	262.38
rs11638	A	G	-0.232	0.030	2.43E-14	58.29
rs11641374	A	C	-0.194	0.031	3.26E-10	39.54
rs11650511	T	C	0.243	0.031	1.07E-14	59.94
rs11655604	T	C	-0.203	0.033	1.09E-09	37.27
rs11685352	A	G	-0.227	0.039	6.14E-09	33.76
rs11689667	T	C	0.198	0.030	6.66E-11	42.55
rs11690717	T	G	-0.202	0.032	1.93E-10	40.61
rs117206641	T	C	0.315	0.050	2.66E-10	39.95
rs117464403	A	G	0.864	0.120	5.80E-13	51.93
rs1177701	A	G	0.172	0.030	1.54E-08	32.07
rs11862847	A	G	-0.168	0.030	2.29E-08	31.26
rs11868035	A	G	0.187	0.033	1.76E-08	31.79
rs11872627	T	C	-0.295	0.044	1.34E-11	45.66
rs11874246	T	C	0.286	0.033	3.23E-18	75.82

rs11876341	A	G	-0.213	0.033	1.82E-10	40.67
rs11960210	T	C	0.473	0.031	1.25E-51	228.08
rs11972067	A	G	-0.179	0.033	3.92E-08	30.12
rs11972506	T	C	-0.179	0.032	1.80E-08	31.74
rs11977526	A	G	-0.321	0.031	6.62E-25	106.05
rs11983200	A	G	-0.297	0.051	7.20E-09	33.54
rs11983783	T	C	0.176	0.031	9.48E-09	32.93
rs12042924	T	C	-0.181	0.030	2.62E-09	35.57
rs12050196	T	C	0.213	0.031	6.31E-12	47.12
rs12136922	A	G	0.203	0.030	2.69E-11	44.46
rs12146652	A	C	-0.358	0.031	9.00E-31	133.14
rs12147633	T	C	-0.538	0.090	2.60E-09	35.50
rs12149704	A	G	0.588	0.068	8.57E-18	73.82
rs12153395	A	G	-0.330	0.049	1.07E-11	46.19
rs1215469	A	C	-0.212	0.037	6.16E-09	33.86
rs12192157	T	C	0.185	0.032	4.43E-09	34.34
rs1229984	T	C	-0.599	0.090	2.42E-11	44.58
rs12363917	T	C	0.200	0.034	2.49E-09	35.61
rs12368199	A	G	-0.234	0.042	3.18E-08	30.55
rs12426261	A	G	0.378	0.031	2.31E-34	149.25
rs12446456	T	C	-0.300	0.030	2.97E-23	98.88
rs12454712	T	C	0.191	0.033	5.82E-09	33.84
rs12462548	A	G	0.332	0.040	7.20E-17	69.77
rs12509595	T	C	-0.837	0.033	2.55E-138	627.55
rs12511987	T	G	-0.233	0.040	5.39E-09	34.07
rs12528975	A	G	0.475	0.075	1.98E-10	40.45
rs12627651	A	G	0.350	0.034	1.02E-24	105.23
rs12630450	A	G	0.289	0.035	7.74E-17	69.57
rs12637573	A	G	-0.173	0.030	9.95E-09	32.85
rs12643599	A	G	0.313	0.031	1.23E-23	100.26
rs12656497	T	C	-0.638	0.031	7.14E-96	432.15
rs12657950	T	C	0.455	0.059	1.27E-14	59.47
rs12661036	T	C	-0.210	0.037	1.82E-08	31.65
rs12661604	A	G	0.178	0.031	9.87E-09	32.82
rs12664016	A	G	-0.316	0.057	2.83E-08	30.81
rs12668436	T	C	-0.215	0.035	7.88E-10	37.77
rs12692738	T	C	0.204	0.036	1.37E-08	32.32
rs12693095	T	G	-0.182	0.032	1.50E-08	32.11
rs12693982	T	C	0.258	0.031	7.49E-17	69.44
rs12731646	T	C	-0.189	0.031	7.21E-10	37.90
rs1275988	T	C	-0.541	0.031	4.42E-69	308.53
rs12901664	T	C	0.182	0.032	9.60E-09	32.86
rs12906962	T	C	-0.265	0.033	3.28E-16	66.64

rs1290784	T	C	0.412	0.030	2.97E-42	185.25
rs1290933	A	C	-0.285	0.033	3.17E-18	75.80
rs1293965	A	G	-0.200	0.035	9.83E-09	32.93
rs12971628	T	G	-0.254	0.032	4.17E-15	61.64
rs12982630	A	C	-0.251	0.035	1.38E-12	50.19
rs13015703	T	C	0.208	0.034	1.45E-09	36.67
rs13016772	T	C	0.252	0.036	1.23E-12	50.47
rs13095509	T	C	0.859	0.107	7.98E-16	64.86
rs13107261	A	G	-0.178	0.031	1.57E-08	32.06
rs13107325	T	C	-0.909	0.059	4.22E-53	235.56
rs13128814	A	G	-0.217	0.031	1.24E-12	50.48
rs13143677	A	G	0.224	0.033	1.82E-11	45.13
rs13149209	T	C	0.281	0.037	1.97E-14	58.62
rs13165038	T	C	-0.192	0.032	3.32E-09	35.01
rs13179413	T	C	0.224	0.035	1.08E-10	41.60
rs13203975	A	G	0.279	0.048	4.46E-09	34.43
rs13207962	A	G	-0.250	0.036	1.78E-12	49.75
rs13227860	A	G	0.222	0.035	1.44E-10	41.04
rs13253358	T	C	0.213	0.033	1.13E-10	41.54
rs13290326	T	C	-0.212	0.030	1.52E-12	50.03
rs1331012	T	G	0.204	0.034	1.49E-09	36.53
rs1332813	T	C	0.220	0.031	2.32E-12	49.22
rs13358657	A	G	-0.388	0.045	2.95E-18	76.02
rs13383272	A	G	-0.172	0.031	1.74E-08	31.80
rs13412750	A	G	-0.289	0.034	2.33E-17	71.78
rs1347345	A	G	-0.181	0.031	6.92E-09	33.51
rs1368298	A	G	0.309	0.031	4.76E-24	102.31
rs1375564	T	C	0.258	0.032	2.84E-16	67.03
rs137923903	T	C	-1.280	0.146	2.25E-18	76.49
rs1379617	T	C	0.217	0.036	1.54E-09	36.44
rs1408945	T	G	-0.320	0.030	8.33E-26	110.53
rs141212865	A	C	0.301	0.039	7.68E-15	60.49
rs1421203	T	C	-0.350	0.040	7.86E-19	78.47
rs142449193	T	C	-0.455	0.074	7.86E-10	37.79
rs1432564	T	C	-0.175	0.030	7.52E-09	33.29
rs143285018	A	G	0.865	0.124	2.88E-12	48.79
rs1433121	T	C	-0.228	0.033	2.66E-12	48.91
rs143333049	A	G	-0.542	0.087	4.13E-10	39.05
rs1436138	A	G	0.312	0.032	4.73E-23	98.04
rs144311783	T	C	0.493	0.086	8.13E-09	33.21
rs145042302	A	G	-0.589	0.097	1.39E-09	36.67
rs145211473	A	G	0.751	0.112	1.86E-11	45.08
rs1465091	A	C	-0.361	0.039	1.75E-20	85.88
rs1474698	T	C	-0.177	0.031	6.73E-09	33.56

rs148140538	T	C	-0.325	0.056	7.39E-09	33.48
rs1486236	A	C	-0.234	0.032	2.96E-13	53.09
rs149487184	T	C	-0.634	0.105	1.46E-09	36.59
rs151117229	T	C	0.612	0.081	3.14E-14	57.70
rs1514086	A	G	-0.433	0.059	2.95E-13	53.24
rs1536608	T	G	0.194	0.030	1.57E-10	41.04
rs1551355	T	C	0.210	0.036	3.89E-09	34.73
rs1565440	A	G	0.175	0.031	1.94E-08	31.52
rs157079	T	C	0.924	0.149	5.83E-10	38.37
rs1575290	T	C	0.197	0.030	5.59E-11	42.97
rs1624823	A	G	0.337	0.031	4.26E-27	115.99
rs1630736	T	C	-0.171	0.031	3.52E-08	30.48
rs1657880	T	C	-0.287	0.050	7.44E-09	33.39
rs167479	T	G	-0.564	0.033	7.21E-67	297.69
rs1688790	A	G	-0.170	0.030	1.87E-08	31.72
rs16939357	T	C	0.311	0.042	1.37E-13	54.71
rs17010957	T	C	-0.534	0.043	1.78E-35	154.22
rs17035181	T	G	0.307	0.043	7.61E-13	51.34
rs17115145	T	C	0.178	0.031	7.39E-09	33.43
rs17171710	T	C	-0.409	0.049	1.13E-16	68.69
rs17245822	A	C	-0.190	0.031	1.15E-09	37.05
rs17249754	A	G	-0.845	0.040	1.25E-97	439.23
rs17257081	A	G	0.227	0.039	6.35E-09	33.65
rs17376426	T	C	0.676	0.087	9.87E-15	59.87
rs17473424	A	G	0.531	0.050	1.25E-26	114.24
rs17608766	T	C	-0.690	0.043	2.48E-57	254.16
rs17638167	T	C	-0.452	0.074	1.07E-09	37.19
rs17730281	A	G	-0.200	0.036	2.38E-08	31.18
rs17760259	T	C	-0.265	0.030	2.25E-18	76.22
rs17778035	T	C	-0.241	0.044	3.94E-08	30.20
rs17812022	T	C	-0.361	0.053	5.65E-12	47.36
rs17826049	T	G	-0.621	0.077	1.09E-15	64.33
rs1790998	A	C	-0.209	0.031	9.10E-12	46.52
rs179972	T	C	0.183	0.031	5.46E-09	33.93
rs1808192	A	G	-0.184	0.032	1.38E-08	32.32
rs1815614	A	G	0.235	0.031	3.73E-14	57.37
rs182050989	T	C	0.580	0.088	3.87E-11	43.72
rs1848510	A	G	0.184	0.032	5.72E-09	33.87
rs1848994	A	G	0.201	0.033	1.79E-09	36.29
rs1871190	T	G	0.195	0.032	1.66E-09	36.37
rs1879057	T	C	-0.325	0.038	1.67E-17	72.43
rs1882961	T	C	0.244	0.033	6.69E-14	56.16
rs1888693	A	G	0.386	0.032	4.69E-34	148.12
rs1891392	T	C	0.228	0.033	9.02E-12	46.44

rs1891730	T	C	-0.181	0.031	7.74E-09	33.30
rs1906672	A	G	0.297	0.036	1.20E-16	68.64
rs1921151	A	G	0.300	0.038	1.27E-15	64.00
rs1957563	T	C	0.363	0.034	2.32E-26	112.60
rs1960024	A	G	0.187	0.031	2.00E-09	36.04
rs1984195	A	G	0.241	0.030	1.77E-15	63.21
rs1998107	A	G	-0.190	0.030	2.23E-10	40.28
rs2007361	A	G	-0.194	0.032	8.69E-10	37.61
rs2009598	A	G	0.222	0.031	4.61E-13	52.34
rs2014408	T	C	0.517	0.037	1.26E-43	192.04
rs2032451	T	G	0.543	0.042	1.56E-37	163.71
rs2054479	T	G	-0.265	0.034	3.02E-15	62.34
rs2060664	T	C	0.216	0.035	4.06E-10	39.20
rs2065498	T	G	-0.293	0.040	3.36E-13	53.00
rs2071287	T	C	0.299	0.032	8.90E-21	87.25
rs2093324	A	G	-0.180	0.031	9.75E-09	32.90
rs2098839	T	C	0.199	0.032	4.92E-10	38.72
rs2105388	A	C	0.178	0.031	5.70E-09	34.02
rs2107595	A	G	0.418	0.042	7.37E-24	101.26
rs2112453	A	G	-0.188	0.034	3.69E-08	30.27
rs2126474	T	G	-0.260	0.031	1.87E-17	72.25
rs2131957	A	C	-0.206	0.031	1.98E-11	44.94
rs2156805	A	G	0.178	0.030	4.64E-09	34.32
rs2161967	T	G	0.284	0.031	2.87E-20	85.34
rs2177843	T	C	0.439	0.043	2.80E-24	103.46
rs2206815	A	C	-0.327	0.030	5.52E-27	115.56
rs2210893	T	C	-0.190	0.033	4.73E-09	34.21
rs2236295	T	G	-0.303	0.031	1.05E-22	96.03
rs2238787	A	G	0.255	0.033	1.45E-14	59.09
rs2239046	A	G	0.208	0.032	9.58E-11	41.81
rs2240365	T	C	0.419	0.069	1.33E-09	36.82
rs2246754	T	C	0.250	0.037	1.17E-11	46.08
rs2249105	A	G	0.293	0.031	7.63E-21	87.45
rs2273171	T	C	-0.167	0.030	2.64E-08	30.91
rs227426	T	G	0.186	0.030	8.59E-10	37.68
rs2289124	A	G	-0.308	0.042	1.14E-13	55.08
rs2290263	A	G	0.268	0.035	3.11E-14	57.77
rs2291434	T	G	-0.262	0.030	5.10E-18	74.88
rs2297387	A	G	-0.173	0.032	4.56E-08	29.94
rs2300481	T	C	0.198	0.031	1.56E-10	40.84
rs2302263	T	C	0.326	0.054	1.07E-09	37.22
rs2306363	T	G	-0.436	0.038	5.24E-31	134.34
rs2327429	T	C	0.200	0.034	3.16E-09	35.01
rs2354862	A	C	0.251	0.032	2.42E-15	62.54

rs2357945	A	G	-0.307	0.048	1.71E-10	40.84
rs2384063	T	C	0.327	0.036	6.33E-20	83.69
rs2392929	T	G	-0.751	0.038	1.96E-87	392.33
rs2395622	T	C	-0.227	0.039	7.17E-09	33.42
rs2417189	T	G	0.243	0.031	1.03E-14	59.84
rs2419077	T	G	0.197	0.034	1.07E-08	32.66
rs246973	T	C	0.248	0.034	1.45E-13	54.76
rs2470004	T	C	-0.345	0.039	1.28E-18	77.64
rs2480171	T	C	0.291	0.047	4.69E-10	38.80
rs2493291	T	C	0.421	0.044	1.85E-21	90.64
rs2498323	A	G	0.317	0.052	8.52E-10	37.62
rs2549732	A	G	-0.455	0.059	1.27E-14	59.39
rs256741	A	C	0.168	0.031	4.50E-08	29.96
rs2596498	T	C	-0.233	0.034	4.90E-12	47.80
rs2608882	A	G	-0.252	0.036	3.43E-12	48.54
rs2610990	A	G	-0.290	0.034	2.86E-17	71.63
rs2611774	A	G	0.219	0.032	6.85E-12	46.97
rs2613765	A	G	-0.235	0.030	5.32E-15	61.11
rs2627316	A	G	-0.333	0.030	2.79E-28	121.36
rs262986	A	G	-0.237	0.031	7.67E-15	60.43
rs2643826	T	C	0.447	0.031	1.74E-48	213.68
rs2652812	T	C	-0.252	0.035	1.03E-12	50.80
rs267517	A	G	-0.261	0.031	7.18E-17	69.53
rs2693560	A	G	-0.206	0.032	6.99E-11	42.50
rs2724377	A	G	0.194	0.030	1.29E-10	41.45
rs2744133	A	G	0.288	0.034	1.33E-17	72.98
rs2745599	A	G	0.216	0.032	8.96E-12	46.60
rs2753960	T	G	0.447	0.031	2.66E-47	208.89
rs2801008	T	G	-0.188	0.032	7.37E-09	33.53
rs2807337	T	C	0.186	0.031	2.78E-09	35.24
rs2815063	A	C	0.276	0.046	1.76E-09	36.18
rs2833834	A	C	0.218	0.034	1.22E-10	41.48
rs28558491	T	C	-0.211	0.034	7.54E-10	37.99
rs28572127	A	C	-0.178	0.032	3.51E-08	30.40
rs28572357	A	C	-0.273	0.031	6.34E-19	78.74
rs28578714	T	C	0.207	0.033	2.53E-10	39.92
rs28621435	A	G	-0.298	0.048	6.47E-10	38.12
rs2869668	T	C	0.174	0.030	6.92E-09	33.53
rs28708888	A	C	-0.207	0.032	1.50E-10	41.17
rs2872717	T	C	-0.265	0.036	1.01E-13	55.20
rs28730765	T	C	0.815	0.141	7.78E-09	33.35
rs28866311	T	G	-0.276	0.030	5.45E-20	83.64
rs2892987	A	G	0.322	0.054	2.95E-09	35.23
rs2913920	T	C	0.242	0.036	1.62E-11	45.37

rs2943648	A	G	-0.256	0.031	2.61E-16	67.00
rs2957468	A	G	0.247	0.032	1.59E-14	58.98
rs296797	T	C	0.216	0.031	2.16E-12	49.23
rs2978098	A	C	0.223	0.031	3.78E-13	52.62
rs2978456	T	C	-0.180	0.031	8.95E-09	33.00
rs2999159	A	G	-0.435	0.040	1.96E-27	117.89
rs3058639	T	C	-0.189	0.034	1.68E-08	31.80
rs307359	A	G	-0.379	0.065	5.18E-09	34.14
rs3098186	T	C	-0.242	0.030	1.41E-15	63.89
rs3128334	A	G	0.207	0.032	1.41E-10	41.07
rs314779	T	G	-0.212	0.033	1.79E-10	40.66
rs33996239	T	C	-0.366	0.066	3.39E-08	30.48
rs34025993	A	G	0.223	0.031	4.71E-13	52.42
rs34072724	A	G	-0.242	0.030	1.37E-15	63.89
rs34130368	T	G	-0.302	0.050	1.28E-09	36.83
rs342130	A	G	0.189	0.031	1.34E-09	36.78
rs34297584	A	G	0.795	0.113	2.13E-12	49.38
rs34475503	A	G	-0.379	0.054	1.31E-12	50.26
rs34518929	A	G	-0.220	0.035	1.79E-10	40.66
rs34587622	T	C	-0.329	0.051	1.67E-10	40.90
rs34727427	T	C	-0.235	0.032	4.02E-13	52.74
rs34869093	A	G	-0.220	0.031	1.84E-12	49.59
rs34905952	A	G	0.287	0.041	3.00E-12	48.73
rs34941092	A	G	-0.323	0.043	3.23E-14	57.58
rs34983854	A	G	-0.206	0.031	2.06E-11	44.85
rs34991912	T	C	0.311	0.031	3.98E-24	102.69
rs35213536	T	G	0.294	0.036	1.94E-16	67.68
rs35413927	A	G	-0.300	0.033	5.25E-20	83.77
rs35444	A	G	0.437	0.031	3.47E-45	198.54
rs35519679	A	G	0.254	0.036	1.15E-12	50.46
rs35680304	T	C	0.269	0.031	3.76E-18	75.52
rs35754956	A	G	-0.290	0.048	1.08E-09	37.12
rs35783704	A	G	-0.462	0.051	8.81E-20	83.00
rs360151	A	G	0.327	0.052	2.16E-10	40.27
rs36027301	T	C	-0.422	0.067	2.99E-10	39.70
rs365990	A	G	0.225	0.031	5.95E-13	52.01
rs366590	A	G	0.219	0.033	2.10E-11	44.92
rs3729931	A	G	-0.182	0.031	6.29E-09	33.71
rs3735533	T	C	-0.910	0.058	5.29E-56	248.73
rs3743157	A	C	0.293	0.040	4.20E-13	52.60
rs3754944	A	C	0.177	0.031	9.30E-09	32.95
rs3757387	T	C	0.190	0.030	4.01E-10	39.23
rs3763917	A	C	0.212	0.038	1.82E-08	31.73
rs3772219	A	C	0.273	0.032	3.10E-17	71.15

rs3807925	A	G	-0.186	0.032	5.39E-09	33.96
rs3808356	T	C	0.197	0.034	4.91E-09	34.31
rs3821843	A	G	0.337	0.034	6.56E-24	101.38
rs3827676	T	C	0.175	0.031	1.17E-08	32.42
rs3843712	T	C	-0.340	0.051	1.91E-11	45.12
rs3847320	A	G	-0.191	0.034	2.82E-08	30.83
rs3860770	A	G	-0.266	0.033	1.20E-15	63.95
rs3894010	T	G	-0.201	0.031	5.12E-11	42.99
rs3910508	A	G	0.261	0.032	1.38E-16	68.38
rs3915499	A	G	-0.207	0.032	1.36E-10	41.13
rs3918226	T	C	0.664	0.058	8.46E-31	133.35
rs3923060	T	C	0.192	0.035	3.64E-08	30.34
rs3980686	T	G	-0.500	0.049	1.03E-24	105.33
rs41267086	A	G	0.505	0.069	3.41E-13	52.99
rs4129585	A	C	0.186	0.031	1.03E-09	37.27
rs4143175	T	C	0.219	0.035	5.10E-10	38.60
rs42377	A	G	-0.315	0.033	1.69E-21	90.74
rs4245165	T	C	-0.225	0.036	2.39E-10	40.09
rs4245599	A	G	-0.179	0.031	4.04E-09	34.60
rs4245930	A	G	-0.202	0.031	1.04E-10	41.73
rs4286632	A	G	0.211	0.034	7.64E-10	37.84
rs4319878	T	C	0.169	0.031	3.77E-08	30.25
rs4320727	A	G	0.234	0.031	9.89E-14	55.44
rs4440615	A	G	-0.220	0.031	1.87E-12	49.77
rs4456714	T	C	-0.191	0.035	3.24E-08	30.54
rs4480845	T	C	0.316	0.032	1.85E-23	99.75
rs4496141	T	G	0.180	0.030	2.28E-09	35.64
rs4511593	T	C	-0.288	0.032	1.28E-19	82.08
rs4553000	T	C	-0.204	0.030	1.09E-11	46.01
rs4573493	T	C	0.167	0.030	3.86E-08	30.30
rs4577304	T	C	-0.177	0.030	4.99E-09	34.23
rs4582532	A	G	-0.200	0.030	3.64E-11	43.70
rs4595370	A	G	-0.209	0.033	1.73E-10	40.68
rs4598218	T	C	0.191	0.031	1.00E-09	37.28
rs464605	T	C	0.208	0.035	2.08E-09	35.83
rs4651224	T	C	0.199	0.031	9.00E-11	42.12
rs4696128	T	C	-0.170	0.031	2.78E-08	30.83
rs4704514	T	C	0.273	0.034	3.92E-16	66.16
rs4738141	A	G	-0.193	0.035	3.12E-08	30.55
rs4754196	A	G	-0.349	0.030	1.46E-30	132.36
rs4760701	A	G	-0.293	0.037	1.95E-15	63.14
rs4775769	T	G	-0.416	0.052	7.76E-16	64.81
rs4793069	A	G	0.374	0.066	1.67E-08	31.86
rs4812536	A	G	0.271	0.032	4.09E-17	70.60

rs4818833	A	G	-0.226	0.031	2.39E-13	53.68
rs4864421	T	C	-0.217	0.031	2.14E-12	49.50
rs4873492	T	C	0.343	0.040	1.61E-17	72.48
rs4875958	A	G	0.226	0.034	1.85E-11	45.08
rs4888408	A	G	0.365	0.031	1.42E-32	141.59
rs4908348	T	G	0.237	0.033	8.07E-13	51.40
rs4925159	A	G	0.217	0.031	9.66E-13	50.81
rs4926923	T	C	0.320	0.054	2.56E-09	35.49
rs4932373	A	C	-0.635	0.033	2.49E-83	374.80
rs4948643	T	C	0.226	0.034	2.40E-11	44.63
rs4952668	A	G	-0.248	0.031	2.60E-15	62.58
rs4957026	A	G	0.198	0.032	8.12E-10	37.65
rs4961293	T	C	0.227	0.030	7.35E-14	56.03
rs4963661	T	C	-0.252	0.035	5.17E-13	52.01
rs4965529	A	C	-0.273	0.040	1.27E-11	45.82
rs4980515	T	C	0.225	0.030	9.73E-14	55.29
rs509833	A	G	0.329	0.044	7.08E-14	55.91
rs519537	A	G	0.197	0.030	5.20E-11	42.97
rs525271	T	C	0.234	0.033	1.67E-12	49.81
rs55678414	T	G	-0.689	0.063	1.56E-27	118.07
rs55732192	T	G	-0.336	0.052	1.15E-10	41.54
rs557526	A	G	-0.206	0.036	6.53E-09	33.61
rs55752997	A	C	0.387	0.060	1.01E-10	41.78
rs55924432	T	C	0.265	0.032	5.70E-17	69.94
rs55938136	A	G	0.252	0.040	1.71E-10	40.83
rs55944332	A	G	-0.261	0.036	1.79E-13	54.18
rs56085433	A	G	-0.251	0.044	8.21E-09	33.29
rs56213443	A	C	-0.174	0.031	1.39E-08	32.15
rs56288724	A	G	-0.218	0.031	2.01E-12	49.36
rs56334134	A	G	0.173	0.032	3.69E-08	30.27
rs56388530	T	C	0.416	0.036	2.84E-31	135.29
rs56407827	T	C	0.360	0.034	2.78E-26	112.30
rs56719405	A	G	0.358	0.065	3.88E-08	30.17
rs569550	T	G	-0.577	0.032	1.33E-73	328.66
rs571689	T	C	0.228	0.030	6.77E-14	56.25
rs573455	A	G	0.199	0.030	4.77E-11	43.31
rs5742643	T	C	-0.223	0.035	1.53E-10	40.94
rs57946343	T	C	0.716	0.043	2.10E-63	282.49
rs59296210	T	C	0.592	0.089	2.63E-11	44.43
rs59400568	A	G	0.224	0.038	2.88E-09	35.27
rs59980837	T	G	1.100	0.116	3.32E-21	89.41
rs60191654	A	G	-0.238	0.039	5.88E-10	38.28
rs6021247	A	G	0.233	0.030	9.80E-15	59.82
rs6031431	A	G	-0.262	0.030	7.05E-18	74.11

rs6039211	A	G	0.283	0.031	1.94E-19	81.52
rs6040316	T	C	-0.986	0.147	1.85E-11	45.10
rs604723	T	C	-0.655	0.034	2.55E-83	373.32
rs6054139	A	G	0.209	0.031	8.23E-12	46.83
rs6058088	T	G	0.283	0.042	1.14E-11	46.12
rs6062324	A	G	-0.329	0.036	1.18E-19	82.34
rs60672471	T	C	0.278	0.049	1.49E-08	32.07
rs606950	A	G	0.270	0.031	3.23E-18	75.59
rs6078093	A	G	-0.185	0.030	1.20E-09	36.99
rs6090907	A	G	-0.385	0.043	1.29E-19	82.23
rs60991988	T	G	0.379	0.050	2.82E-14	57.89
rs6108787	T	G	-0.427	0.030	5.38E-46	202.97
rs613872	T	G	-0.224	0.040	1.67E-08	31.88
rs6141766	A	G	-0.314	0.042	9.94E-14	55.49
rs61762319	A	G	0.571	0.100	1.28E-08	32.38
rs61772592	A	G	-0.318	0.046	2.86E-12	48.88
rs61798313	A	G	-0.513	0.079	6.60E-11	42.62
rs61929307	T	G	-0.190	0.032	1.79E-09	36.19
rs61942628	T	C	0.328	0.057	9.95E-09	32.84
rs61948066	T	G	0.281	0.047	2.07E-09	35.92
rs62039771	T	G	0.332	0.051	6.62E-11	42.54
rs62059712	T	C	0.494	0.058	9.88E-18	73.55
rs62169534	T	C	0.190	0.030	3.74E-10	39.36
rs62301873	A	G	-0.300	0.050	1.60E-09	36.34
rs62309747	A	G	-0.224	0.030	1.59E-13	54.49
rs62421489	A	G	0.397	0.055	8.34E-13	51.25
rs62523863	A	G	0.269	0.037	2.87E-13	53.39
rs6271	T	C	-0.555	0.061	1.18E-19	82.42
rs6416749	T	C	0.231	0.034	9.68E-12	46.27
rs6438857	T	C	0.274	0.031	3.13E-19	80.47
rs6452769	A	G	-0.314	0.038	7.82E-17	69.50
rs6466878	T	C	0.196	0.032	1.54E-09	36.52
rs6490019	A	G	-0.290	0.031	6.61E-21	87.90
rs6497759	A	G	-0.327	0.038	8.79E-18	73.75
rs6504213	T	C	-0.298	0.031	1.25E-21	91.35
rs6538211	T	C	0.217	0.030	4.59E-13	52.51
rs6539467	A	G	0.265	0.040	5.57E-11	43.03
rs6540125	T	G	0.204	0.032	1.21E-10	41.49
rs6557155	T	G	0.191	0.031	1.14E-09	37.08
rs6562778	A	G	0.178	0.030	4.96E-09	34.28
rs6565174	A	C	-0.288	0.048	2.57E-09	35.46
rs6567160	T	C	0.224	0.036	3.33E-10	39.44
rs6570530	T	C	0.221	0.031	6.04E-13	51.97
rs658780	T	G	-0.203	0.035	5.29E-09	34.16

rs6723509	T	C	0.253	0.044	7.61E-09	33.39
rs6734118	A	C	-0.323	0.037	1.42E-18	77.32
rs6737318	A	G	0.235	0.036	1.13E-10	41.61
rs6737690	A	C	-0.233	0.035	1.69E-11	45.42
rs6739913	A	G	-0.182	0.033	4.63E-08	29.82
rs6771917	T	C	-0.379	0.036	1.39E-26	114.16
rs67720684	A	C	0.194	0.035	3.85E-08	30.30
rs6785824	A	G	0.167	0.030	3.30E-08	30.54
rs67885470	T	C	-0.209	0.038	4.12E-08	30.16
rs6788984	A	G	0.300	0.043	3.81E-12	48.19
rs6806529	A	C	0.177	0.031	1.03E-08	32.74
rs68085857	T	C	0.274	0.036	1.68E-14	58.91
rs6823767	T	C	-0.213	0.034	4.40E-10	38.98
rs687914	T	G	0.248	0.035	1.90E-12	49.64
rs6884092	A	C	0.278	0.051	3.76E-08	30.23
rs6892983	A	C	0.343	0.031	7.11E-29	124.61
rs6894014	T	G	0.275	0.030	1.09E-19	82.31
rs6905288	A	G	0.190	0.031	1.09E-09	37.16
rs6911827	T	C	0.238	0.031	7.96E-15	60.39
rs6921291	T	C	0.358	0.039	1.58E-20	86.22
rs6957161	A	G	0.206	0.035	2.20E-09	35.79
rs6959688	A	G	-0.234	0.031	4.22E-14	57.17
rs6963105	A	G	-0.189	0.032	3.83E-09	34.74
rs698748	A	G	0.187	0.033	8.90E-09	33.14
rs699	A	G	-0.375	0.031	5.59E-34	148.08
rs7009170	T	C	-0.215	0.032	3.01E-11	44.07
rs7012866	T	G	-0.233	0.030	1.21E-14	59.66
rs7023828	T	C	-0.260	0.031	2.17E-17	71.92
rs702395	T	C	0.232	0.031	3.24E-14	57.76
rs704	A	G	-0.169	0.030	1.94E-08	31.67
rs7069881	T	C	-0.181	0.033	2.90E-08	30.69
rs708117	A	G	0.287	0.030	1.59E-21	90.56
rs709668	A	G	-0.294	0.038	5.96E-15	60.94
rs7100912	T	G	0.241	0.032	3.85E-14	57.12
rs7106914	T	C	-0.217	0.030	6.95E-13	51.73
rs7107356	A	G	-0.460	0.030	1.63E-52	233.35
rs7125196	T	C	0.442	0.047	7.31E-21	87.77
rs7134677	T	C	-0.385	0.033	4.46E-31	134.55
rs71355297	T	C	0.199	0.036	4.09E-08	30.14
rs7154723	A	G	0.253	0.031	2.72E-16	67.04
rs7156903	T	G	-0.195	0.031	5.36E-10	38.61
rs7165200	A	G	0.201	0.030	2.87E-11	44.34
rs7186298	T	C	-0.232	0.030	1.88E-14	58.76
rs7196195	T	G	-0.221	0.030	3.35E-13	52.94

rs7198817	A	C	-0.182	0.031	5.99E-09	33.96
rs7204624	A	G	0.202	0.035	1.18E-08	32.59
rs7213273	A	G	-0.400	0.032	6.24E-37	161.25
rs7218708	A	G	-0.178	0.030	4.38E-09	34.55
rs7246865	A	G	0.276	0.034	1.05E-15	64.51
rs7250497	A	G	0.205	0.032	1.16E-10	41.40
rs7255933	A	G	0.231	0.035	2.44E-11	44.68
rs7256564	A	G	0.196	0.032	1.53E-09	36.41
rs72632819	T	G	0.186	0.033	2.40E-08	31.22
rs72654647	A	G	0.211	0.036	2.64E-09	35.36
rs72661887	T	C	0.246	0.031	3.88E-15	61.97
rs72683923	T	C	0.959	0.110	3.08E-18	75.82
rs72688070	T	C	-0.271	0.041	2.82E-11	44.39
rs72834453	T	G	-0.325	0.047	2.95E-12	48.73
rs72837033	A	G	0.235	0.032	1.89E-13	53.98
rs72847885	A	G	0.241	0.032	3.08E-14	57.58
rs72931748	A	G	0.397	0.053	6.40E-14	56.24
rs73046792	A	G	-0.355	0.043	7.23E-17	69.60
rs73049928	A	G	-0.238	0.039	1.20E-09	36.92
rs7306710	T	C	-0.243	0.030	1.03E-15	64.26
rs73075659	A	G	0.396	0.032	5.52E-35	152.34
rs73088122	T	C	-0.256	0.044	4.83E-09	34.24
rs7315980	A	G	0.480	0.056	7.35E-18	74.12
rs73164066	T	C	-0.217	0.033	3.18E-11	44.12
rs73187288	A	C	-0.277	0.048	1.04E-08	32.80
rs7331680	T	G	0.410	0.042	3.35E-22	93.99
rs733329	T	C	0.302	0.045	3.09E-11	44.19
rs7340705	T	C	-0.243	0.032	4.87E-14	56.72
rs73727605	A	G	0.362	0.062	6.60E-09	33.69
rs7394746	T	C	0.228	0.031	9.08E-14	55.69
rs74026308	T	G	0.244	0.041	3.28E-09	35.02
rs74048200	A	G	-0.422	0.057	1.90E-13	54.05
rs7406910	T	C	-0.435	0.053	3.39E-16	66.52
rs7407	T	C	-0.330	0.032	1.33E-25	109.32
rs7412	T	C	-0.434	0.057	4.04E-14	57.14
rs74179970	A	G	0.408	0.057	6.83E-13	51.57
rs7439567	T	C	0.254	0.031	2.31E-16	67.41
rs7460241	A	G	-0.425	0.075	1.34E-08	32.30
rs749109	A	G	-0.222	0.037	2.83E-09	35.36
rs7500448	A	G	0.223	0.035	1.98E-10	40.49
rs75016974	T	C	-0.251	0.044	1.05E-08	32.77
rs75088623	A	G	0.199	0.034	5.10E-09	34.09
rs7514579	A	C	0.224	0.036	5.45E-10	38.61
rs752229	A	G	-0.404	0.046	1.60E-18	77.21

rs75497489	T	C	0.242	0.040	1.00E-09	37.41
rs7553381	A	C	-0.196	0.036	3.76E-08	30.23
rs7562	T	C	0.231	0.031	3.26E-14	57.51
rs75672964	T	C	0.589	0.084	2.35E-12	49.20
rs7578940	T	G	0.184	0.030	1.10E-09	37.08
rs75961402	A	G	0.266	0.042	1.95E-10	40.47
rs7600439	A	G	-0.184	0.030	1.19E-09	36.92
rs7606205	A	C	-0.184	0.033	3.06E-08	30.63
rs7615099	A	G	0.189	0.032	3.90E-09	34.70
rs7630377	T	C	0.174	0.030	7.30E-09	33.53
rs76452347	T	C	-0.297	0.040	7.13E-14	56.12
rs76719272	T	C	-0.274	0.046	2.97E-09	35.27
rs767717	T	C	0.220	0.031	1.92E-12	49.68
rs7683728	T	C	-0.365	0.030	2.43E-33	144.47
rs7703560	A	G	-0.225	0.033	1.51E-11	45.49
rs77151571	T	C	0.574	0.089	1.24E-10	41.37
rs7725413	T	C	-0.199	0.036	3.07E-08	30.57
rs7734385	A	G	-0.181	0.031	3.83E-09	34.68
rs77375686	A	G	-0.347	0.049	8.38E-13	51.10
rs77413490	T	G	0.449	0.076	4.27E-09	34.52
rs7744319	T	C	-0.175	0.031	2.23E-08	31.28
rs7751856	A	C	0.197	0.033	1.68E-09	36.41
rs7763294	T	G	-0.200	0.032	6.39E-10	38.26
rs7763558	A	G	0.336	0.032	1.17E-25	109.76
rs7765526	A	G	0.201	0.031	5.88E-11	42.87
rs778124	A	G	0.297	0.031	1.45E-21	90.89
rs77924615	A	G	-0.408	0.039	1.12E-25	109.50
rs7821832	T	G	0.422	0.035	6.67E-34	147.19
rs78474310	A	G	-0.470	0.073	1.51E-10	40.98
rs78550103	A	G	-0.284	0.043	4.45E-11	43.48
rs78648104	T	C	-0.429	0.054	2.37E-15	62.79
rs786923	T	C	-0.308	0.031	2.83E-23	98.84
rs7874646	T	C	-0.206	0.038	4.25E-08	29.96
rs79119657	A	G	-0.232	0.039	2.29E-09	35.78
rs7912283	A	G	-0.214	0.032	2.94E-11	44.33
rs79208229	T	G	0.381	0.057	1.99E-11	45.06
rs7927515	A	C	0.227	0.032	1.05E-12	50.68
rs7944927	T	C	0.224	0.039	1.23E-08	32.51
rs79539362	T	C	0.400	0.050	2.09E-15	63.08
rs7963801	T	C	-0.236	0.031	2.87E-14	57.68
rs79682447	A	G	0.501	0.081	6.02E-10	38.32
rs7976260	T	C	-0.708	0.105	1.31E-11	45.75
rs7977406	A	G	-0.346	0.033	4.13E-26	111.70
rs79780963	T	C	-1.097	0.057	1.21E-83	375.37

rs79802331	T	G	0.338	0.054	3.84E-10	39.13
rs79917357	A	G	0.298	0.040	1.40E-13	54.68
rs79932633	A	C	-0.274	0.049	3.10E-08	30.67
rs8003103	A	G	-0.176	0.032	3.60E-08	30.27
rs80045342	A	G	-0.334	0.060	2.12E-08	31.42
rs80100774	T	G	0.370	0.061	1.43E-09	36.61
rs8014182	T	C	-0.334	0.044	5.23E-14	56.55
rs8077276	A	G	-0.295	0.031	5.15E-21	88.20
rs813412	T	C	-0.210	0.035	1.87E-09	36.14
rs8142376	T	C	0.168	0.030	2.20E-08	31.21
rs839755	A	C	-0.267	0.031	5.41E-18	74.87
rs848309	T	C	-0.246	0.030	5.98E-16	65.54
rs848445	T	C	-0.203	0.034	2.28E-09	35.68
rs849138	A	G	-0.189	0.030	3.99E-10	39.03
rs863930	T	G	-0.189	0.030	3.98E-10	39.00
rs869396	A	C	-0.212	0.031	4.12E-12	48.09
rs871004	A	G	0.234	0.032	1.65E-13	54.30
rs880315	T	C	-0.508	0.032	9.59E-56	246.97
rs8904	A	G	0.306	0.031	1.71E-22	95.03
rs896312	T	C	0.194	0.032	1.60E-09	36.37
rs907612	T	C	0.319	0.032	3.83E-23	98.39
rs908591	A	G	-0.205	0.032	1.55E-10	40.94
rs908951	T	C	-0.226	0.032	7.14E-13	51.52
rs911547	A	G	-0.308	0.043	7.76E-13	51.44
rs912434	T	G	0.234	0.035	2.47E-11	44.44
rs917275	A	G	-0.173	0.031	3.26E-08	30.60
rs918948	T	C	-0.201	0.032	2.73E-10	39.82
rs927315	T	C	0.169	0.030	2.44E-08	31.07
rs9302885	A	G	0.224	0.030	1.03E-13	55.11
rs9311344	A	G	0.227	0.039	7.52E-09	33.33
rs932316	T	C	-0.223	0.038	6.45E-09	33.66
rs9349379	A	G	0.266	0.031	1.31E-17	72.91
rs9368222	A	C	0.228	0.034	1.84E-11	45.27
rs9394578	A	C	0.193	0.035	2.56E-08	30.97
rs9401913	A	G	0.520	0.031	3.66E-65	290.90
rs9449350	T	C	-0.219	0.032	1.19E-11	45.93
rs9486916	T	C	0.266	0.039	5.42E-12	47.63
rs9508495	T	C	-0.356	0.035	6.34E-24	101.54
rs9512650	A	G	-0.430	0.074	6.19E-09	33.81
rs9526707	A	G	-0.204	0.032	2.77E-10	39.85
rs9532243	A	C	0.224	0.030	8.17E-14	55.80
rs954767	A	C	-0.213	0.035	7.53E-10	37.79
rs9549328	T	C	0.292	0.036	7.91E-16	64.84
rs962369	T	C	0.223	0.033	1.43E-11	45.51

rs9634314	A	G	-0.392	0.057	4.43E-12	47.90
rs9652858	A	G	0.255	0.040	1.53E-10	40.91
rs9660067	A	G	0.267	0.033	2.52E-16	66.98
rs9661802	A	C	0.224	0.032	2.72E-12	48.87
rs9716164	T	C	0.180	0.031	3.40E-09	34.98
rs9747001	A	G	-0.265	0.037	6.85E-13	51.50
rs9869437	A	C	-0.200	0.032	3.22E-10	39.59
rs9875380	T	C	-0.175	0.030	6.53E-09	33.66
rs9880098	A	G	0.308	0.031	1.59E-23	100.06
rs9885632	T	C	0.236	0.034	4.37E-12	47.82
rs9886665	T	C	0.205	0.034	2.47E-09	35.65
rs994446	A	G	-0.347	0.037	1.87E-20	85.88
