# Supplementary material for: Identifying modifiable risk factors of lung cancer: Indications from Mendelian randomization
Source: PLoS One. 2021 Oct 18;16(10):e0258498. doi: 10.1371/journal.pone.0258498 (PMC8523078; doi:10.1371/journal.pone.0258498)
Supplement: S11 Table — The SNP is the result of genetic variants; A1 is the effect allele; A2 is the other allele; beta is the effect size of A1 on the exposure; she is the standard error of beta; pval is the p-value of beta; F is the F statistics. (PDF) [file pone.0258498.s024.pdf]

S11 Table: Instrumental variables of HDL cholesterol. SNP is the rsID of genetic variants; A1 is the effect allele; A2 is the other allele; beta is the effect size of A1 on the exposure; se is the standard error of beta; pval is the p value of beta; F is the F statistics.

SNP	A1	A2	beta	se	pval	F
rs10019888	A	G	0.027	0.005	4.90E-08	34.45
rs10282707	C	T	0.025	0.004	1.03E-11	51.02
rs10444840	G	A	0.020	0.004	2.05E-08	31.04
rs10750215	T	G	0.026	0.004	5.95E-14	55.18
rs10790166	G	A	0.030	0.006	1.27E-08	28.77
rs10892001	G	A	0.021	0.004	1.37E-08	34.35
rs10900221	A	G	0.025	0.004	1.00E-09	42.42
rs10951983	G	A	0.029	0.005	3.60E-10	41.53
rs11045163	G	A	0.022	0.004	3.20E-09	38.44
rs11057405	G	A	0.041	0.006	1.23E-09	44.16
rs11204072	C	T	0.034	0.004	1.28E-13	60.06
rs1129555	A	G	0.034	0.004	5.19E-17	79.59
rs11662909	A	G	0.035	0.005	4.63E-11	42.49
rs11665227	G	A	0.022	0.004	4.84E-08	36.67
rs11758326	A	G	0.034	0.006	1.40E-08	31.25
rs1180647	C	T	0.026	0.005	4.18E-08	30.02
rs11874381	A	G	0.035	0.005	5.52E-12	49.86
rs11875600	G	A	0.054	0.007	2.29E-12	53.25
rs12038699	A	G	0.022	0.004	3.98E-09	39.51
rs12145743	G	T	0.020	0.004	1.80E-08	31.80
rs12412743	C	T	0.029	0.005	1.31E-09	41.82
rs12521454	T	C	0.025	0.004	1.35E-09	41.09
rs12710745	A	G	0.022	0.004	2.91E-08	38.44
rs12720917	C	T	0.098	0.006	5.89E-68	314.90
rs12801636	A	G	0.024	0.004	3.15E-08	31.31
rs12908474	C	T	0.081	0.005	1.01E-55	235.88
rs12928598	A	G	0.038	0.004	2.48E-17	77.69
rs13076253	A	C	0.028	0.005	4.96E-09	34.76
rs13099479	A	G	0.036	0.006	1.82E-08	33.71
rs13107325	C	T	0.071	0.008	1.07E-15	82.39
rs13225097	A	G	0.023	0.004	4.33E-08	33.88
rs13312735	G	T	0.058	0.009	4.89E-12	46.40
rs13334864	T	C	0.057	0.007	1.83E-18	74.59
rs1531517	A	G	0.044	0.007	2.43E-08	35.35
rs17152302	T	C	0.022	0.004	3.96E-08	31.36
rs17162330	T	C	0.038	0.005	1.44E-13	60.14
rs17173637	T	C	0.036	0.006	1.90E-08	40.56

rs17380117	A	G	0.025	0.004	3.85E-09	36.29
rs174528	T	C	0.036	0.004	2.09E-24	108.16
rs17695224	G	A	0.029	0.004	2.42E-13	55.29
rs1801706	A	G	0.064	0.008	1.09E-15	73.50
rs1991515	T	C	0.023	0.004	5.62E-10	36.98
rs2233455	T	C	0.057	0.007	2.27E-14	73.80
rs2240466	A	G	0.039	0.005	1.01E-11	55.26
rs2256175	T	C	0.024	0.004	2.65E-09	37.87
rs2271051	A	G	0.049	0.007	1.60E-11	55.12
rs2278236	A	G	0.033	0.004	3.19E-18	89.44
rs2282231	C	T	0.034	0.006	2.10E-08	32.11
rs2412710	G	A	0.084	0.014	1.36E-09	36.52
rs2602836	A	G	0.019	0.003	4.96E-08	31.89
rs2606736	C	T	0.025	0.004	4.80E-08	32.73
rs2652840	C	T	0.027	0.004	1.09E-10	41.94
rs2777797	A	C	0.043	0.006	1.78E-11	50.15
rs289723	C	A	0.022	0.004	4.08E-09	33.52
rs2925979	C	T	0.035	0.004	1.32E-19	89.99
rs2967776	G	A	0.041	0.005	1.76E-13	61.02
rs2980885	A	G	0.035	0.004	1.73E-14	71.04
rs3184504	C	T	0.026	0.004	4.10E-12	51.36
rs333947	G	A	0.030	0.005	3.17E-09	39.66
rs3732361	A	G	0.020	0.004	2.54E-08	30.56
rs3775228	C	T	0.021	0.004	4.53E-09	36.69
rs3775380	A	G	0.024	0.003	1.11E-11	50.66
rs3780543	G	A	0.050	0.005	1.42E-19	89.71
rs3923113	C	A	0.023	0.004	2.46E-09	37.97
rs40271	C	T	0.023	0.004	2.47E-08	33.35
rs4075205	T	C	0.022	0.004	3.54E-09	40.96
rs4082919	T	G	0.024	0.004	5.74E-11	45.85
rs424346	T	C	0.068	0.011	4.84E-08	36.11
rs4362515	C	T	0.024	0.004	3.95E-08	29.87
rs4622454	T	C	0.032	0.004	1.93E-19	84.12
rs4752797	G	A	0.034	0.005	3.25E-10	47.58
rs4760278	A	C	0.027	0.004	4.36E-12	44.01
rs4784659	T	C	0.027	0.005	1.02E-08	31.27
rs486394	A	C	0.027	0.004	2.10E-10	49.00
rs4917014	G	T	0.022	0.004	1.03E-08	38.03
rs4970834	T	C	0.025	0.004	1.77E-08	33.32
rs499974	C	A	0.026	0.004	1.12E-08	35.73
rs5754100	T	C	0.034	0.006	3.86E-08	27.89
rs575442	T	C	0.032	0.004	1.03E-14	64.00
rs6031587	C	T	0.049	0.007	1.92E-09	43.49

rs605066	T	C	0.028	0.005	2.79E-08	32.89
rs6065904	G	A	0.052	0.006	1.95E-18	71.28
rs645040	G	T	0.031	0.004	1.53E-12	55.18
rs6488910	T	G	0.021	0.004	4.19E-08	32.43
rs6589567	C	A	0.039	0.006	2.18E-09	45.68
rs6668958	T	G	0.023	0.004	4.21E-08	30.25
rs6694102	G	A	0.030	0.005	1.69E-08	33.51
rs6857	C	T	0.067	0.008	2.63E-17	75.71
rs6971365	T	C	0.024	0.004	1.31E-10	38.24
rs7012891	T	C	0.023	0.004	2.13E-08	33.64
rs7073746	G	A	0.019	0.003	1.79E-08	30.90
rs7165301	T	C	0.056	0.005	3.84E-28	144.00
rs719726	C	T	0.019	0.004	4.86E-09	30.41
rs7244158	C	T	0.090	0.014	1.46E-11	42.82
rs7298751	G	A	0.043	0.005	2.46E-16	69.66
rs731839	A	G	0.022	0.004	3.44E-09	35.35
rs737337	T	C	0.057	0.006	4.56E-17	85.79
rs73959582	C	T	0.067	0.008	5.63E-17	80.76
rs7578326	G	A	0.028	0.004	2.50E-13	60.93
rs7613875	C	A	0.025	0.004	1.79E-11	49.39
rs767145	A	G	0.020	0.004	2.35E-08	31.80
rs783149	C	A	0.031	0.005	2.95E-11	46.30
rs7844579	T	C	0.022	0.003	1.03E-08	41.87
rs7932354	T	C	0.022	0.004	1.76E-08	32.11
rs7954519	T	G	0.039	0.005	1.99E-13	63.35
rs80164982	A	C	0.049	0.009	4.70E-08	32.60
rs8047395	G	A	0.019	0.004	2.88E-08	28.55
rs8071366	G	A	0.025	0.004	2.08E-09	34.31
rs820299	A	G	0.064	0.004	1.46E-67	301.07
rs838878	A	G	0.049	0.004	3.96E-30	141.09
rs863750	C	T	0.026	0.004	4.71E-13	56.89
rs9332412	G	A	0.021	0.003	1.87E-08	36.71
rs9897185	G	A	0.036	0.006	4.13E-08	31.64
rs9938618	G	A	0.058	0.008	1.44E-11	48.16
rs998584	C	A	0.026	0.004	2.27E-11	46.81
