# Supplementary material for: Identifying modifiable risk factors of lung cancer: Indications from Mendelian randomization
Source: PLoS One. 2021 Oct 18;16(10):e0258498. doi: 10.1371/journal.pone.0258498 (PMC8523078; doi:10.1371/journal.pone.0258498)
Supplement: S12 Table — The SNP is the result of genetic variants; A1 is the effect allele; A2 is the other allele; beta is the effect size of A1 on the exposure; she is the standard error of beta; pval is the p-value of beta; F is the F statistics. (PDF) [file pone.0258498.s025.pdf]

S12 Table: Instrumental variables of LDL cholesterol. SNP is the rsID of genetic variants; A1 is the effect allele; A2 is the other allele; beta is the effect size of A1 on the exposure; se is the standard error of beta; pval is the p value of beta; F is the F statistics.

SNP	A1	A2	beta	se	pval	F
rs10102164	A	G	0.032	0.005	3.74E-11	49.312
rs10128711	C	T	0.034	0.004	9.21E-13	59.711
rs10195252	T	C	0.024	0.004	3.81E-08	37.241
rs1030431	A	G	0.034	0.004	5.97E-17	81.950
rs1048699	T	C	0.032	0.006	1.81E-08	31.206
rs10495907	A	G	0.041	0.005	1.26E-13	60.960
rs11125936	T	C	0.037	0.006	4.07E-08	37.210
rs11216103	A	G	0.047	0.008	5.91E-10	37.758
rs11220437	C	T	0.034	0.006	5.76E-09	35.163
rs11244084	T	C	0.070	0.010	7.86E-11	44.787
rs112948675	T	G	0.062	0.011	2.70E-08	34.654
rs1129555	A	G	0.030	0.004	1.54E-13	57.381
rs11563251	T	C	0.035	0.006	4.50E-08	30.964
rs11650232	G	A	0.022	0.004	3.19E-08	32.111
rs11668536	C	T	0.051	0.005	4.30E-26	129.960
rs11679386	C	T	0.045	0.006	4.75E-14	54.906
rs11878377	C	T	0.055	0.006	4.13E-19	88.620
rs12067569	A	G	0.089	0.010	1.97E-17	78.323
rs1250229	C	T	0.024	0.004	3.13E-08	33.474
rs12625035	C	T	0.033	0.005	2.90E-11	43.296
rs12686004	G	A	0.036	0.006	2.25E-09	38.099
rs12720842	C	T	0.099	0.012	1.88E-15	73.280
rs12721109	G	A	0.446	0.018	2.99E-122	594.507
rs12753981	A	G	0.054	0.008	2.21E-09	45.394
rs12983082	C	A	0.051	0.006	1.52E-18	85.983
rs12983889	A	G	0.056	0.007	5.06E-15	67.820
rs13027716	T	G	0.059	0.011	4.84E-08	26.893
rs1337247	C	A	0.051	0.006	7.74E-17	70.840
rs1386585	T	C	0.034	0.005	2.21E-12	53.672
rs1408272	T	G	0.052	0.008	3.68E-09	39.251
rs1501910	G	A	0.035	0.005	1.44E-10	45.045
rs16872770	A	G	0.041	0.007	8.57E-09	32.538
rs16891156	C	A	0.097	0.017	8.23E-09	31.847
rs16988072	C	T	0.063	0.010	1.53E-09	38.149
rs17035630	A	G	0.051	0.006	1.44E-16	68.537
rs17120035	C	T	0.048	0.007	4.37E-11	44.075

rs17404153	G	T	0.034	0.005	1.83E-09	38.716
rs17508045	T	C	0.049	0.007	4.91E-12	54.670
rs17657025	T	C	0.035	0.007	4.40E-08	28.122
rs17677316	A	G	0.025	0.004	2.08E-09	34.306
rs1800961	C	T	0.069	0.011	6.03E-10	41.761
rs1801689	C	A	0.103	0.014	9.81E-12	54.696
rs2030746	T	C	0.021	0.004	8.61E-09	31.715
rs206070	A	G	0.026	0.005	4.63E-08	30.021
rs2088487	T	C	0.095	0.010	9.13E-18	89.218
rs2247056	C	T	0.025	0.004	1.42E-08	33.263
rs2294266	A	C	0.030	0.005	2.67E-08	34.142
rs2328223	C	A	0.030	0.005	5.63E-09	35.760
rs2385114	T	C	0.036	0.004	1.05E-19	84.734
rs2390536	A	G	0.022	0.004	2.04E-08	34.438
rs2495504	C	T	0.038	0.006	3.22E-10	41.920
rs2642438	G	A	0.035	0.004	7.32E-16	70.240
rs2647281	G	A	0.059	0.010	2.27E-09	38.440
rs2649999	T	C	0.041	0.006	1.12E-12	50.985
rs267733	A	G	0.033	0.005	5.29E-09	39.004
rs2737229	A	C	0.029	0.004	3.74E-12	55.675
rs2886232	T	C	0.045	0.006	3.88E-11	49.658
rs2902940	A	G	0.027	0.004	1.74E-11	44.662
rs2965174	G	A	0.025	0.004	5.21E-11	40.006
rs3093584	T	C	0.032	0.005	4.90E-09	37.634
rs3103353	T	C	0.034	0.006	3.13E-08	32.625
rs3184504	C	T	0.027	0.004	4.20E-12	49.740
rs364585	G	A	0.025	0.004	4.28E-10	42.937
rs3780181	A	G	0.045	0.007	1.76E-09	36.162
rs379309	C	T	0.031	0.004	1.39E-13	64.411
rs3798236	T	C	0.023	0.004	3.30E-08	35.693
rs3895886	T	C	0.042	0.006	1.02E-10	47.407
rs4253772	T	C	0.031	0.006	4.33E-08	27.214
rs4530754	A	G	0.028	0.004	3.58E-12	58.353
rs456598	A	G	0.047	0.006	2.00E-16	69.544
rs4704810	A	G	0.022	0.004	8.28E-09	32.610
rs4722551	C	T	0.039	0.005	3.95E-14	63.674
rs4783961	G	A	0.028	0.004	6.22E-12	53.521
rs4788597	C	T	0.033	0.004	2.11E-15	75.873
rs4823057	G	A	0.078	0.012	2.03E-08	39.264
rs487738	G	A	0.030	0.006	1.82E-08	26.576
rs499790	T	C	0.036	0.006	6.46E-09	37.854
rs516246	T	C	0.029	0.004	1.33E-13	55.675
rs603643	G	A	0.025	0.004	3.79E-09	37.823
rs631106	C	A	0.049	0.004	1.58E-31	148.84

						0
rs6415084	T	C	0.025	0.004	1.35E-10	45.654
rs6573778	T	C	0.030	0.004	3.33E-14	56.626
rs6756743	T	C	0.055	0.009	4.97E-09	36.131
rs676385	G	A	0.035	0.004	1.17E-15	67.775
rs6818397	T	G	0.022	0.004	1.68E-08	31.360
rs6857	T	C	0.192	0.008	5.12E-110	520.818
rs6917747	A	G	0.036	0.005	8.07E-10	44.940
rs7014582	G	A	0.034	0.006	2.43E-08	36.215
rs713286	T	C	0.047	0.007	4.50E-10	43.158
rs7225700	C	T	0.030	0.004	3.56E-13	61.087
rs7264396	C	T	0.025	0.005	4.41E-08	29.884
rs7469576	G	A	0.029	0.005	1.22E-08	37.261
rs7515901	C	T	0.049	0.005	4.28E-18	88.432
rs7604788	C	T	0.134	0.013	4.12E-22	103.853
rs7616006	A	G	0.025	0.004	2.54E-10	45.654
rs7640978	C	T	0.039	0.007	9.84E-09	32.276
rs7774197	C	A	0.051	0.007	2.97E-10	48.426
rs7798185	A	C	0.041	0.007	1.88E-09	38.402
rs780093	T	C	0.022	0.004	2.36E-08	36.325
rs7849542	A	G	0.024	0.004	1.12E-08	35.127
rs865774	C	T	0.035	0.006	2.76E-10	41.427
rs9932707	C	A	0.030	0.004	5.25E-14	63.568
rs9973305	C	T	0.053	0.009	1.01E-09	38.294
rs9989419	A	G	0.028	0.004	2.49E-12	50.446
