# Supplementary material for: Identifying modifiable risk factors of lung cancer: Indications from Mendelian randomization
Source: PLoS One. 2021 Oct 18;16(10):e0258498. doi: 10.1371/journal.pone.0258498 (PMC8523078; doi:10.1371/journal.pone.0258498)
Supplement: S13 Table — The SNP is the result of genetic variants; A1 is the effect allele; A2 is the other allele; beta is the effect size of A1 on the exposure; she is the standard error of beta; pval is the p-value of beta; F is the F statistics. (PDF) [file pone.0258498.s026.pdf]

S13 Table: Instrumental variables of total cholesterol. SNP is the rsID of genetic variants; A1 is the effect allele; A2 is the other allele; beta is the effect size of A1 on the exposure; se is the standard error of beta; pval is the p value of beta; F is the F statistics.

SNP	A1	A2	beta	se	pval	F
rs10088180	A	G	0.023	0.004	6.02E-10	32.49
rs10102164	A	G	0.030	0.004	4.60E-11	49.00
rs10128711	C	T	0.031	0.004	1.06E-11	50.97
rs10258022	G	A	0.024	0.004	9.89E-11	45.56
rs1030431	A	G	0.038	0.004	5.41E-22	104.37
rs10403668	G	A	0.039	0.005	2.41E-13	61.47
rs10495907	A	G	0.035	0.005	3.09E-11	47.89
rs10773003	A	G	0.037	0.006	4.08E-09	40.48
rs10774708	G	A	0.021	0.004	4.71E-08	36.34
rs1077514	T	C	0.030	0.005	6.40E-09	33.51
rs10900221	A	G	0.026	0.004	7.96E-09	38.68
rs11085752	A	C	0.034	0.005	2.53E-09	42.25
rs11650232	G	A	0.023	0.004	1.77E-09	38.98
rs11679386	C	T	0.039	0.006	2.23E-11	44.37
rs117733303	G	A	0.130	0.021	1.15E-08	37.42
rs11784762	G	A	0.033	0.005	4.02E-08	36.22
rs12412743	C	T	0.030	0.005	6.98E-10	40.20
rs12625035	C	T	0.028	0.005	3.62E-09	35.01
rs12660382	T	C	0.027	0.005	4.96E-09	33.44
rs12908474	C	T	0.040	0.006	6.09E-12	48.28
rs12921986	G	A	0.047	0.007	2.42E-10	44.70
rs13027175	G	T	0.065	0.011	2.23E-09	35.67
rs13075746	G	A	0.036	0.006	3.84E-08	32.97
rs1337247	C	A	0.038	0.006	4.76E-11	42.93
rs1384614	A	G	0.037	0.006	1.36E-08	32.88
rs138777	A	G	0.021	0.004	4.74E-08	33.45
rs1473886	G	T	0.024	0.004	5.06E-10	45.47
rs1501910	G	A	0.037	0.005	1.30E-12	54.46
rs1594895	T	C	0.026	0.005	3.18E-08	33.13
rs16979595	A	G	0.030	0.005	5.42E-09	35.05
rs17035630	A	G	0.040	0.006	2.16E-12	48.04
rs17120035	C	T	0.066	0.007	7.33E-22	88.90
rs17135399	G	A	0.051	0.008	7.39E-11	40.16
rs17398765	G	A	0.073	0.007	9.90E-24	101.10
rs17508045	T	C	0.039	0.006	1.13E-08	38.91
rs17651629	C	T	0.032	0.006	3.85E-08	30.16
rs1800562	G	A	0.057	0.008	1.91E-12	53.84
rs1800961	C	T	0.106	0.010	1.34E-24	110.56
rs1801701	T	C	0.050	0.006	8.30E-15	64.26

rs181360	T	G	0.028	0.004	7.32E-10	41.80
rs195517	G	A	0.031	0.005	3.23E-08	35.54
rs1997243	G	A	0.033	0.005	2.72E-10	44.09
rs2023472	G	A	0.021	0.004	4.21E-08	30.11
rs2030746	T	C	0.020	0.004	3.60E-08	28.93
rs2070959	A	G	0.021	0.004	3.52E-08	31.91
rs207150	C	T	0.035	0.006	2.96E-08	31.40
rs2141371	A	G	0.025	0.004	1.58E-08	34.59
rs2155216	T	C	0.090	0.015	5.51E-09	37.58
rs2156499	G	A	0.026	0.004	1.02E-10	45.13
rs2165537	T	G	0.090	0.013	1.61E-12	45.79
rs2249742	T	C	0.024	0.004	4.86E-10	39.23
rs2287019	C	T	0.029	0.005	4.02E-10	40.29
rs2287623	G	A	0.027	0.004	4.09E-12	57.51
rs2294261	A	C	0.025	0.004	2.37E-10	46.32
rs2385114	T	C	0.039	0.004	7.14E-24	103.18
rs2390536	A	G	0.022	0.004	7.74E-09	35.68
rs2495504	C	T	0.042	0.006	1.88E-12	54.81
rs2508015	G	A	0.024	0.004	9.55E-09	34.52
rs2642438	G	A	0.037	0.004	1.28E-18	85.56
rs2649999	T	C	0.040	0.006	6.11E-13	51.53
rs2737229	A	C	0.029	0.004	1.86E-13	58.24
rs2777797	A	C	0.039	0.006	1.13E-08	37.32
rs2792744	C	A	0.034	0.006	2.73E-09	31.92
rs2814982	C	T	0.044	0.006	3.68E-15	59.86
rs287227	G	T	0.065	0.007	2.72E-18	77.63
rs2886232	T	C	0.036	0.006	3.87E-08	33.34
rs2902940	A	G	0.024	0.004	8.84E-10	38.19
rs3091242	T	C	0.028	0.004	1.70E-13	62.23
rs3103353	T	C	0.036	0.006	3.28E-10	40.33
rs3184504	C	T	0.032	0.004	1.62E-17	73.87
rs3208856	C	T	0.203	0.019	1.09E-28	117.05
rs3739095	G	A	0.022	0.004	2.40E-09	36.33
rs3745157	T	C	0.025	0.004	2.15E-10	43.98
rs3780181	A	G	0.044	0.007	6.67E-10	38.76
rs3780542	A	G	0.049	0.008	2.35E-08	35.85
rs3809630	A	G	0.031	0.005	1.54E-08	35.10
rs3818441	G	A	0.028	0.004	2.94E-11	44.76
rs3895886	T	C	0.036	0.006	1.74E-08	37.44
rs4253772	T	C	0.032	0.006	9.85E-09	30.82
rs4530754	A	G	0.023	0.004	1.68E-09	42.44
rs456598	A	G	0.048	0.005	2.10E-18	80.00
rs4704810	A	G	0.022	0.004	2.89E-09	37.01
rs4722551	C	T	0.029	0.005	7.02E-09	38.07

rs4752805	G	A	0.025	0.004	1.62E-09	37.48
rs4803759	C	T	0.054	0.006	3.70E-21	85.72
rs487738	G	A	0.035	0.006	1.50E-11	38.35
rs4939593	T	C	0.025	0.004	5.39E-10	37.48
rs4968255	T	C	0.028	0.005	2.06E-08	32.82
rs499790	T	C	0.041	0.006	1.32E-13	52.24
rs5110	A	C	0.086	0.014	1.09E-09	38.57
rs516246	T	C	0.032	0.004	9.13E-17	72.48
rs56395424	G	A	0.032	0.005	1.03E-08	36.91
rs568052	A	G	0.033	0.006	8.92E-09	31.28
rs5930	G	A	0.041	0.004	1.24E-24	108.37
rs603643	G	A	0.023	0.004	1.45E-08	33.88
rs6413458	G	A	0.080	0.013	2.25E-09	35.82
rs6430552	T	C	0.024	0.004	1.44E-09	41.03
rs6435161	T	G	0.024	0.004	6.09E-09	38.82
rs646179	A	G	0.041	0.005	9.50E-16	71.39
rs6511727	T	G	0.025	0.004	1.17E-10	46.32
rs6573778	T	C	0.026	0.004	2.96E-11	45.48
rs6587970	G	A	0.029	0.005	1.24E-09	38.66
rs6589936	A	C	0.032	0.005	1.14E-09	37.14
rs6756743	T	C	0.055	0.009	1.82E-09	38.33
rs676385	G	A	0.025	0.004	1.44E-09	36.57
rs6818397	T	G	0.025	0.004	9.51E-11	42.42
rs6894099	C	T	0.056	0.010	8.51E-09	30.80
rs6917747	A	G	0.032	0.005	5.99E-09	38.82
rs7035671	C	T	0.036	0.005	7.66E-10	47.13
rs7080366	T	C	0.022	0.004	1.68E-08	34.40
rs714948	A	C	0.036	0.006	2.50E-10	36.80
rs7178779	G	T	0.023	0.004	3.76E-08	29.61
rs7197967	G	A	0.035	0.005	8.20E-10	44.61
rs7241687	G	A	0.024	0.004	9.80E-10	37.87
rs74444640	T	C	0.061	0.011	4.09E-09	33.75
rs7515901	C	T	0.041	0.005	5.84E-14	66.26
rs7604788	C	T	0.097	0.013	6.67E-15	59.97
rs7640978	C	T	0.038	0.007	1.66E-08	32.46
rs77301115	A	G	0.157	0.016	2.85E-20	99.49
rs7798185	A	C	0.040	0.006	3.54E-10	42.46
rs8176731	T	C	0.027	0.004	5.39E-11	44.01
rs865774	C	T	0.033	0.005	1.89E-09	38.30
rs892115	G	T	0.024	0.004	8.64E-09	35.70
rs9268882	C	T	0.035	0.006	1.91E-09	41.19
rs9282575	G	A	0.108	0.013	1.67E-15	70.67
rs9376090	T	C	0.025	0.004	2.60E-09	40.32
rs9972882	C	A	0.024	0.004	4.16E-08	36.91

rs9989419	G	A	0.032	0.004	1.03E-15	74.33
