# Supplementary material for: Identifying modifiable risk factors of lung cancer: Indications from Mendelian randomization
Source: PLoS One. 2021 Oct 18;16(10):e0258498. doi: 10.1371/journal.pone.0258498 (PMC8523078; doi:10.1371/journal.pone.0258498)
Supplement: S14 Table — The SNP is the result of genetic variants; A1 is the effect allele; A2 is the other allele; beta is the effect size of A1 on the exposure; she is the standard error of beta; pval is the p-value of beta; F is the F statistics. (PDF) [file pone.0258498.s027.pdf]

S14 Table: Instrumental variables of triglycerides. SNP is the rsID of genetic variants; A1 is the effect allele; A2 is the other allele; beta is the effect size of A1 on the exposure; se is the standard error of beta; pval is the p value of beta; F is the F statistics.

SNP	A1	A2	beta	se	pval	F
rs10088180	A	G	0.021	0.004	3.87E-08	32.52
rs10426094	C	T	0.024	0.004	8.47E-09	36.30
rs10440120	C	A	0.031	0.004	5.34E-11	48.37
rs10501321	T	C	0.022	0.004	1.41E-08	38.09
rs11172147	G	A	0.033	0.006	1.97E-09	34.92
rs1129555	G	A	0.021	0.004	6.72E-09	31.30
rs117604010	G	A	0.137	0.017	3.11E-15	65.04
rs117877390	C	T	0.110	0.014	1.53E-09	60.75
rs11879589	G	A	0.037	0.006	3.84E-11	40.92
rs12317176	T	C	0.024	0.004	1.23E-10	45.47
rs1240658	C	T	0.035	0.005	3.01E-12	56.58
rs12746810	A	G	0.037	0.006	2.33E-09	38.27
rs12994068	C	A	0.025	0.004	6.71E-10	41.57
rs16837533	A	G	0.024	0.005	3.87E-08	28.92
rs16842	C	T	0.027	0.004	6.33E-13	54.84
rs16885714	G	A	0.035	0.006	2.93E-08	34.59
rs17152302	C	T	0.023	0.004	2.99E-09	33.88
rs17410086	T	C	0.033	0.005	2.01E-09	36.45
rs174528	C	T	0.044	0.003	5.65E-36	164.44
rs1781930	G	A	0.031	0.004	2.51E-11	53.32
rs2068888	G	A	0.024	0.003	1.68E-11	50.24
rs2122823	T	C	0.024	0.004	8.61E-09	36.30
rs2284063	A	G	0.022	0.003	1.10E-09	39.99
rs2303223	G	A	0.020	0.003	3.50E-09	35.30
rs2304684	T	C	0.086	0.013	5.00E-11	45.86
rs2384629	A	G	0.128	0.013	1.03E-21	95.03
rs2412710	A	G	0.099	0.013	1.66E-11	56.02
rs2523729	G	A	0.033	0.006	3.00E-08	29.80
rs2869433	T	C	0.019	0.003	4.59E-08	31.89
rs2967776	A	G	0.032	0.005	1.80E-08	36.93
rs2976940	T	G	0.021	0.003	1.14E-08	36.35
rs2980885	G	A	0.058	0.004	3.00E-40	198.74
rs319	C	A	0.022	0.004	6.23E-09	32.61
rs3198697	C	T	0.020	0.003	2.21E-08	33.91
rs35133828	G	A	0.110	0.015	4.45E-09	52.09
rs3749946	A	C	0.045	0.007	4.19E-09	40.89
rs3760627	C	T	0.019	0.003	5.29E-09	30.90
rs3861397	G	A	0.024	0.004	1.08E-10	45.85
rs38855	A	G	0.019	0.003	2.11E-08	32.11

rs3923113	A	C	0.024	0.004	5.69E-11	45.08
rs4296389	C	T	0.056	0.005	2.21E-23	116.39
rs4711253	A	C	0.032	0.004	1.10E-11	56.42
rs4731702	C	T	0.018	0.003	2.19E-08	30.75
rs4738684	A	G	0.021	0.004	8.82E-09	34.31
rs4783961	G	A	0.021	0.004	1.29E-08	34.31
rs4803750	G	A	0.042	0.007	9.52E-09	36.52
rs4871598	A	G	0.044	0.004	5.12E-28	132.86
rs4871624	G	T	0.025	0.004	1.07E-11	47.13
rs4921667	A	G	0.031	0.005	6.00E-09	44.07
rs516226	T	C	0.067	0.007	6.22E-22	106.25
rs539298	A	G	0.019	0.003	3.60E-08	32.46
rs6029143	C	T	0.039	0.007	4.93E-08	29.86
rs6065904	A	G	0.040	0.006	4.70E-12	46.19
rs6066141	T	C	0.030	0.005	2.34E-08	31.40
rs62001736	A	G	0.034	0.005	5.55E-09	38.72
rs645040	T	G	0.029	0.004	1.83E-12	53.66
rs646179	A	G	0.036	0.005	3.80E-12	55.63
rs6587971	C	T	0.019	0.004	6.58E-09	25.26
rs6721762	C	T	0.074	0.009	1.35E-14	73.04
rs6831256	G	A	0.026	0.004	1.60E-12	54.34
rs6857	T	C	0.054	0.007	4.55E-19	60.57
rs7073746	A	G	0.025	0.003	1.23E-15	59.24
rs719726	T	C	0.020	0.004	2.49E-08	32.33
rs7254892	A	G	0.124	0.011	1.40E-24	135.74
rs72817536	A	G	0.039	0.006	1.66E-11	45.45
rs73012993	G	A	0.045	0.008	2.64E-08	29.98
rs731839	G	A	0.022	0.004	2.65E-09	38.72
rs7557068	G	A	0.036	0.006	8.04E-10	40.19
rs7578326	A	G	0.023	0.004	4.41E-10	43.94
rs7588926	C	T	0.038	0.005	1.39E-15	63.00
rs76881617	T	C	0.077	0.011	1.18E-10	51.12
rs7717984	T	C	0.025	0.004	1.52E-11	51.02
rs7943309	G	A	0.061	0.009	1.16E-11	48.36
rs8077889	C	A	0.025	0.004	9.88E-09	36.00
rs888246	T	C	0.071	0.006	8.99E-31	148.59
rs8891	T	C	0.025	0.004	1.16E-08	35.71
rs9989419	A	G	0.024	0.004	1.05E-11	48.20
