# Supplementary material for: Identifying modifiable risk factors of lung cancer: Indications from Mendelian randomization
Source: PLoS One. 2021 Oct 18;16(10):e0258498. doi: 10.1371/journal.pone.0258498 (PMC8523078; doi:10.1371/journal.pone.0258498)
Supplement: S15 Table — The SNP is the result of genetic variants; A1 is the effect allele; A2 is the other allele; beta is the effect size of A1 on the exposure; she is the standard error of beta; pval is the p-value of beta; F is the F statistics. (PDF) [file pone.0258498.s028.pdf]

S15 Table: Instrumental variables of HbA1c. SNP is the rsID of genetic variants; A1 is the effect allele; A2 is the other allele; beta is the effect size of A1 on the exposure; se is the standard error of beta; pval is the p value of beta; F is the F statistics.

SNP	A1	A2	Beta	se	pval	F
rs1046896	C	T	-0.028	0.002	4.46E-64	271.28
rs10774625	A	G	-0.009	0.002	1.46E-08	30.25
rs11248914	C	T	-0.014	0.002	2.56E-14	54.29
rs11603334	A	G	-0.012	0.002	6.85E-09	32.65
rs11708067	A	G	0.013	0.002	1.42E-12	46.81
rs11964178	A	G	0.010	0.002	6.38E-10	36.00
rs12368284	A	G	0.012	0.002	2.92E-10	36.00
rs12621844	C	T	-0.010	0.002	1.87E-08	30.25
rs13134327	A	G	0.013	0.002	2.64E-15	58.48
rs13266634	C	T	0.015	0.002	4.53E-20	77.85
rs1387153	C	T	-0.019	0.002	2.11E-24	100.00
rs1547247	A	G	-0.014	0.002	1.73E-17	67.82
rs17509001	C	T	0.018	0.002	1.94E-15	61.25
rs17533903	A	G	0.015	0.002	5.27E-12	46.49
rs17747324	C	T	0.015	0.002	6.12E-11	42.53
rs1800562	A	G	-0.04	0.004	4.67E-28	123.46
rs198846	A	G	-0.022	0.002	1.18E-23	100.00
rs2246434	A	G	0.019	0.002	1.99E-27	111.42
rs2383208	A	G	0.014	0.002	7.04E-12	44.44
rs267738	G	T	-0.011	0.002	2.59E-09	33.52
rs2979422	C	T	0.015	0.002	1.10E-10	42.53
rs3782123	A	C	-0.013	0.002	1.51E-10	42.25
rs423117	C	T	-0.019	0.003	1.30E-12	49.52
rs4607517	A	G	0.031	0.002	8.76E-38	166.84
rs4737009	A	G	0.021	0.002	4.48E-27	110.25
rs4745982	G	T	-0.095	0.006	2.87E-65	287.79
rs5030913	G	T	-0.013	0.002	3.56E-13	52.16
rs560887	C	T	0.028	0.002	1.48E-58	241.98
rs579459	C	T	0.011	0.002	9.42E-09	33.52
rs592423	A	C	0.009	0.002	3.96E-08	28.65
rs6474359	C	T	-0.044	0.005	1.50E-16	68.92
rs6980507	A	G	0.010	0.002	3.58E-08	29.04
rs7616006	A	G	0.010	0.002	5.07E-10	34.60
rs7756992	A	G	-0.012	0.002	2.80E-12	44.44
rs8192675	C	T	-0.011	0.002	1.38E-11	41.87
rs837763	C	T	-0.017	0.002	1.68E-28	112.89
rs855791	A	G	0.017	0.002	3.44E-28	112.89
rs9818758	A	G	0.012	0.002	7.74E-10	36.00
rs9914988	A	G	0.013	0.002	2.77E-11	42.25

rs9935401	A	G	0.010	0.002	1.87E-08	30.25
