# Supplementary material for: Identifying modifiable risk factors of lung cancer: Indications from Mendelian randomization
Source: PLoS One. 2021 Oct 18;16(10):e0258498. doi: 10.1371/journal.pone.0258498 (PMC8523078; doi:10.1371/journal.pone.0258498)
Supplement: S16 Table — The SNP is the result of genetic variants; A1 is the effect allele; A2 is the other allele; beta is the effect size of A1 on the exposure; she is the standard error of beta; pval is the p-value of beta; F is the F statistics. (PDF) [file pone.0258498.s029.pdf]

S16 Table: Instrumental variables of fasting glucose. SNP is the rsID of genetic variants; A1 is the effect allele; A2 is the other allele; beta is the effect size of A1 on the exposure; se is the standard error of beta; pval is the p value of beta; F is the F statistics.

SNP	A1	A2	beta	se	pval	F
rs10276674	C	T	-0.035	0.003	4.94E-28	120.67
rs10497347	C	T	-0.068	0.008	6.54E-16	65.36
rs11020155	T	C	-0.033	0.005	1.17E-10	41.52
rs11195502	T	C	0.033	0.004	1.66E-19	81.71
rs1128905	T	C	-0.014	0.002	2.26E-09	35.79
rs11558471	G	A	0.027	0.002	5.09E-35	152.65
rs11603334	A	G	0.020	0.003	5.52E-15	61.17
rs11607883	A	G	0.023	0.002	4.58E-30	129.92
rs11614702	G	A	0.030	0.004	1.68E-12	49.83
rs116983590	C	A	0.047	0.007	1.56E-10	40.95
rs11708067	G	A	0.023	0.002	4.71E-22	93.30
rs11919595	T	C	-0.025	0.004	9.75E-09	32.94
rs11924648	G	A	0.027	0.003	2.06E-19	81.30
rs12470752	C	T	0.036	0.006	1.05E-09	37.23
rs12888855	A	C	0.017	0.003	7.73E-12	46.89
rs13415496	A	G	-0.025	0.004	1.60E-10	40.98
rs16913693	G	T	0.044	0.006	5.98E-13	51.94
rs174576	A	C	0.019	0.002	4.55E-19	79.76
rs2232315	A	G	-0.076	0.012	7.85E-11	42.36
rs2383208	A	G	-0.023	0.003	2.41E-20	85.56
rs2715094	A	G	0.017	0.002	1.71E-14	58.91
rs2785137	A	G	0.012	0.002	4.97E-08	29.76
rs340874	T	C	0.016	0.002	1.34E-15	63.98
rs36080963	C	T	-0.046	0.008	2.04E-08	31.46
rs3778321	A	G	0.016	0.003	1.04E-09	37.31
rs3808320	T	C	0.051	0.008	1.10E-09	37.20
rs4502156	C	T	0.021	0.002	1.32E-24	105.03
rs4607517	A	G	-0.059	0.003	4.32E-97	437.36
rs4869272	T	C	-0.017	0.002	2.03E-16	67.69
rs560494	T	G	-0.024	0.003	2.12E-14	58.49
rs6048205	A	G	-0.034	0.005	8.07E-13	51.35
rs6598541	A	G	-0.012	0.002	1.04E-08	32.81
rs700548	A	G	-0.038	0.007	2.77E-08	30.91
rs7034200	C	A	0.016	0.002	1.28E-14	59.51
rs7142571	A	C	-0.016	0.003	1.94E-09	36.08
rs7178572	A	G	0.012	0.002	2.70E-08	30.95
rs7589456	T	C	0.053	0.009	2.88E-09	35.26
rs76323047	A	G	0.057	0.006	8.23E-20	83.00
rs76639975	G	A	-0.069	0.010	3.81E-11	43.71

rs77769873	A	G	0.067	0.012	5.34E-09	34.06
rs780093	T	C	0.031	0.002	4.87E-50	221.43
rs7903146	T	C	-0.024	0.002	6.32E-26	110.96
rs79621919	G	A	-0.083	0.013	5.25E-10	38.58
rs8044995	A	G	0.016	0.003	5.76E-09	33.95
rs8103278	A	G	-0.012	0.002	2.79E-08	30.89
rs853777	T	C	0.178	0.006	1.00E-200	929.26
rs9356744	C	T	-0.013	0.002	2.12E-09	35.92
rs954750	T	C	-0.022	0.003	4.54E-14	57.00
rs983309	T	G	-0.029	0.003	2.09E-21	90.38
