# Supplementary material for: Identifying modifiable risk factors of lung cancer: Indications from Mendelian randomization
Source: PLoS One. 2021 Oct 18;16(10):e0258498. doi: 10.1371/journal.pone.0258498 (PMC8523078; doi:10.1371/journal.pone.0258498)
Supplement: S18 Table — The SNP is the result of genetic variants; A1 is the effect allele; A2 is the other allele; beta is the effect size of A1 on the exposure; she is the standard error of beta; pval is the p-value of beta; F is the F statistics. (PDF) [file pone.0258498.s031.pdf]

**S18 Table: Instrumental variables of fasting insulin.** SNP is the rsID of genetic variants; A1 is the effect allele; A2 is the other allele; beta is the effect size of A1 on the exposure; se is the standard error of beta; pval is the p value of beta; F is the F statistics.

| SNP | A1 | A2 | beta | se | pval | F |
|---|---|---|---|---|---|---|
| rs11127048 | G | A | 0.034 | 0.005 | 6.87E-11 | 42.55 |
| rs1167834 | A | G | 0.032 | 0.005 | 1.84E-10 | 40.63 |
| rs2169387 | A | G | -0.050 | 0.008 | 4.99E-11 | 43.18 |
| rs28567725 | T | C | 0.035 | 0.005 | 5.71E-12 | 47.43 |
| rs7985878 | C | T | -0.027 | 0.005 | 4.88E-08 | 29.76 |
